# Safety and Immunogenicity of an AMA1 Malaria Vaccine in Malian Children: Results of a Phase 1 Randomized Controlled Trial

**DOI:** 10.1371/journal.pone.0009041

**Published:** 2010-02-04

**Authors:** Mahamadou A. Thera, Ogobara K. Doumbo, Drissa Coulibaly, Matthew B. Laurens, Abdoulaye K. Kone, Ando B. Guindo, Karim Traore, Mady Sissoko, Dapa A. Diallo, Issa Diarra, Bourema Kouriba, Modibo Daou, Amagana Dolo, Mounirou Baby, Mahamadou S. Sissoko, Issaka Sagara, Amadou Niangaly, Idrissa Traore, Ally Olotu, Olivier Godeaux, Amanda Leach, Marie-Claude Dubois, W. Ripley Ballou, Joe Cohen, Darby Thompson, Tina Dube, Lorraine Soisson, Carter L. Diggs, Shannon L. Takala, Kirsten E. Lyke, Brent House, David E. Lanar, Sheetij Dutta, D. Gray Heppner, Christopher V. Plowe

**Affiliations:** 1 Malaria Research and Training Center, University of Bamako, Bamako, Mali; 2 Howard Hughes Medical Institute/Center for Vaccine Development, University of Maryland School of Medicine, Baltimore, Maryland, United States of America; 3 GlaxoSmithKline Biologicals, Rixensart, Belgium; 4 EMMES Corporation, Rockville, Maryland, United States of America; 5 Malaria Vaccine Development Program, U.S. Agency for International Development, Washington, D.C., United States of America; 6 Division of Malaria Vaccine Development, Walter Reed Army Institute of Research, Silver Spring, Maryland, United States of America; Walter and Eliza Hall Institute of Medical Research, Australia

## Abstract

**Background:**

The objective was to evaluate the safety and immunogenicity of the AMA1-based malaria vaccine FMP2.1/AS02_A_ in children exposed to seasonal falciparum malaria.

**Methodology/Principal Findings:**

A Phase 1 double blind randomized controlled dose escalation trial was conducted in Bandiagara, Mali, West Africa, a rural town with intense seasonal transmission of *Plasmodium falciparum* malaria. The malaria vaccine FMP2.1/AS02_A_ is a recombinant protein (FMP2.1) based on apical membrane antigen 1 (AMA1) from the 3D7 clone of *P. falciparum*, formulated in the Adjuvant System AS02_A_. The comparator vaccine was a cell-culture rabies virus vaccine (Rab*Avert*®). One hundred healthy Malian children aged 1–6 years were recruited into 3 cohorts and randomized to receive either 10 µg FMP2.1 in 0.1 mL AS02_A_, or 25 µg FMP2.1 in 0.25 mL AS02_A_, or 50 µg FMP2.1 50 µg in 0.5 mL AS02_A_, or rabies vaccine. Three doses of vaccine were given at 0, 1 and 2 months, and children were followed for 1 year. Solicited symptoms were assessed for 7 days and unsolicited symptoms for 30 days after each vaccination. Serious adverse events were assessed throughout the study. Transient local pain and swelling were common and more frequent in all malaria vaccine dosage groups than in the comparator group, but were acceptable to parents of participants. Levels of anti-AMA1 antibodies measured by ELISA increased significantly (at least 100-fold compared to baseline) in all 3 malaria vaccine groups, and remained high during the year of follow up.

**Conclusion/Significance:**

The FMP2.1/AS02_A_ vaccine had a good safety profile, was well-tolerated, and induced high and sustained antibody levels in malaria-exposed children. This malaria vaccine is being evaluated in a Phase 2 efficacy trial in children at this site.

**Trial Registration:**

ClinicalTrials.gov NCT00358332 [NCT00358332]

## Introduction

A safe and effective malaria vaccine would be a major addition to current malaria control tools and could reinforce hope for malaria eradication. The *Plasmodium falciparum* apical membrane antigen 1 (AMA1) is considered to be a promising antigen for blood stage vaccine development based on evidence that it plays a critical role during merozoite invasion of erythrocytes and that this function can be abrogated with inhibitory antibodies [1]–[5], and on sero-epidemiological studies showing association of anti-AMA1 antibodies with naturally acquired protection against malaria [6], [7]. A vaccine that boosts levels of anti-AMA1 antibodies might therefore reduce the risk that malaria infection will cause clinical disease, making AMA1 an attractive candidate for inclusion in a multi-stage, multi-antigen malaria vaccine [8].

AMA1 is highly polymorphic–more than 300 unique AMA1 haplotypes have been identified worldwide and more than 200 at a single site in Mali [9]. This extreme genetic diversity presumably results from balancing selection driven by host immunity. In vitro [10] and animal studies [4], [11], [12] have suggested the possibility of strain-specific immunity, raising concern that AMA1 vaccines based on one or a few alleles might not provide broad protection [13]. However, both in vitro [14] and molecular epidemiological [9], [15] studies have suggested possible diversity-covering approaches to developing effective AMA1 vaccines.

Three AMA1-based adjuvanted protein vaccines have been evaluated in clinical trials in Mali, including two different monovalent vaccines based on AMA1 derived from the 3D7 and FVO clones of *P. falciparum*, respectively, [12], [16], [17] and a bivalent vaccine that includes both the 3D7 and FVO versions of AMA1 adjuvanted with aluminum hydroxide [18]–[20]. The failure of this bivalent AMA1 vaccine to protect children in a Phase 2 trial in Mali [20] may be due to the relatively modest and short-lived nature of the antibody responses generated by the vaccine and/or to the inability to overcome genetic diversity.

FMP2.1 is a lyophilized preparation of the ectodomain of the 3D7 clone of *P. falciparum* AMA1 [21]. A Phase 1 study in malaria-naïve North American volunteers found that the AMA1-based vaccine FMP2.1/AS02_A_ elicited potent humoral and cellular immune responses and that immune sera recognized sporozoites and merozoites by immunofluorescence assay and inhibited both parasite growth and AMA1 processing in homologous 3D7 parasites [16]. The first Phase 1 study of this vaccine in a malaria-exposed population found it to have promising safety and tolerability profiles in adults in Bandiagara, Mali, and to elicit dose-dependent anti-AMA1 antibody responses [17] as well as IL-5 production and lymphocyte proliferative responses [22].

The overall objective of the current study was to identify an optimal pediatric dose of FMP2.1/AS02_A_ that is safe, with high immunogenicity and acceptable reactogenicity, for progression to efficacy testing. The safety and reactogenicity of FMP2.1/AS02_A_, as well as the magnitude and duration of the antibody response, were evaluated in children naturally exposed to *P. falciparum* infection.

## Methods

The protocol and supporting CONSORT checklist are available as supporting information; see Protocol S1 and Checklist S1.

### Study Setting

The study was conducted at the Bandiagara Malaria Project research clinic adjacent to the district hospital in Bandiagara, a rural town of 13,634 inhabitants in the Dogon Country in northeast Mali. Bandiagara is relatively dry, with a mean annual rainfall of 600 mm. *Anopheles gambiae* is the principal malaria vector. Malaria transmission is highly seasonal, with minimal transmission at the height of the dry season in March; less than one infected bite per person per month as the transmission season starts and ends in June and December, respectively; and a peak of up to 60 infected mosquito bites per person per month in August or September [23], [24]. *P. falciparum* represents 97% of malaria infections with 3% due to *P. malariae* and rare infections with *P. ovale*. Despite the seasonal transmission pattern, the malaria burden is heavy: children aged less than 10 years have an average of 2 clinical malaria episodes every transmission season [24], and severe malaria afflicts 1 in 50 children aged less than 6 years each year [23]. Older children and adults are relatively protected against malaria disease, but remain susceptible to infection.

### Participants

After obtaining community permission as described by Diallo et al. [25], the trial was publicized by local radio broadcast. Parents were invited to bring children aged 1 to 6 years to the research clinic to be screened for eligibility. Children were eligible for inclusion if they planned to remain in Bandiagara for at least 12 months and if their parents or guardians gave written informed consent. Exclusion criteria included: significant current illness as indicated by history, examination and/or laboratory testing including complete blood counts, alanine aminotransferase (ALT) and serum creatinine; previous immunization with a rabies vaccine or any experimental vaccine; chronic use of immunosuppressants; receipt of blood products during the previous 6 months; and allergy to substances present in the vaccines.

### Ethics

The trial was conducted in compliance with the International Conference on Harmonisation Good Clinical Practices, the Declaration of Helsinki and regulatory requirements of Mali. The protocol was approved by institutional review boards of the University of Bamako Faculty of Medicine, University of Maryland Baltimore, and the U.S. Army Surgeon General. Separate written informed consent was obtained for screening and enrollment. Verbal consent of illiterate parents or guardians was administered and then documented using their thumbprints, a process verified by signatures of independent witnesses. Permission to import and administer the investigational products in Mali was granted by the Republic of Mali Ministry of Health. The trial was monitored by the National Institute of Allergy and Infectious Diseases, Division of Microbiology and Infectious Diseases and the United States Army Medical Material Development Activity.

### Interventions

The FMP2.1 antigen (Lot 1046) is comprised of amino acids #83-531 corresponding to the ectodomain of AMA1 derived from the 3D7 clone of *P. falciparum*. The protein was produced in and purified from *E. coli* bacteria under current Good Manufacturing Practices (cGMP) at the Walter Reed Army Institute of Research Pilot Bioproduction Facility (Forest Glen, Maryland, United States) [21]. The vaccine was provided in vials containing approximately 50 µg of lyophilized protein.

The AS02_A_ Adjuvant System is composed of an oil-in-water emulsion and 2 immuno-stimulants, 3-deacylated monophosphoryl lipid A and QS21, a saponin agent derived from the soap bark tree, *Quillaja saponaria* [26], [27]. AS02_A_ was manufactured by GlaxoSmithKline Biologicals (Rixensart, Belgium) according to cGMP and provided in pre-filled syringes. The whole content of each FMP2.1 vial was dissolved by adding the contents of 0.62 mL pre-filled syringes of AS02_A_ to the vial immediately before injection, mixing well, and withdrawing into a new syringe. The RabAvert® rabies vaccine (Chiron Corporation, Emeryville, California, United States) is a sterile freeze-dried vaccine obtained by growing the fixed-virus strain Flury LEP in primary cultures of chicken fibroblasts. It is supplied in pre-filled syringes containing lyophilized antigen to which 1 mL of sterile water was added as diluent before injection. All doses of all vaccines were administered by intramuscular injection preferably in the left deltoid muscle.

One hundred children aged 1 to 6 years were sequentially assigned to 3 cohorts of 20, 40 and 40 participants with stratification for age by 2-year increments (1–2 years, 3–4 years, and 5–6 years) to ensure that the study groups were balanced by age in case of age-related differences in tolerability or immunogenicity. Within each cohort, participants were randomized in a 3∶1 fashion to receive 10, 25 or 50 μg of FMP2.1 adjuvanted with a proportionate volume of AS02_A_, or rabies vaccine. After reconstitution, the doses of FMP2.1/AS02_A_ were approximately: 10 µg of FMP2.1 in a final volume of 0.10 mL AS02_A_ in Cohort 1, 25 µg of FMP2.1 in a final volume of 0.25 mL AS02_A_ in Cohort 2, and 50 µg FMP2.1 in a final volume of 0.5 mL in Cohort 3. Because the final injection volumes were slightly smaller than the reconstitution volumes, the doses of the FMP2.1 antigen delivered were slightly less than 10, 25 or 50 μg. Vaccines were given on a 0-, 1- and 2-month schedule. In each cohort, older children were immunized at least one day before younger children so that vaccine responses could be observed first in older children before exposing younger children to potential risks of vaccination. The first vaccination was given in early November 2006 near the end of the peak malaria transmission season; the second and the third doses were given in December - February 2007, when malaria transmission typically declines to virtually undetectable levels at this site. The final study follow-up visit on day 364 coincided with the end of the 2007 malaria transmission season. The cohorts were immunized in a staggered fashion to permit interim safety analyses; each successive immunization of Cohort 1 was followed in approximately 3 weeks by the corresponding immunization of Cohort 2. Immunizations of Cohort 2 were followed in a similar way by immunizations of Cohort 3. Two interim safety analyses were reviewed by an independent Data and Safety Monitoring Board, which provided written recommendations to proceed before each of the first immunizations of Cohorts 2 and 3.

### Objectives

The primary objective was to evaluate the safety and reactogenicity of 3 injections of 3 different dose levels of the malaria vaccine FMP2.1/AS02_A_ in malaria-experienced Malian children. The secondary objective was to measure the magnitude and duration of antibody responses to FMP2.1. Exploratory objectives include measuring vaccine-induced cellular immune responses at baseline and after immunization (results to be presented elsewhere).

### Outcomes

The primary outcome was safety, measured as 1) occurrence of solicited symptoms during a 7-day follow-up period after immunization (day of immunization and days 1, 2, 3 and 7 after immunization); 2) occurrence of unsolicited symptoms during a 30-day follow-up period after each immunization (day of immunization and 29 subsequent days); and 3) occurrence of serious adverse events (SAE) during the study period. Secondary outcome measures include serum antibody levels and activity of anti-FMP2.1 measured against recombinant 3D7 AMA1 at baseline and at specified times during and after immunization.

#### Assessment of safety and tolerability

Following each immunization, participants were directly observed for 60 minutes, then evaluated at the study clinic 1, 2, 3, 7, 14 and 30 days after each immunization and on study days 120, 180, 272 and 364. Starting on day 180, monthly home visits were made to check the health status of participants and to encourage parents or guardians to bring them to the research clinic if they felt ill. Study physicians were available at the research clinic at all times throughout the 12-month study period to assess and treat adverse events.

Clinical evaluations consisted of measurement of vital signs and assessment for local injection site and general solicited signs or symptoms. Local signs and solicited symptoms included pain or tenderness, swelling, and erythema at the injection site. General signs and solicited symptoms included fever (oral temperature ≥37.5°C), vomiting, irritability/fussiness, drowsiness and loss of appetite. Any other signs or symptoms were considered to be unsolicited, as were signs or symptoms that occurred more than 7 days after immunization. Solicited symptoms were considered to be related to the study vaccines. Unsolicited signs and symptoms were recorded during the 30 days after each immunization, whereas SAEs were monitored throughout the 12-month study period.

Blood was collected at screening, on immunization days, 7 days after each immunization and on study days 90, 180, 272 and 364 to determine complete blood count, ALT and serum creatinine. Although clinical malaria episodes were not formally assessed as a study endpoint, malaria microscopy was performed for diagnostic purposes whenever participants presented with symptoms suggestive of malaria.

Adverse events were graded by severity and judged for potential association to study vaccines. Solicited adverse events were graded according to the system outlined in Table 1. Other non-laboratory adverse events were classified as grade 1–3 adverse events. Grade 1 adverse events were easily tolerated, causing minimal discomfort and not interfering with daily activities. Grade 2 adverse events were sufficiently discomforting to interfere with normal activities. Grade 3 adverse events prevented normal daily activities. For laboratory tests, toxicity grading was assigned using normal reference ranges based on a similar local pediatric population with the exception of absolute lymphocyte counts, which were based on normal values in Ugandan children [28].

**Table 1:** Assessment of Solicited Adverse Event (AE) Intensity.

Solicited AE	Grade	Intensity Definition
Pain/tenderness at injection site	0	Absent
	1	Minor reaction to touch
	2	Cries/protests on touch
	3	Cries when limb is moved/spontaneously painful
Swelling at injection site	0	Absent
	1	<5 mm
	2	5–20 mm
	3	>20 mm
Erythema at injection site	0	Absent
	1	<5 mm
	2	5–20 mm
	3	>20 mm
Limitation of arm motion/shoulder abduction	0	None
	1	>90° but <120°
	2	>30° but ≤90°
	3	≤30°
Fever	0	<37.5°C
	1	37.5–38.0°C
	2	38.1–39.0°C
	3	>39.0°C
Irritability/fussiness	0	Behavior as usual
	1	Crying more than usual/no effect on normal activity
	2	Crying more than usual/interferes with normal activity
	3	Crying that cannot be comforted/prevents normal activity
Drowsiness	0	Behavior as usual
	1	Drowsiness easily tolerated
	2	Drowsiness that interferes with normal activity
	3	Drowsiness that prevents normal activity
Loss of appetite	0	Normal
	1	Eating less than usual/no effect on normal activity
	2	Eating less than usual/interferes with normal activity
	3	Not eating at all
Vomiting	0	Absent
	1	Occasional but able to eat/drink normal amounts
	2	Repeated with limited oral intake
	3	Continuous, unable to keep down liquids or solids

#### Antibody responses to AMA1

Antibody levels (µg/mL) measuring total IgG against the *P. falciparum* 3D7 AMA1 vaccine antigen were measured by an enzyme-linked immunosorbent assay (ELISA) [16]. Briefly, plates were coated overnight at 4°C with the FMP2.1 recombinant AMA1 antigen (100 µL/well, 0.5 µg/mL), after which they were blocked with a 0.5% boiled casein buffer for 1 hour at 22°C. Test samples were added to the plate, serially diluted in 8 sequential 2-fold serial dilutions (done in triplicate) and incubated for 2 hours at 22°C. Secondary antibody (Affinity Purified Antibody Peroxidase Labeled Goat Anti-Human IgG (γ), KPL, Gaithersburg, Maryland, United States: Cat#074-1002) at a 1∶4,000 dilution, was added and incubated for 1 hour at 22°C, after which substrate (ABTS Peroxidase Substrate System (2-Component), KPL: Cat#50-62-01) was added and incubated for an additional hour at 22°C. A stop solution (20% SDS) was added and the plates were read using a Spectromax 340PC Plate Reader (Molecular Devices, Sunnyvale, California, United States). Between each incubation step the wells were washed in PBS using a SkanWasher Plate Washer (Molecular Devices) with four washing cycles of 400 µl each. Antibody responses were measured on serum obtained from participants at the time of each immunization (study days 0 [baseline], 30 and 60), and 1, 4, 7 and 10 months after the scheduled time of the last immunization (study days 90, 180, 272 and 364).

### Sample Size

The sample size of 15 in the 10 µg dose group and 30 each in the 25 µg and 50 µg dose groups was chosen to balance the need to detect any possible untoward reactions against the need to limit the number of volunteers exposed to an experimental vaccine for evaluation of safety. This Phase 1 trial was thus not powered to detect differences between groups. We used a comparator vaccine group of 25 to permit broad estimates of the incidence of local and general side effects and of immune responses among vaccine recipients compared to controls. We began with a group of 20 children randomized 3∶1 to receive 10 µg of FMP2.1 in 0.1 mL of AS02_A_ or rabies vaccine to rule out a common adverse reaction in children that would preclude further testing. This was not considered a dose level to be evaluated for further clinical development and thus did not warrant the larger sample size of the 25 µg and 50 µg dose level groups.

### Randomization—Sequence Generation

Participants were randomized to one of 3 cohorts in the order of enrollment, with stratification for age by 2-year increments (1–2 years, 3–4 years, 5–6 years). Within each of the 3 cohorts, individual participants were randomized in a 3∶1 ratio to receive either FMP2.1/AS02_A_ or rabies vaccine. The randomization sequence was generated by a computer program to ensure a 3∶1 ratio of vaccine allocation. Randomly generated sequential codes linked each study number to a vaccine assignment (FMP2.1/AS02_A_ or rabies vaccine).

### Randomization—Allocation Concealment

The randomization sequence was provided by the study statistician in an opaque sealed envelope to the study pharmacists. In addition the local safety monitor was provided with a sealed envelope to be opened if it was deemed necessary to determine urgently the intervention a participant had received; no such emergency unblinding occurred. The only people at the study site with access to the randomization codes during the study were the 2 study pharmacists, who had no contact with study participants and did not reveal vaccine assignments to anyone else. Study participants and investigators who assessed outcomes were blinded to vaccine assignment.

### Randomization—Implementation

Clinical investigators assigned study numbers to participants of each group in the order in which they arrived at the clinic on the first day of immunization. On this day, study pharmacists opened the sealed envelope containing the vaccine assignments and prepared the vaccine to be administered to the respective study participant. The vaccine and dose assigned during the first immunization were maintained for second and third immunizations. The study pharmacists prepared the vaccines in a special room with access strictly limited to them and to study monitors. Syringes containing the prepared vaccines were passed through small sliding doors from the vaccine preparation room to separate, private vaccine administration rooms, where the immunizations were administered.

### Blinding

The reconstituted rabies vaccine was a clear to slightly opaque, colorless suspension of 1 mL volume, while FMP2.1/AS02_A_ was off-white and 0.10, 0.25 or 0.5 mL in volume. Syringes containing vaccines were covered with opaque tape to conceal their contents from participants and immunizers. The study pharmacists, who were unblinded, had no study-related contact with participants and were not involved in outcome assessment. Because of the difference in volumes, the immunizers could potentially have deduced which vaccine was given to a specific participant, and therefore they did not participate in other study procedures, including follow-up assessments. The presence of both study pharmacists and immunizers at the site was limited to the periods during which immunizations were given, and these individuals did not discuss vaccine allocation with other study staff.

### Statistical Methods

Statistical analyses were performed using SAS version 9.2 (SAS, Cary, North Carolina, United States). Confidence intervals for geometric mean AMA1 antibody levels (µg/mL) were estimated by using log_10_-transformed values, calculating the 95% confidence interval based on the normal distribution, and then converting the limits to the original scale for presentation. T-tests were used to compare log-transformed antibody levels at each study time point. Longitudinal mixed models were also used to estimate the effect of vaccine dose on mean log-transformed antibody levels over time, using a spatial exponential covariance structure to model the correlation between measurements from the same individual while taking into account the number of study days between measurements. Safety and immunogenicity analyses were based on intention-to-treat, such that all available data were included in analyses.

## Results

### Participant Flow

**Figure 1:**
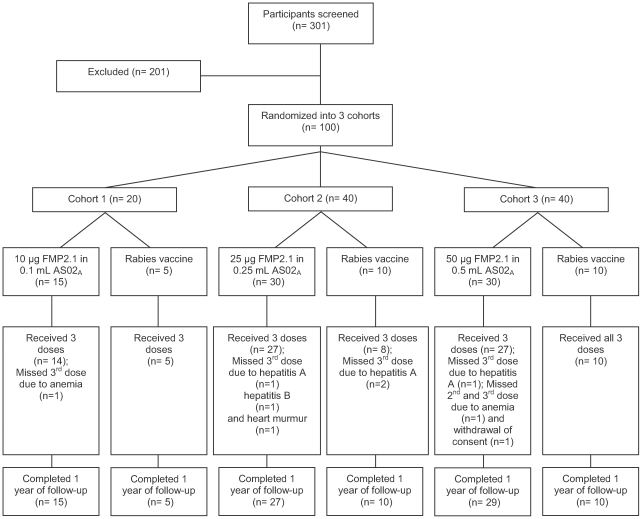
Trial profile.

Three hundred and one (301) children were screened, and 100 who fulfilled the criteria for inclusion were enrolled in the study (Figure 1). The most common reasons for exclusion were medical illnesses such as anemia, respiratory infections and malaria. Nine parents of screened children subsequently declined to allow their children to participate in the study. In Cohort 1, one participant missed the third vaccine dose due to an episode of anemia. In Cohort 2, the third dose was not given to 5 children due to: hepatitis B infection in one child, hepatitis A infections in 3 children and a new asymptomatic systolic heart murmur in another child, further assessments of whom uncovered no evidence of cardiac disease. In Cohort 3, one child had hepatitis A and was not given the third vaccine dose. In the same cohort, 2 children missed the second and third vaccine doses, in one case because of anemia diagnosed on the day that the second dose was due, and in the other case, because the participant's father withdrew consent for blood collection after first vaccination. Children who missed vaccine doses continued to be followed throughout the duration of the study.

### Recruitment and Surveillance

Participants were recruited between October 28 and November 30, 2006. Immunizations for Cohort 1 began on November 3, 2006, for Cohort 2 on November 23, 2006, and for Cohort 3 on December 13, 2006. Subsequent immunizations were done at 30-day intervals following this staggered start. Active surveillance of participants for 30 days after each immunization was completed in March 2007, corresponding to study day 90. The database was locked for the primary unblinded analysis after study day 90 so that results could be used to plan a Phase 2 trial, and the study continued in a single-blinded fashion, although individual study allocations were not disclosed to on-site study investigators or staff, with the exception of the principal investigator. The extended surveillance phase included continuous free access to basic medical care at the research clinic, monthly home visits, and scheduled visits on study days 180, 272 and 364. Of the 100 children enrolled, 96 completed the follow-up schedule.

### Baseline Data

The four study groups were similar at enrollment with regard to gender, age or laboratory parameters (Table 2). Forty-nine of 100 participants were female.

**Table 2:** Baseline Characteristics of FMP2.1/AS02_A_ and Rabies Vaccine groups.

Characteristics	FMP2.1/AS02_A_ 10 µg	FMP2.1/AS02_A_ 25 µg	FMP2.1/AS02_A_ 50 µg	Rabies Vaccine
	n = 15	n = 30	n = 30	n = 25
Mean age in year (SD)	3.5 (1.8)	3.5 (1.7)	3.6 (1.7)	3.2 (1.9)
Number of Females (%)	7 (46.7)	15 (50.0)	12 (40.0)	15 (60.0)
Mean WBC x 10^3^/µL (SD)	9.47 (3.67)	8.83 (2.20)	9.53 (2.59)	9.62 (3.02)
Mean hemoglobin g/dL (SD)	10.9 (1.2)	11.0 (1.0)	11.0 (1.1)	10.6 (0.8)
Mean platelets x 10^3^/µL (SD)	448 (129)	422 (152)	410 (151)	423 (132)
Mean lymphocytes x 10^3^/µL (SD)	5.03 (2.02)	4.84 (1.64)	4.75 (1.75)	5.26 (1.96)
Mean creatinine **µ**M/L (SD)	44.1 (0.0)	44.1 (0.0)	44.3 (1.3)	44.1 (0.0)
Mean ALT U/L (SD)	13.53 (5.34)	20.10 (21.82)	18.23 (8.55)	15.44 (6.92)
GMT Anti-AMA-1 antibody titer	532	500	1,088	456
(95% CI)	(97-2,922)	(168-1,489)	(448-2,645)	(151-1,377)

GMT, geometric mean titer; CI, confidence interval; ALT, alanine aminotransferase; SD, standard deviation.

### Numbers Analyzed

All available data from all participants, including partial data from participants lost to follow-up, were included in both safety and immunogenicity analyses.

### Safety and Reactogenicity

#### Local solicited adverse events

**Figure 2:**
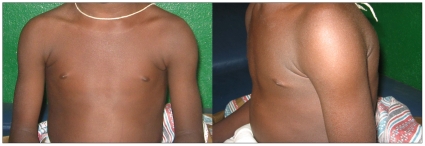
Grade 3 injection site swelling 1–2 days following immunization with the malaria vaccine. Injection site swelling was considered grade 3 if it exceeded 20 mm in its widest dimension. Grade 3 swelling was typically unnoticed by participants and parents and came to attention only during physical examinations. It was transient, lasting 1–3 days, and not associated with functional impairment. Shown here is grade 3 injection site swelling of the left shoulder of a study participant.

Injection site swelling and pain were the most common local solicited adverse events reported during the 7 day post-immunization period (Tables 3 and 4). The study was not powered for statistical comparisons of event rates between groups, but the frequency and severity of these local events tended to decrease with successive immunizations, especially in the 10 µg group. The proportion of children who experienced local pain or swelling was higher in the malaria vaccine groups compared to the rabies vaccine group. Grade 3 local adverse events consisted mainly of injection site swelling (Figure 2), which was reported in all study groups, but had a higher frequency in the 50 µg malaria vaccine group. Grade 3 local swelling was generally associated with minor injection site pain. Other grade 3 local reactions consisted of injection site pain and erythema. Grade 3 pain was always associated with grade 3 injection site swelling. One participant experienced grade 3 local erythema that was associated with grade 3 injection site swelling with no report of local pain.

**Table 3:** Signs and Solicited Symptoms during 7-Day Follow-Up Periods after Each Immunization: Malaria Vaccine by Dose Regimen.

	FMP2.1/AS02_A_ 10 µg						FMP2.1/AS02_A_ 25 µg						FMP2.1/AS02_A_ 50 µg					
	Immunization 1		Immunization 2		Immunization 3		Immunization 1		Immunization 2		Immunization 3		Immunization 1		Immunization 2		Immunization 3	
	n = 15		n = 15		n = 14		n = 30		n = 30		n = 27		n = 30		n = 28		n = 27	
	Overall	Severe	Overall	Severe	Overall	Severe	Overall	Severe	Overall	Severe	Overall	Severe	Overall	Severe	Overall	Severe	Overall	Severe
	n (%)	n (%)	n (%)	n (%)	n (%)	n (%)	n (%)	n (%)	n (%)	n (%)	n (%)	n (%)	n (%)	n (%)	n (%)	n (%)	n (%)	n (%)
Local																		
Pain (%)	8 (53.3)	0	7 (46.7)	0	3 (21.4)	0	19 (63.3)	2 (6.7)	15 (50.0)	0	12 (44.4)	0	16 (59.3)	1 (3.3)	19 (87.9)	0	16 (59.3)	0
Swelling (%)	11 (73.3)	11 (73.3)	7 (46.7)	7 (46.7)	10 (71.4)	9 (64.3)	12 (40.0)	11 (36.7)	22 (73.3)	22 (73.3)	16 (59.3)	16 (59.3)	28 (93.3)	27 (90.0)	22 (75.9)	22 (75.9)	23 (85.2)	23 (85.2)
Erythema (%)	0	0	0	0	0	0	0	0	0	0	0	0	0	0	0	0	1 (3.7)	1 (3.7)
General																		
Fever (%)	0	0	2 (13.3)	0	2 (13.3)	0	9 (30.0)	0	7 (23.3)	0	5 (18.5)	0	8 (26.7)	0	6 (21.4)	0	6 (22.2)	0
Drowsiness (%)	0	0	0	0	0	0	0	0	0	0	0	0	0	0	0	0	0	0
Irritability/Fussiness (%)	0	0	0	0	0	0	0	0	0	0	0	0	0	0	0	0	0	0
Loss of appetite (%)	2 (13.3)	0	2 (13.3)	0	0	0	0	0	0	0	0	0	1 (3.3)	0	0	0	0	0
Vomiting (%)	1 (6.7)	0	0	0	1 (7.1)	0	0	0	0	0	0	0	0	0	0	0	0	0

**Table 4:** Signs and Solicited Symptoms during the 7-Day Follow-Up Periods after Each Immunization: All Dose Regimens of Malaria Vaccine and Rabies Vaccine.

	FMP2.1/AS02_A_						Rabies vaccine					
	Immunization 1		Immunization 2		Immunization 3		Immunization 1		Immunization 2		Immunization 3	
	n = 75		n = 73		n = 68		n = 25		n = 25		n = 23	
	Overall	Severe	Overall	Severe	Overall	Severe	Overall	Severe	Overall	Severe	Overall	Severe
	n (%)	n (%)	n (%)	n (%)	n (%)	n (%)	n (%)	n (%)	n (%)	n (%)	n (%)	n (%)
Local												
Pain (%)	43 (57.3)	3 (4.0)	41 (56.2)	0	31 (45.6)	0	2 (8.0)	0	4 (16.0)	0	1 (4.3)	0
Swelling (%)	51 (68.0)	49 (65.3)	51 (69.9)	51 (69.9)	49 (72.1)	48 (70.6)	6 (24.0)	5 (20.0)	4 (16.0)	4 (16.0)	3 (13.0)	3 (13.0)
Erythema (%)	0	0	0	0	1 (1.5)	1 (1.5)	0	0	0	0	0	0
General												
Fever (%)	17 (22.7)	0	15 (20.5)	0	13 (19.1)	0	0	0	2 (8.0)	0	4 (17.4)	0
Drowsiness (%)	0	0	0	0	0	0	0	0	0	0	0	0
Irritability/Fussiness (%)	0	0	0	0	0	0	0	0	0	0	0	0
Loss of appetite (%)	3 (4.0)	0	2 (2.7)	0	0	0	0	0	0	0	0	0
Vomiting (%)	1 (1.3)	0	0	0	0	0	0	0	0	0	0	0

Overall, the lowest proportion of children (80%) having at least one local adverse event was observed in the 10 µg group among the younger children aged 1–2 years. All local solicited adverse events resolved without sequelae during the 7-day post-immunization periods.

#### Systemic solicited adverse events

Fever was the most common systemic adverse event observed and was more frequent in malaria vaccine recipients (Tables 3 and 4). The highest proportion of children with fever was observed in the 25 µg group, in which 30% of children had fever of mild to moderate intensity after immunization 1. The proportion of children reporting at least one systemic adverse event was the lowest among those aged 1–2 years and 5–6 years. Children in the 10 µg malaria vaccine and the rabies vaccine groups experienced the fewest systemic adverse events. All systemic solicited symptoms were of Grade 1 or Grade 2 intensity and all resolved during the 7-day follow-up period.

#### Unsolicited adverse events

Overall, 1,131 unsolicited adverse events were reported during the 30-day post immunization period. Unsolicited adverse events were balanced by study groups and were representative of local patterns of childhood illnesses. The majority of unsolicited symptoms for all age groups were acute respiratory tract infections, followed by malaria episodes and gastrointestinal disorders. Three unsolicited adverse events were graded as severe; all were instances of abnormal laboratory values in children aged 1–2 years old. A white blood cell (WBC) count of 17.7×10^3^/µL was detected on the day of the first immunization (prior to immunization) with the 10 µg dose of the malaria vaccine, and a WBC of 22.6×10^3^/µL was detected in another child 1 week after the first rabies vaccine immunization. Both of these elevated WBC counts were associated with concurrent minor infections. In the third case, a serum ALT of 364 U/L that was detected 1 week after the third immunization with the 50 µg dose of the malaria vaccine was determined to be due to hepatitis A infection, confirmed by serology. All abnormal lab values resolved within 1 month.

#### Serious adverse events

Four serious adverse events (SAE) occurred during the study. The first was a WBC elevation to 30.3×10^3^/µL (defined according to the protocol as an SAE) that was detected 1 week after the second immunization with the 10 µg malaria vaccine. This leukocytosis occurred contemporaneously with a malaria illness episode and resolved shortly after the end of the malaria episode. The second SAE was an elevation of serum ALT to grade 4 toxicity level at 521 U/L that was detected 1 week after receipt of the second dose of rabies vaccine. The high value was detected at a scheduled clinic visit and was not associated with any concerning clinical symptoms. Serological testing identified hepatitis A infection as the cause of the elevated ALT, which returned to normal after 2 weeks. Nevertheless the third immunization was withheld. The third and fourth SAEs were both ALT elevations that occurred in children with minor clinical symptoms and were determined by serological testing to be caused by a hepatitis B infection. In the first case, a moderate ALT elevation to 53 U/L was reported after the third immunization with the 25 µg dose of malaria vaccine. Thirty days later the ALT level was 1,260 U/L, constituting grade 4 toxicity and an SAE. Given the temporal relationship with vaccination an association cannot be ruled out, in that even with hepatitis B infection as the primary cause of ALT elevation, vaccination could have amplified the rise of ALT. The second case of hepatitis B occurred with a rise in ALT to 1,371 U/L on study day 90, a month after all 3 rabies vaccine immunizations had been completed, and was deemed not related to study products. All 4 SAEs resolved within 3 to 4 weeks of follow up with no sequelae.

#### Laboratory safety tests

Grade 1 elevated platelet count was the most common laboratory abnormality and was observed in 8, 16, 12 and 15 children in the 10 µg, 25 µg 50 µg and rabies vaccine groups, respectively. The highest platelet counts were reported in a 10 µg malaria vaccine recipient, with values of 1,096×10^3^/µL on the day of the second immunization (prior to immunization) and 1,397×10^3^/µL a week after the second immunization. These values were observed 5 weeks apart, with a normal platelet count in between. This child experienced several concurrent illnesses, including the SAE with very high WBC, and was not given the third vaccine dose due to moderate anemia.

Hemoglobin levels remained within or slightly above the normal range (9.8 g/dL to 12.4) for all but 4 participants throughout the study. Four children had grade 1 low hemoglobin levels measured at limited time points, one in the 10 µg group a week after the second immunization; and 3 in the 25 µg group occurring on the days of the first and second immunizations and a month after the third immunization, respectively. One grade 2 low hemoglobin was recorded in the 50 µg group on the day of the second immunization. Laboratory abnormalities detected on days of immunization were seen in blood samples that had been collected just prior to immunization.

Other abnormal hematology (lymphocyte count) and biochemistry (serum creatinine and ALT) laboratory abnormalities were evenly distributed among study groups. In addition to the 3 ALT abnormalities constituting SAEs described above (1 in the 25 µg group and 2 in the rabies vaccine group), 9 additional elevated ALT values were reported; 2 in the 10 µg group, 2 in the 25 µg group (1 attributed to hepatitis B); 4 in the 50 µg group (2 attributed to hepatitis A) and 1 in the rabies vaccine group (attributed to hepatitis A). No temporal pattern relative to immunizations was apparent in these cases of ALT elevation.

### Immunogenicity

**Figure 3:**
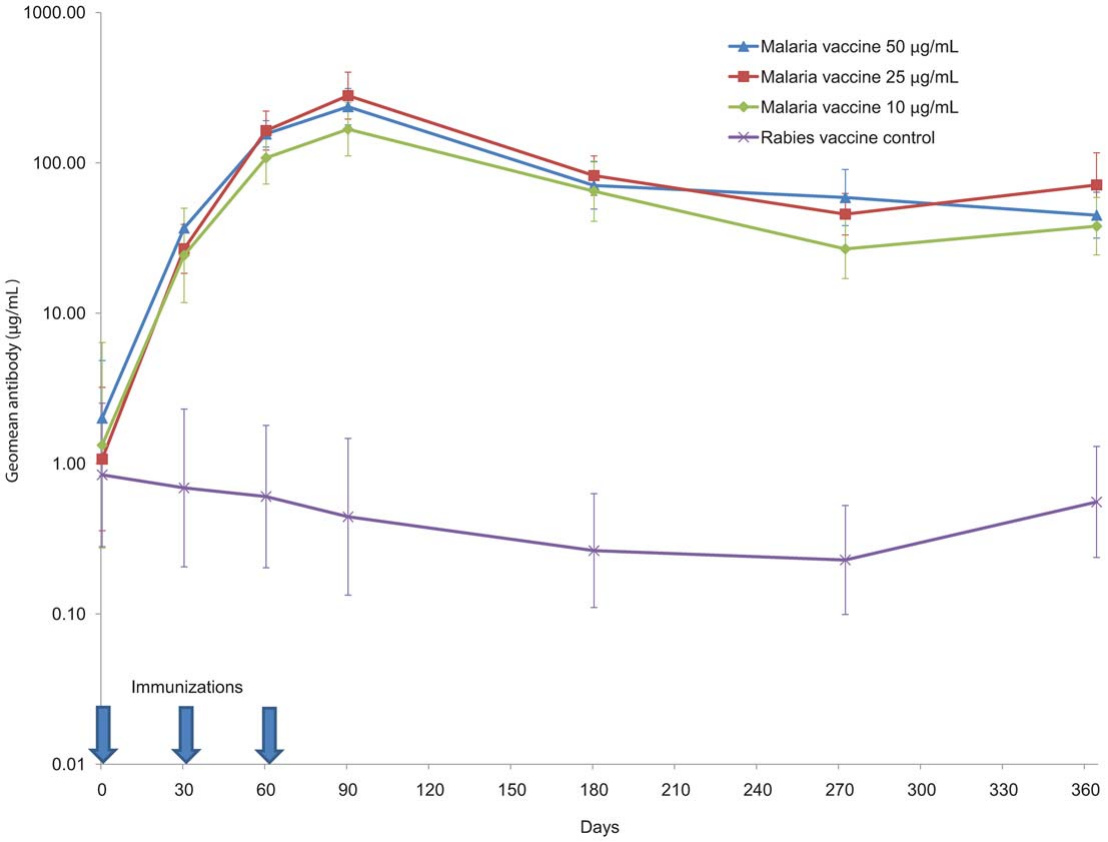
Anti-AMA1 antibody levels. Geometric mean of antibodies (µg/mL) to homologous recombinant AMA1 for FMP2.1/AS02_A_ 50 µg dose, FMP2.1/AS02_A_ 25 µg dose, FMP2.1/AS02_A_ 10 µg dose and rabies vaccine recipients. Times of each of 3 immunizations are indicated by arrows. Error bars represent 95 percent confidence intervals.

Baseline antibody levels were low in all groups (Figure 3). Immunization with the 3 dose levels was followed by a dramatic rise in anti-AMA1 antibodies, significantly higher than in the control group after a single immunization. Antibody levels peaked 4 weeks after the third immunization on study day 90 with 100-fold or greater rise relative to baseline, and remained significantly higher than in the control group throughout the 12 months of the study. In all groups the antibodies waned as the malaria transmission season was ending and rose as the new malaria season began. At all post-immunization time points, mean log antibody levels (µg/mL) were significantly higher in the malaria vaccine groups compared to the rabies vaccine group (t-test p-values <0.0001 at all post-immunization time points for each malaria vaccine dose group compared to rabies vaccine group). Longitudinal models showed the same results, with p-values<0.0001 at all post-immunization time points for each malaria vaccine dose group compared to rabies vaccine. All doses of the malaria vaccine induced similar high levels of antibodies, with overlapping 95% confidence intervals among the 3 malaria vaccine dose groups at all time points. Pre-immunization anti-AMA antibody levels tended to be lower in children in the youngest age group (1–2 years) than in older children (data not shown).

## Discussion

### Interpretation

This study is the first evaluation of the AMA1-based malaria vaccine FMP2.1/AS02_A_ in malaria-experienced children. The 3 dose levels of the malaria vaccine had acceptable tolerability. Local reactions were more frequent in malaria vaccine groups than in the comparator group. Pain and/or swelling at the injection site were experienced by most recipients of the malaria vaccine. Although swelling was often classified as grade 3 based on the size of the reaction (>20 mm), these episodes of swelling were short-lived and were usually unnoticed by participants (Figure 2). Nine participants received fewer than 3 vaccine doses. Four SAEs were observed; all were instances of grade 4 laboratory abnormalities. A high follow-up rate (96%) was achieved.

All 3 dose levels of the malaria vaccine elicited high levels of antibodies recognizing the vaccine antigen after a single immunization, peaking a month after the third immunization. The children in this trial had almost 30-fold lower baseline levels of anti-AMA1 antibodies than adults had in a previous trial of this vaccine at this site [17], and this lower baseline likely accounts for the more than 100-fold increase following immunization, compared to a 5- to 6-fold rise in the adult trial. These high levels were sustained for 1 year after the first dose was given. No significant differences in antibody levels were observed between the malaria vaccine groups.

The peak antibody levels reached a month after the third immunization in these children were similar to, or even slightly higher than, those seen previously in adults at this site. In adults we observed a tendency toward a dose response, with the 50 µg group having the highest antibody levels, while in children the 25 µg antibody levels were slightly higher. None of these differences, however, were statistically significant, and the proportions of children with 4-fold or higher rises in antibody levels was similarly high in all age groups and at all post-immunization time points (data not shown), supporting the conclusion that there was no dose effect. Since data on the duration of these responses were not available at the time the Phase 2 trial began, the highest dose with an acceptable safety profile, 50 µg, was selected for further evaluation.

### Generalizability

The safety and tolerability profile of the FMP2.1/AS02_A_ vaccine was similar to that seen in previous trials of this vaccine in North American malaria-naïve volunteers [16] and in Malian adults [17], as well as in trials of a similar recombinant protein blood-stage malaria vaccine with the same adjuvant in this and other African populations [29], [30]. As in these other trials, most of the adverse reactions both to the malaria vaccine and to the rabies vaccine were local and transient. There was no evidence of an increased risk of anemia, a concern raised in the report of the Phase 2 trial of the AMA1-C1 vaccine [20]

All 3 dose levels of the FMP2.1/AS02_A_ vaccine resulted in at least 100-fold increases in AMA1 antibody levels. While it is not possible to compare antibody levels measured in this trial with those seen in trials of other AMA1 vaccines, based on the relative fold-rise and duration of antibody responses this profile compares favorably with much lower and shorter-lived increases in antibody levels observed in Malian children immunized with AMA1-C1, an AMA1 vaccine adjuvanted with aluminum hydroxide [19], which provided no measurable protection in a recently reported Phase 2 trial at another site in Mali. Notably, while baseline antibody levels among children in this trial were about 10-fold to 50-fold lower than pre-immunization levels in adults at this same site, children achieved post-immunization AMA1 antibody levels that were at least as high as those seen in vaccinated adults [17], [20]. Moreover, the post-immunization AMA1 antibody levels in children were about 10-fold higher than baseline levels in semi-immune adults who have robust naturally acquired immunity that protects them against clinical malaria disease despite frequent infection.

Although the strong and sustained antibody responses observed in this trial are encouraging, it is not known whether antibodies raised against AMA1 based on the 3D7 clone of *P. falciparum* will protect against infection with the highly diverse forms of AMA1 found in nature [9], [13], [15]. The AMA1 vaccine that recently failed to demonstrate protection, AMA1-C1, is a bivalent vaccine based on genetically diverse AMA1 sequences derived from two different *P. falciparum* isolates. It is not yet known whether this lack of efficacy is due to an insufficiently robust immune response, to failure of allele-specific antibodies to protect against the wide array of AMA1 variants, or because immune responses to AMA1 alone are simply unable to prevent clinical malaria. High levels of inhibitory AMA1 antibodies were correlated with protection in a recent trial of AMA1 vaccines in Aotus monkeys [31], supporting the idea that AMA1-C1 could have failed because it was insufficiently immunogenic.

Previous studies have shown that the FMP2.1/AS02_A_ vaccine elicits antibodies that inhibit both parasite growth and AMA1 processing in homologous parasites [16] as well as measurable cellular immune responses [22]. Post-immunization sera from Malian adults who received the 50 µg dose of FMP2.1/AS02_A_ (but not sera from those who received the 25 µg dose) of the malaria vaccine had significantly greater growth inhibition activity against both 3D7 and FVO parasites than did post-immunization sera from the rabies comparator group in the previous Phase 1 trial [17]. However, until an AMA1 malaria vaccine demonstrates clinical efficacy against genetically diverse natural parasites, the relevance of growth inhibition assays and other humoral and cellular immunogenicity endpoints for clinical development decisions will remain a matter of reasoned conjecture.

### Overall Evidence

Based on its good safety profile, acceptable tolerability, and very robust antibody responses, the 50 µg dose of the AMA1-based malaria vaccine FMP2.1/AS02_A_ was selected for evaluation in a Phase 2 efficacy trial in children aged 1–6 years at the Bandiagara Malaria Project in Mali. If the results of this trial are promising, the development pathway for this vaccine could include incorporating the FMP2.1 antigen as one component of a multi-stage, multi-antigen malaria vaccine in combination with RTS,S [8], improved adjuvant formulations [32] and/or separate development as a disease-blocking vaccine for use in targeted populations in malaria transmission areas. As AMA1 malaria vaccines are evaluated in efficacy trials, the impact of genetic diversity of parasite antigens on vaccine efficacy is likely to emerge as a critical problem requiring integration of methods and concepts drawn from molecular epidemiology, molecular evolution, immunology and structural vaccinology [9], [13].

## Supporting Information

Protocol S1 Trial protocol
(0.39 MB PDF)

Checklist S1 Completed CONSORT checklist
(0.19 MB DOC)
