# Supplementary material for: Safety and Immunogenicity of an AMA1 Malaria Vaccine in Malian Children: Results of a Phase 1 Randomized Controlled Trial
Source: PLoS One. 2010 Feb 4;5(2):e9041. doi: 10.1371/journal.pone.0009041 (PMC2816207; doi:10.1371/journal.pone.0009041)
Supplement: Protocol S1 — Trial protocol (0.39 MB PDF) [file pone.0009041.s001.pdf]

RANDOMIZED, CONTROLLED, DOSE ESCALATION PHASE 1 CLINICAL TRIAL TO EVALUATE THE SAFETY AND IMMUNOGENICITY OF WRAIR'S AMA-1 MALARIA VACCINE (FMP2.1) ADJUVANTED IN GSKBIO'S AS02A VS. RABIES VACCINE IN 1-6 YEAR OLD CHILDREN IN BANDIAGARA, MALI

DMID Protocol Number: 05-0146

MMVDU Protocol Number: MMVDU-005

UMB IRB Protocol Number: H-28155

HSRRB Protocol Number: A-13920

IND Number: 11140

GSK Biologicals Protocol: Malaria 051 (106874)

DMID Funding Mechanism: NIAID U19AI065683

Pharmaceutical Support Provided by: GlaxoSmithKline Biologicals

IND Sponsor: Office of the Surgeon General, US Army

Principal Investigator: Mahamadou A. Thera MD MPH

DMID Protocol Champion: Abdollah Naficy MD

DMID Medical Monitor: Mirjana Nesin MD

DMID Clinical Affairs Specialist: Josephine Vinson MS

DMID Regulatory Affairs Specialist: Blossom Smith

Draft or Version Number: 2.0

30 January 2007

STATEMENT OF COMPLIANCE

The study described in this protocol will be conducted according to current Good Clinical Practices (US 21 CFR Part 50-Protection of Human Subjects and Part 56-Institutional Review Boards, US 45 CFR 46, 21 CFR 312, 32 CFR 219, 10 USC 980 and the applicable rules and regulations of Mali).

The University of Bamako Faculty of Medicine, Pharmacy and Odonto-stomatology IRB (FWA00001769), the University of Maryland IRB (FWA00007145), the U.S. Army Medical Research and Materiel Command's Human Subjects Research Review Board (HSRRB, FWA00000015) and the Division of Microbiology and Infectious Diseases (DMID), NIAID, NIH will review and approve the protocol prior to study start. Documentation of the approval by these bodies will be kept in the PI's study file and Sponsor's regulatory files.

SIGNATURE PAGE

The signatures below constitute the approval of this protocol and the attachments, and provide the necessary assurances that this trial will be conducted according to all stipulations of the protocol, including all statements regarding confidentiality, and according to local legal and regulatory requirements and applicable US federal regulations and ICH guidelines.

Research Center Director:

Signed: ______________________________ Date: ______________
Ogobara K Doumbo MD PhD
Professor

Site Principal Investigator:

Signed: ______________________________ Date: ______________
Mahamadou A. Thera MD MPH
Associate Professor

Co-Principal Investigator:

Signed: ______________________________ Date: ______________
Christopher V. Plowe MD MPH
Professor

TABLE OF CONTENTS

Page
Statement of Compliance.......2
Signature Page.......3
Table of Contents.......4
Protocol Summary.......10
1 Key Roles.......12
2 Background Information and Scientific Rationale.......19
2.1 Background Information.......19
2.1.1 Malaria parasite life cycle.......19
2.2 Disease burden.......19
2.2.1 Malaria in Mali.......19
2.3 Rationale.......20
2.3.1 The AMA-1 antigen.......20
2.3.2 Genetic diversity in AMA-1.......21
2.4 The AMA-1 FMP2.1/AS02A vaccine.......21
2.4.1 Preclinical toxicity, safety and reactogenicity of AMA-1 FMP2.1/AS02A.......22
2.4.2 Clinical experience with AMA-1 FMP2.1/AS02A.......22
2.4.3 Phase 1 trial in US adults.......23
2.4.4 Phase 1 trial in Malian adults.......27
2.4.5 Clinical Experience with the AS02A Adjuvant.......33
2.4.6 Summary of clinical experience with AMA-1 FMP2.1/AS02A.......34
2.4.7 Justification of 0-, 1-, 2-Month Schedule.......35
2.4.8 Justification of the vaccine dosing regimen and next steps for the vaccine.......35
2.4.9 Comparator Vaccine.......35
2.4.10 Safety of RabAvert® rabies vaccine.......36
2.5 Potential Risks and Benefits.......37
2.5.1 Potential Risks.......37
2.5.2 Vaccination.......37
2.5.3 Known Potential Benefits.......37
2.5.4 Risks to study personnel.......38
3 Objectives.......39
3.1 Study Objectives.......39
3.1.1 Overall study objective.......39
3.1.2 Primary specific objective.......39
3.1.3 Secondary specific objectives.......39
3.2 Study Outcome Measures.......39
3.2.1 Primary Outcome Measures.......39
3.2.2 Secondary Outcome Measures.......39
4 Study Design.......40
4.1 Overview.......40
5 Study Enrollment and Withdrawal.......42

5.1 Site description 42
5.2 Subject Inclusion Criteria 43
5.3 Subject Exclusion Criteria 43
5.3.1 Rationale for using clinical assessment of immunosuppression 45
5.4 Treatment Assignment Procedures 45
5.4.1 Randomization Procedures 45
5.4.2 Masking Procedures 46
5.4.3 Reasons for Withdrawal 47
5.4.4 Handling of Withdrawals 47
5.4.5 Termination of Study 47
6 Study Intervention/Investigational Product 49
6.1 Study Product Description 49
6.1.1 Acquisition 49
6.1.2 Formulation and Packaging 49
6.1.3 Product Storage and Stability 50
6.2 Dosage, Preparation and Administration of Investigational Product 51
6.2.1 Administration of vaccines 51
6.3 Accountability Procedures for the Investigational Product 51
6.4 Concomitant Medications/Treatments 51
7 Study Schedule 53
7.1 Screening 53
7.2 Enrollment/Baseline 54
7.3 Vaccination process 54
7.3.1 Dose escalation 54
7.4 Follow-up 54
8 Study Procedures/Evaluations 56
8.1 Standard operating procedures (SOPs) 56
8.2 Daily study procedures 57
8.2.1 Outline of study procedures 61
8.2.2 Health care provision 62
8.3 Final Study Visit 63
8.4 Early Termination Visit 63
8.5 Unscheduled Visit 63
9 Sample handling and analysis 64
9.1 Overview of sample collection, handling, transport and shipping 64
9.2 Overview of collection time points 64
9.2.1 Safety 64
9.2.2 Serology 64
9.2.3 CMI 64
9.3 Laboratory Assays 65
9.3.1 Hematology and biochemistry 65
9.3.2 Serology 65
9.3.3 Malaria smears 65
9.3.4 Additional assays 66

9.3.5 CMI and antibody avidity assays ... 66
9.3.6 Additional serology ... 67
9.3.7 AMA-1 sequencing and genotyping ... 67
10 Assessment of Safety ... 69
10.1 Specification of Safety Parameters ... 69
10.2 Methods and Timing for Assessing, Recording, and Analyzing Safety Parameters ... 69
10.2.1 Adverse Events ... 69
10.2.2 Surveillance period for adverse events ... 70
10.2.3 Recording adverse events ... 70
10.2.4 Solicited adverse events ... 70
10.2.5 Unsolicited adverse events ... 71
10.3 Assessment of intensity of non-serious adverse events ... 71
10.4 Assessment of causality ... 73
10.5 Following-up of adverse events and assessment of outcome ... 74
10.6 Clinical malaria ... 74
10.7 Serious adverse events ... 74
10.7.1 Definition of a serious adverse event ... 74
10.7.2 Reporting serious adverse events ... 75
10.8 Treatment of adverse events ... 78
10.9 Halting Rules ... 78
10.10 Safety Oversight ... 78
10.10.1 Local Medical Monitor ... 78
10.11 Data Safety and Monitoring Board (DSMB) ... 79
11 Clinical Monitoring ... 82
11.1 Site Monitoring Plan ... 82
12 Statistical Considerations ... 83
12.1 Primary endpoints ... 83
12.2 Secondary endpoints ... 83
12.3 Study cohorts/datasets to be evaluated ... 83
12.4 Sample Size Considerations ... 84
12.5 Final Analysis Plan ... 85
12.5.1 Analysis of demographics ... 85
12.5.2 Analysis of immunogenicity ... 85
12.5.3 Analysis of safety ... 86
12.5.4 Analysis of CMI responses ... 86
12.5.5 Analysis of AMA-1 sequences ... 86
13 Source Documents and Access to Source Data/Documents ... 88
14 Quality Control and Quality Assurance ... 89
15 Ethics/Protection of Human Subjects ... 90
15.1 Ethical Standard ... 90
15.2 Institutional Review Board ... 90
15.3 Informed Consent Process ... 91
15.3.1 Screening and study informed consent ... 91

15.3.2 Screening recruitment radio announcement text 93
15.3.3 Compensation 93
15.4 Subject Confidentiality 94
15.5 Study Discontinuation 94
15.6 Future Use of Stored Specimens 95
15.7 Justification for conducting the study in children 95
15.8 Justification for exclusion 95
16 Data Handling and Record Keeping 96
16.1 Data Management Responsibilities 96
16.2 Data Capture Methods 96
16.3 Types of Data 97
16.4 Study Records Retention 97
16.5 Protocol Deviations 97
17 Publication Policy 98
18 Literature 99
19 SupplementS/Appendices 104

List of Abbreviations

3D7:	A laboratory clone of *Plasmodium falciparum*
AE:	Adverse event
ALT:	Alanine aminotransferase
AMA-1:	Apical Membrane Antigen-1 of *P. falciparum*
AST:	Aspartate aminotransferase
AS02A:	Adjuvant system 2 of GlaxoSmithKline Biologicals without thimerosal
BMP:	Bandiagara Malaria Project (clinical trial site)
CBER:	Center for Biologics Evaluation and Research
CBC:	Complete blood count
CFR:	Code of Federal Regulations
CMI:	Cell mediated immunity
CRF:	Case report form
CS gene:	Circumsporozoite gene of *P. falciparum*
CSP:	Circumsporozoite protein
CTL:	Cytotoxic T lymphocyte
CVD:	Center for Vaccine Development, University of Maryland Baltimore
DMID:	Division of Microbiology & Infectious Diseases, NIAID, NIH
DNA:	Deoxyribonucleic acid
DSMB:	Data and Safety Monitoring Board
EGF:	Epidermal growth factor
FDA:	US Food and Drug Administration
FWA:	Federal-Wide Assurance
ELISA:	Enzyme linked immunosorbent assay
FMP1:	Falciparum Malaria Protein 1 (3D7 MSP1-42 vaccine candidate antigen)
FMP2.1:	Falciparum Malaria Protein 2.1 (3D7 AMA-1 vaccine candidate antigen)
FMPOS:	Faculty of Medicine, Pharmacy and Odonto-stomatology, University of Bamako, Mali
GCP:	Good clinical practice
GIA:	Growth inhibition assay
GSKBio:	GlaxoSmithKline Biologicals
GMT:	Geometric mean titer
HDCV:	Human Diploid Cell Vaccine (Rabies)
HSRRB:	U.S. Army Medical Research and Materiel Command's Human Subjects Research Review Board (Army IRB)
ICH:	International Conference on Harmonisation
ID:	Identification
IEC:	Institutional Ethical Committee
IM:	Intramuscular
IND:	Investigational new drug
IRB:	Institutional Review Board (ethical review committee), for this study including the University of Bamako Faculty of Medicine IRB, the University of Maryland Baltimore IRB, and the U.S. Army's Human Subjects Research Review Board
LMM	Local Medical Monitor

MPL:	Monophosphoryl lipid A
3D-MPL	3-deacylated monophosphoryl lipid A
MRTC:	Malaria Research and Training Center
MMVDU:	Mali Malaria Vaccine Development Unit
MSP-1:	Merozoite surface protein-1 of *P. falciparum*
MSP-2:	Merozoite surface protein-2 of *P. falciparum*
MVDB:	Malaria Vaccine Development Branch, NIAID, NIH
NIAID:	National Institute of Allergy and Infectious Diseases
NIH:	US National Institutes of Health
NANP:	Repeat epitopes of the circumsporozoite protein
PBMC:	Peripheral blood mononuclear cells
PI:	Principal Investigator
OCRA:	Office of Clinical Research Affairs, DMID, NIAID, NIH
QA:	Quality assurance
QC:	Quality control
QS21:	*Quillaja saponaria* 21 (saponin derivative)
RTS,S:	Fusion protein between circumsporozoite protein based antigen and Hepatitis B surface antigen and 226 amino acid polypeptide corresponding to the surface antigen of hepatitis B virus (adw serotype)
SBAS2:	SmithKline Beecham Adjuvant System 2
SAE:	Serious adverse event
SME:	Sponsor’s Medical Expert
SMC:	Safety Monitoring Committee
SOP:	Standard Operating Procedure
TRAP:	Thrombospondin adhesion protein
UMB:	University of Maryland Baltimore
USAID:	US Agency for International Development
USAMMDA:	US Army Medical Materiel Development Activity
WHO:	World Health Organization
WRAIR:	Walter Reed Army Institute of Research

PROTOCOL SUMMARY

Title: Randomized, controlled, dose escalation Phase 1 clinical trial to evaluate the safety and immunogenicity of WRAIR's AMA-1 malaria vaccine (FMP2.1) adjuvanted in GSKBio's AS02A vs. rabies vaccine in 1-6 year old children in Bandiagara, Mali

Phase: 1

Population: 100 healthy children aged 1-6 years residing in Bandiagara, Mali

Number of Sites: 1

Study Duration: 15 months

Subject Participation Duration: 14 months (including screening)

Description of Agent or Intervention:

- Recombinant subunit protein FMP2.1 (*Plasmodium falciparum* Apical Membrane Antigen-1 from strain 3D7 expressed in and purified from E*scherichia coli*), adjuvanted with:
- GSK's AS02A (proprietary oil-in-water emulsion and phosphate buffered saline with the immunostimulants monophosphoral lipid A and QS21)

Objectives:

Primary:

- Evaluate the safety and reactogenicity of FMP2.1/AS02A in children naturally exposed to *P. falciparum* malaria infection

Secondary:

- To measure the magnitude and duration of antibody response to FMP2.1 by ELISA

Description of Study Design: Randomized, controlled dose-escalation Phase 1 trial of the FMP2.1/AS02A malaria vaccine, using rabies vaccine as a control. Children aged 1-6 years will be randomized to three groups. Twenty subjects will be enrolled in cohort 1 and 40 subjects each in cohorts 2 and 3. Children within each cohort will be randomized in a 3:1 ratio to receive 10, 25 or 50 μg of FMP2.1 (in cohorts 1, 2 and 3, respectively) adjuvanted with a proportionate volume of the AS02A, or rabies vaccine. Thus a total of 75 children will receive the malaria vaccine and 25 the rabies vaccine. Immunizations will

be given on days 0, 30 and 60 in a staggered fashion, with the first administrations of the 25 and 50 μg dose levels of FMP2.1 following the first administration of the 10 and 25 μg dose levels, respectively, by 2-3 weeks. Solicited adverse events will be recorded on the days of immunization and days 1, 2, 3 and 7 after each immunization, and unsolicited adverse events will be recorded for 30 days after each immunization. Children will be followed for one year after the last immunization. Sera will be collected for anti-FMP2.1 antibody titers on the days of immunization and 14 days after each immunization as well as three, six, nine and twelve months after the first immunization.

Estimated Time to Complete Enrollment: 45 days

Schematic of Study Design:

Cohort 1	ARM 1	N=15	FMP2.1/AS02A 10 μg dose
	ARM 2	N=5	Rabies vaccine

↓

Interim blinded safety evaluation by DSMB after 1st 10 μg dose

↓

Cohort 2	ARM 1	N=30	FMP2.1/AS02A 25 μg dose
	ARM 2	N=10	Rabies vaccine

↓

Interim blinded safety evaluation by DSMB after 1st 25 μg dose

↓

Cohort 3	ARM 1	N=30	FMP2.1/AS02A 50 μg dose
	ARM 2	N=10	Rabies vaccine

Timeline for safety reviews by DSMB. Arrows down indicate immunization safety data included in safety review. Arrows up indicate review results in recommendation to proceed with immunization.

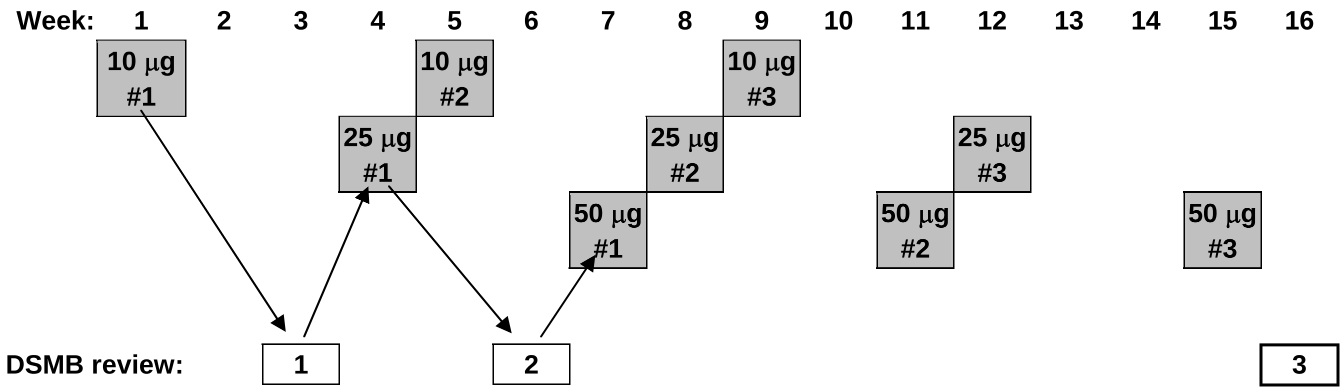

1 KEY ROLES

For questions regarding this protocol, contact:

Christopher V. Plowe MD MPH, Professor
Center for Vaccine Development
University of Maryland School of Medicine
685 West Baltimore Street, HSF 480
Baltimore, Maryland 21201 USA
Tel 410-706-3082, Fax 410-706-6205
E-mail: cplowe@medicine.umaryland.edu

Individuals:

DMID Protocol Champion:

Abdi Naficy MD, MPH, Medical Officer
Parasitology & International Programs Branch
Division of Microbiology & Infectious Diseases
National Institute of Allergy & Infectious Diseases
National Institutes of Health
6610 Rockledge Drive, MSC 6603
Bethesda, MD 20892, USA
Tel: (301) 435 2866, Fax: (301) 402 0659
E-mail: anaficy@niaid.nih.gov

IND Sponsor:

COL Jerome Pierson, Sponsor's Representative
Commander, U.S. Army Medical Materiel Development Activity
1430 Veterans Drive
Fort Detrick, MD 21702
Tel: (301) 619-7643
E-mail: Jerome.pierson@us.army.mil

Investigator Roles and Responsibilities:

[1]Supervision of clinical trial conduct
[2]Patient enrollment, follow-up and care
[3]Study design and planning, protocol development, data analysis
[4]Clinical laboratory testing
[5]Specimen collection and transport
[6]Primary immunogenicity assays
[7]Additional analyses
[8]Vaccine handling and preparation
[9]Vaccine administration
[10]Severe adverse event reporting

Principal Investigator:
Mahamadou A. Thera MD, MPH, Associate Professor[1,2,3,7,10]
Malaria Research and Training Center
Department of Epidemiology of Parasitic Diseases
Faculty of Medicine, Pharmacy and Dentistry
University of Bamako
BP 1805, Point G
Bamako, Mali, West Africa
Tel/Fax 223-222-8109
E-mail: mthera@mrtcbko.org

Co-Principal Investigator:
Christopher V. Plowe MD MPH, Professor[1,3,7,9,10]
Center for Vaccine Development
University of Maryland School of Medicine
685 West Baltimore Street, HSF 480
Baltimore, Maryland 21201 USA
Tel 410-706-3082, Fax 410-706-6205
E-mail: cplowe@medicine.umaryland.edu

Clinical Investigators:
University of Bamako
Ogobara K. Doumbo MD, PhD, Professor and Director[1,2,3,7]
Dapa A. Diallo MD, Professor[1,2,3,7]
Drissa Coulibaly MD, Research Associate[1,2,3,7]
Abdoulaye Koné MD, Research Associate[2,3,5,7]
Ando Guindo MD, Research Associate[2,3,5,7]
Karim Traoré MD, Research Associate[2,3,5,7]
Sory Diawara MD, Research Associate[2,3,5,7]
Malaria Research and Training Center
Department of Epidemiology of Parasitic Diseases
Faculty of Medicine, Pharmacy and Dentistry
University of Bamako
BP 1805, Point G
Bamako, Mali, West Africa
Tel/Fax: + 223-222 81 09; 221 63 61; 222 49 87

University of Maryland
Kirsten E. Lyke MD, Assistant Professor[1,3,5,7,9]
Matthew B. Laurens MD MPH, Fellow[2,3,7]
Center for Vaccine Development
University of Maryland School of Medicine
685 West Baltimore Street, HSF 480
Baltimore, Maryland 21201 USA

Tel 410-706-5328, Fax 410-706-6205

WRAIR
(WRAIR clinical investigators may be on site during parts of the trial in an advisory capacity but will not be directly responsible for clinical aspects of the trial)
D. Gray Heppner MD[3,7]
James F. Cummings MD, Sponsor's medical expert[3]
Michelle D. Spring MD[3] [Contractor]
Department of Immunology
503 Robert Grant Avenue
Silver Spring, MD 20910
Contact: D. Gray Heppner MD
Tel 301-319-9414, Fax 301-319-7358

Laboratory Investigators:

University of Bamako
Mounirou Baby PharmD, Associate Professor, Clinical Lab Manager[3,4,5,7]
Amadou Niangaly PharmD, Research Associate[3,4,5,7]
Mady Cissoko, PharmD, Research Associate[3,4,5,7]
Amagana Dolo PharmD, PhD, Professor, Study Pharmacist[3,8]
Modibo Daou, PharmD, Research Associate, Assistant Pharmacist[3,8]
Bourema Kouriba PharmD, PhD, Associate Professor[3,5,7]
Issa Diarra Pharm.D, Research Associate[3,4,7]
Malaria Research and Training Center
Department of Epidemiology of Parasitic Diseases
Faculty of Medicine, Pharmacy and Dentistry
University of Bamako
BP 1805, Point G
Bamako, Mali, West Africa
Tel/Fax: + 223-222 81 09; 221 63 61; 222 49 87

University of Maryland
Marcelo B. Sztein MD, Professor, for cellular immunology assays[3,7]
Shannon L. Takala PhD, for molecular assays[7]
Center for Vaccine Development
University of Maryland School of Medicine
685 West Baltimore Street, HSF 480
Baltimore, Maryland 21201 USA
Tel 410-706-5328, Fax 410-706-6205

WRAIR
V. Ann Stewart DVM PhD, for primary immunogenicity assays[3,6,7]
David Lanar PhD, for additional laboratory assays[3,7]
Sheetij Dutta PhD, for additional laboratory assays[3,7]

Department of Immunology
503 Robert Grant Avenue
Silver Spring, MD 20910
Contact: D. Gray Heppner MD
Tel 301-319-9414, Fax 301-319-7358

Co-Sponsors:
USAID
(USAID provides financial support for the trial)
Carter Diggs MD PhD[3]
Tel 202-712-5728
Lorraine Soisson PhD[3]
Tel 908-369-7704
USAID Malaria Vaccine Development Program

GSKBio
(GSKBio provides the adjuvant for the trial)
Amanda Leach MSc MRCPCH[3], Clinical Development Manager
Ally Olotu MD[3]
GSK Biologicals
89 Rue de l'Institut
1330, Rixensart, Belgium
Tel: +32-2-656-7788, Fax: +32-2-656-6160

Statistical Consultant:
Don Stablein, PhD
(NIH contractor will provide data management system and statistical support)
The EMMES Corporation
401 N. Washington St., Suite 700
Rockville, MD 20850
Tel: 301-315-6145, Fax: 301-251-1355
Email: dstablein@emmes.com

Clinical Trial Coordinator:
Drissa Coulibaly MD
Malaria Research and Training Center
Department of Epidemiology of Parasitic Diseases
Faculty of Medicine, Pharmacy and Dentistry
University of Bamako
BP 1805, Point G
Bamako, Mali, West Africa
Tel/Fax: + 223-222 81 09; 221 63 61

Data Quality Manager:

Issaka Sagara MD MSPH
Malaria Research and Training Center
Department of Epidemiology of Parasitic Diseases
Faculty of Medicine, Pharmacy and Dentistry
University of Bamako
BP 1805, Point G
Bamako, Mali, West Africa
Tel/Fax: + 223-222 81 09; 221 63 61; 222 49 87

Regulatory Affairs Specialists:
(Coordination of communications with sponsors and IRBs)
Karen Ball RN MS
Center for Vaccine Development
University of Maryland
685 West Baltimore Avenue
Baltimore MD 21201
Phone: 410-706-0431, Fax: 410-706-4171
E-mail: kball@medicine.umaryland.edu

Richard Potter (communications with FDA)
Division of Regulatory Affairs
U. S. Army Medical Materiel Development Activity
1430 Veterans Drive
Fort Detrick, MD 21702
Phone : 301-619-0317, Fax 301-619-0197
E-mail : richard.potter6@us.army.mil

Lisa Ware
Department of Immunology
503 Robert Grant Avenue
Silver Spring, MD 20910
Tel: (301) 319-9941, Fax: (301) 319-7358
E-mail: lisa.ware@us.army.mil

WRAIR Project manager:

(coordination of protocols and communications with study partners)
Tracey O. Smith
Department of Immunology
503 Robert Grant Avenue
Silver Spring, MD 20910
Tel: (301) 319-3121, Fax: (301) 319-7358
E-mail: tracey.o.smith@amedd.army.mil

USAMMDA Medical Monitor:
Lieutenant Colonel Arthur G. Lyons
WRAIR
Division of CD&I, Room 3A04
Silver Spring, MD 20910
Tel: (301) 319-9021
E-mail: Arthur.lyons@amedd.army.mil

DMID Medical Monitor:
Mirjana Nesin MD
Office of Clinical Research Affairs (OCRA)
Division of Microbiology & Infectious Diseases
National Institute of Allergy & Infectious Diseases
National Institutes of Health
6610 Rockledge Drive
Bethesda, MD 20892, USA
Tel: (301) 496 7067
E-mail: nesinm@niaid.nih.gov

Local Medical Monitor
Mariam Traore MD
Chief, Pediatric Service
Mopti Regional Hospital
Mopti, Mali
Tel: 223 601 0130

Institutions: University of Bamako
Malaria Research and Training Center
Department of Epidemiology of Parasitic Diseases
Faculty of Medicine, Pharmacy and Dentistry
BP 1805, Point G
Bamako, Mali, West Africa
Contact: Mahamadou A. Thera MD MPH
Tel/Fax 223-222-8109
E-mail: mthera@mrtcbko.org

University of Maryland School of Medicine
Center for Vaccine Development
685 West Baltimore Street, HSF 480
Baltimore, Maryland 21201 USA
Contact: Christopher V. Plowe MD MPH
Tel 410-706-3082, Fax 410-706-6205
E-mail: cplowe@medicine.umaryland.edu

Walter Reed Army Institute of Research

Department of Immunology
503 Robert Grant Avenue
Silver Spring, MD 20910
Contact: D. Gray Heppner MD
Tel 301-319-9414, Fax 301-319-7358
E-mail: donald.heppner@na.amedd.army.mil

The EMMES Corporation (NIH contractor for data and statistical support)
401 N. Washington St., Suite 700
Rockville, MD 20850
Tel: 301-315-6145, Fax: 301-251-1355
Email: mwolff@emmes.com

Office of the Surgeon General, US Army
U.S. Army Medical Materiel Development Activity
1430 Veterans Drive
Fort Detrick, MD 21702
Contact: Colonel Jerome Pierson
Tel: (301) 619-7643
E-mail: Jerome.pierson@us.army.mil

GlaxoSmithKline Biologicals
89 Rue de l'Institut
Rixensart 1330, Belgium
Contact: Amanda Leach MSc MRCPCH
Tel: +32-2-656-7788, Fax: +32-2-656-6160
E-mail: amanda.leach@gskbio.com

USAID Malaria Vaccine Program
Contact : Carter Diggs MD PhD
Tel 202-712-5728
E-mail: cdiggs@usaid.gov

2 BACKGROUND INFORMATION AND SCIENTIFIC RATIONALE

2.1 Background Information

2.1.1 Malaria parasite life cycle

Among the four species of Plasmodium that cause human malaria, *P. falciparum* is responsible for most disease and death. Its life cycle is complex. Disease occurs as a result of the asexual blood stage when parasites invade and grow inside red blood cells. Anopheline mosquitoes inject sporozoites which travel to the liver and invade hepatocytes. About 6-10 days later, each infected hepatocyte releases thousands of merozoites into the bloodstream. Within the red blood cells, *P. falciparum* merozoites develop into schizonts over 48 hours and produce 15-30 new merozoites, each able to invade other red cells. The blood stages of the infection produce clinical symptoms and malaria disease.

Particular to *P. falciparum* is its ability to modify the surface of the red blood cell in a way that the infected cells can adhere to the vascular endothelium and other tissues, where they cause disease. Parasite sequestration in various organs (brain, heart, liver, kidney, placenta) contributes to the pathogenesis of malarial disease. A small proportion of asexual parasites convert to sexual forms that transmit the infection to others through female Anopheline mosquitoes.

2.2 Disease burden

Africa bears the heaviest burden of malaria. The most dangerous parasite species, *P. falciparum*, is responsible for more than one million deaths worldwide each year. More than 90% of these deaths occurs among sub-Saharan African children under five years old. In areas of stable malaria transmission, 25% of all-cause mortality in children aged four years or less has been directly attributed to malaria (1). Evidence from impregnated bed net trials in West Africa indicates that malaria could account directly and indirectly for as much as 60% of all-cause mortality in children aged less than five years old (2-4).

2.2.1 Malaria in Mali

In Mali, where childhood mortality exceeds 20%, malaria is a leading cause of morbidity in the general population and of mortality in children aged less than ten years. The study site, Bandiagara (pop. 13,364), is 700 km northeast of Bamako on the Dogon plateau. Here the malaria transmission season is from July through December, with peak transmission in September and peak disease incidence in October. The predominant ethnic group is Dogon (80%) but many ethnic groups are represented in the community. About 85% of Bandiagara children aged 0-10 years have at least

one clinical episode of uncomplicated malaria during the malaria season, and the average number of clinical episodes of malaria per child and per transmission season was 1.92, with a few children experiencing a maximum of four clinical episodes (5).The incidence of severe malaria among children aged six years or less in Bandiagara was 2.5% (n=2284) in 2000. (6).

2.3 Rationale

A safe and effective malaria vaccine would be a major contribution to existing control tools. Two critical steps of the malaria life cycle have been closely examined as potential targets for vaccine-induced immunity: the invasion of liver cells by sporozoites and the invasion of red blood cells by merozoites.

The circumsporozoite protein (CSP), which coats the surface of the sporozoite, is critical for the invasion of liver cells and contains a peptide sequence that binds to the surface of liver cells (7). *P. falciparum* CSP is composed of a central repeating sequence (NANP) flanked by two non-repeat sequences. GlaxoSmithKline Biologicals (GSKBio) and the Walter Reed Army Institute of Research (WRAIR) developed a recombinant molecule that contains important T helper epitopes from CSP as well as the $(NANP)_{19}$ repeat fused to hepatitis B (8). A recent Phase 2b trial in more than 2000 children aged 1-4 years in Mozambique reported 30% efficacy against incidence of first clinical episodes of *P. falciparum* malaria and efficacy against severe malaria of 58% (9). The duration of protection against both clinical episodes and severe malaria was reported to have lasted at least 18 months in this trial (10).

The merozoite stage of the parasite is another logical target for a malaria vaccine since blockade of erythrocyte invasion would prevent clinical disease, leading to hope that a merozoite protein-based vaccine would prevent severe malaria and death (11). Several antigens have been identified that are involved in merozoite invasion of red cells. One of the most studied of these antigens and a promising blood stage vaccine candidate is the merozoite surface protein 1 (MSP1). A recombinant version of the 42-kDa C-terminal portion of MSP1, FMP1 (Falciparum Malaria Protein 1) has been produced at WRAIR in *E. coli*. The antigen is derived from the 3D7 clone of *P. falciparum* and contains both T-cell and B-cell epitopes. The vaccine is formulated in the AS02A adjuvant system. The FMP1/AS02A vaccine was evaluated in healthy malaria-exposed adults in Bandiagara in 2003-2004 and is currently being evaluated in an efficacy trial in 400 children in Western Kenya.

2.3.1 The AMA-1 antigen

Another promising antigen for blood-stage vaccine development is the apical membrane antigen 1 (AMA-1), a surface protein expressed during the asexual blood stage of *P. falciparum*. AMA-1 is produced as an 83-kDa polypeptide by mature schizonts in infected erythrocytes (12), and localizes in the microneme, an apical secretory organelle of the merozoite containing ligands for binding red cell receptors (13). The protein is processed to a 66-kDa protein that is subsequently exported to the merozoite surface where it plays a critical role in invading erythrocytes (14-17).

P. falciparum AMA-1 consists of a signal sequence, a large extracellular domain (ectodomain of 546 amino acids), a transmembrane domain (21 amino acids), and a C-terminal cytoplasmic domain (55 amino acids) (18). Comparisons between all of the known amino acid sequences of AMA-1 homologues indicate greater than 50% sequence identity, with 16 cysteine residues conserved in all sequences (19-21). All of the cysteines are found in the ectodomain of the molecule, which is stabilized by eight intramolecular disulfide bonds (22).

Seroepidemiologic studies conducted in West Africa and western Kenya demonstrate that natural antibody responses to AMA-1 are widespread and highly prevalent (23). Serological and cellular immune responses to AMA-1 increase with age in populations subject to continued exposure to malaria infection (24). Preliminary studies in Donéguébougou, Mali, demonstrate that natural antibodies exist in a large proportion of individuals (>90%) in this area of intense, seasonal transmission. In a cross-sectional study of 200 individuals aged 6 months to 45 years conducted in 2002 and 2003, median anti-AMA-1 antibody titers reached approximately 1000 at the peak of malaria transmission, although significant variation was seen between different age groups (25).

The naturally acquired antibodies to AMA-1 found in people living in malaria-endemic areas inhibit the growth of *P. falciparum in vitro* (26). This inhibition was dose-dependent and anti-AMA-1 antibodies recognized both strain-specific and conserved epitopes. From this evidence, it is reasonable to postulate that boosting the natural antibody response to AMA-1 through vaccination may protect an individual from illness due to the asexual blood stage of *P. falciparum* infection.

2.3.2 Genetic diversity in AMA-1

In vitro experiments and studies in both animals and humans have indicated some degree of allele-specificity in the antibody responses to genetically different forms of AMA-1 (26-30). We have sequenced domain I of AMA-1 in 357 *P. falciparum* infections from 100 individuals with clinical *P. falciparum* infections in Bandiagara in 1999, 2000, and 2001. A very high degree of polymorphism was observed within and between years, with a total of 101 unique haplotypes (distinct genetic sequences of AMA-1 domain I) observed among the 357 infections (31). The most common haplotype was identical to the 3D7 parasite strain of *P. falciparum* on which the FMP2.1 antigen is based. However, the 3D7 haplotype had a frequency of only about 10% each year; thus 90% of infections in Bandiagara were different from the vaccine strain with respect to one or more amino acids. The degree to which a 3D7-based vaccine will protect against non-3D7 type parasites in vivo is not known and this will be studied in the Phase 2 efficacy trial that will follow the current Phase 1 trial.

2.4 The AMA-1 FMP2.1/AS02A vaccine

FMP2.1 is a lyophilized preparation of the ectodomain of the 3D7 clone of *P. falciparum* AMA-1. FMP2.1 is comprised of 478 amino acids, 449 of which are derived from the merozoite surface protein AMA-1 of the malaria parasite, *P. falciparum*, 3D7 clone. The protein is produced in and purified from *E. coli* bacteria at the WRAIR BioProduction Facility. The gene encoding the FMP2.1 protein was chemically synthesized to contain an *E. coli*-optimized codon usage to encode the amino acids representative of amino acids 83 to 531 of the AMA-1 protein from the 3D7 clone of *P.*

falciparum. The purified antigen is adjuvanted with AS02A (proprietary oil in water emulsion and phosphate buffered saline + MPL + QS21). This candidate vaccine is intended to limit malaria morbidity and mortality, and possibly infection, by stimulating host immune responses against the AMA-1 of *P. falciparum*.

Following a Phase 1 study in malaria-naïve US volunteers, the first Phase 1 study in a malaria-exposed population of the AMA-1-based vaccine, FMP2.1/AS02A, was recently completed in adults in Bandiagara. As detailed below, the vaccine was safe and well tolerated and showed a dose-dependent immune response in adults in Bandiagara. The study proposed here is a Phase 1 safety and immunogenicity study of this vaccine in children. This will be the first pediatric study of the FMP2.1/AS02A candidate vaccine. If shown to be safe and immunogenic in children, a Phase 2 study of safety and efficacy in similarly-aged children will quickly follow at this same site using the dose selected in the present study. Demonstration of efficacy would then be followed by further development of an AMA-1 vaccine either alone or in combination with other malaria vaccine candidates, possibly including RTS,S, other genotypes of AMA-1, or other blood stage antigens, such as the MSP1-based vaccine recently tested in Mali and currently being tested in Kenya.

2.4.1 Preclinical toxicity, safety and reactogenicity of AMA-1 FMP2.1/AS02A

Clinical grade lots of FMP2.1 protein have been administered to mice, guinea pigs, rabbits, and rhesus monkeys. As summarized in the attached Investigators' Brochure, no safety concerns arose from preclinical studies.

2.4.2 Clinical experience with AMA-1 FMP2.1/AS02A

FMP2.1/AS02A has been administered to a total of 63 adult volunteers (Table 1; 182 doses total) in two dose-escalation Phase 1 protocols, identified as Malaria-033 and Malaria-037. In Malaria-033, the three dosage levels (approximately 10 µg, 25 µg or 50 µg FMP2.1 in 0.5 mL AS02A) were found to be safe and well-tolerated in healthy malaria-naïve adults in the U.S. In Malaria-037, two dosage levels (approximately 25 µg FMP2.1 in 0.25 mL AS02A and approximately 50 µg FMP2.1 in 0.5 mL AS02A) were tested and found to be safe and well-tolerated in healthy malaria-exposed adults in Mali.

Table 1: Doses of AMA-1 FMP2.1/AS02A administered in clinical studies

Study Designation	~50 µg FMP2.1 in 0.5 mL AS02A		~25 µg FMP2.1 in either 0.5 mL or 0.25 mL AS02A[1]		~10 µg FMP2.1 in 0.5 mL AS02A		Total	
	Total number of subjects	Total number of doses	Total number of subjects	Total number of doses	Total number of subjects	Total number of doses	Total number of subjects	Total number of doses
Malaria-033	7	19[2]	8	22[3]	8	22[4]	23	63
Malaria-037	20	59[5]	20	60	N/A	N/A	40	119

**TOTALS**	**27**	**78**	**28**	**82**	**8**	**22**	**63**	**182**

[1] In Malaria-033 all doses of antigen were administered with a 0.5 mL AS02A. In Malaria-037 the doses were prepared using a fixed ratio of antigen to adjuvant (~25 µg FMP2.1 formulated in 0.25 mL AS02A and ~50 µg FMP2.1 formulated in 0.5 mL AS02A)

[2] Five of the seven volunteers in this group received three immunizations. Two volunteers received only two immunizations.

[3] Seven of the eight volunteers in this group received three immunizations. One volunteer received only one immunization.

[4] Seven of the eight volunteers in this group received three immunizations. One volunteer received only one immunization.

[5] Nineteen volunteers in this group received three immunizations. One volunteer received only two immunizations.

Adverse events (AEs) and serious adverse events (SAEs) were defined in standard fashions as described in detail in the attached Investigator’s Brochure.

2.4.3 Phase 1 trial in US adults

Malaria-033: “Phase 1 dose escalation study to evaluate safety, reactogenicity, and immunogenicity of FMP2.1/AS02A in malaria-naïve adults in the U.S.” was an open-label Phase 1, dose-escalation study to evaluate the safety, reactogenicity, and immunogenicity of FMP2.1 reconstituted with AS02A adjuvant. This study was initiated in September 2003 at the WRAIR Clinical Trials Center. Twenty three participants were enrolled and assigned to receive approximately 10 µg (N=8), 25 µg (N=8), or 50 µg (N=7) doses of antigen with 0.5 mL AS02A on a 0-, 1-, 2- month schedule by intramuscular injection. As shown in Table 2, in the 10 µg and 25 µg dosage groups, 22 of the 24 scheduled doses were given; seven of the eight volunteers in each of these groups received all three immunizations with the eighth volunteer receiving only one immunization. In the 50 µg dosage group, 19 of the 21 scheduled doses were given; five of the seven volunteers in this group received all three immunizations with the other two volunteers receiving two immunizations. All three doses were found to be safe and well tolerated. FMP2.1/AS02A was immunogenic in all volunteers.

2.4.3.1 Safety/Reactogenicity data

AEs were assessed for intensity. Injection site pain was graded as 0 = absent, 1 = painful on touch, 2 = painful when limb is moved, and 3 = spontaneously painful. Solicited symptoms were graded as 0 = normal, 1= easily tolerated, 2 = interferes with normal activity, and 3 = prevents normal daily activity. Additional grading scales were applied to visible swelling at the injection site; 0 = none, 1= >0 to 20 mm, 2 = >20 to 50 mm, and 3 = > 50 mm, and to oral temperature; 0 = < 37.5 ^{o}C, 1 = 37.5 to 38 ^{o}C, 2 = >38 to 39 ^{o}C, and 3 = >39 ^{o}C.

All vaccine-related AEs occurred within 72 hours after vaccination (Table 2). Ten grade 3 reactions were reported with seven of ten occurring in the 50 µg dosage group and the remaining three occurring in the 25 µg dosage group (Table 2). Five of the Grade 3 reactions occurred in one individual in the 50 µg group after the second vaccination and were self-reported, as the individual did not return for follow-up during the time he was symptomatic. Local pain (43 incidents over 63 vaccinations), local swelling (8 incidents over 63 vaccinations) and headache (12 incidents over 63 vaccinations) accounted for most of the solicited AEs (Table 3).

There was one SAE reported during the study. The SAE was an episode of paroxysmal supraventricular tachycardia that occurred two days following the first immunization in a volunteer in the 25 µg dosage group. It was determined the volunteer had similar, unreported symptoms prior to entry into the study. She was evaluated by cardiology and underwent extensive testing including electrocardiogram, blood testing, echocardiogram, nuclear medicine study and cardiac MRI. There was no evidence of structural abnormality, myocarditis, or pericarditis. Additionally, the case was reviewed by the Director of Cardiac Electrophysiology at Walter Reed Army Medical Center. Cardiology concluded the event was consistent with paroxysmal supraventricular tachycardia that predated enrollment in the study and was not related to immunization. The volunteer was withdrawn from the study by the Principle Investigator. On follow up she was asymptomatic until seven months after vaccination when she experienced the same symptoms after placement of a purified protein derivative (PPD) for routine tuberculosis screening.

Table 2: Summary of Incidence of Adverse Events (Malaria-033)

Group	Grade 1		Grade 2		Grade 3	
	First 72 hours after vaccinations	Day 7	First 72 hours after vaccinations	Day 7	First 72 hours after vaccinations	Day 7
~10 µg FMP2.1 in 0.5 mL AS02A	11	0	6	0	0	0
~25 µg FMP2.1 in 0.5 mL AS02A	17	0	25	0	3	0
~50 µg FMP2.1 in 0.5 mL AS02A	23	0	14	0	7[1]	0
**Total**	**51**	**0**	**45**	**0**	**10**	**0**

[1] Five of the seven Grade 3 reactions in this group were self-reported by one individual after second vaccination.

Table 3 Solicited Adverse Event Totals (Malaria-033)

	~10 µg FMP2.1 in 0.5 mL AS02A			~25 µg FMP2.1 in 0.5 mL AS02A			~50 µg FMP2.1 in 0.5 mL AS02A		
	Grade1	Grade2	Grade3	Grade1	Grade2	Grade3	Grade1	Grade2	Grade3
Pain	6	4	0	6	13	1	6	7	0
Redness	0	0	0	0	0	1	2	0	0
Swelling	1	1	0	1	1	1	3	0	0
Fever	0	0	0	0	0	0	0	2	0
GI	0	0	0	2	0	0	0	0	0
HA	2	0	0	2	3	0	2	1	2
Malaise	0	0	0	1	2	0	1	0	2
Myalgia	2	1	0	3	3	0	2	1	1
Fatigue	0	0	0	2	2	0	2	3	1
Arthralgia	0	0	0	0	1	0	2	0	1
**Total**	**11**	**6**	**0**	**17**	**25**	**3**	**23**	**14**	**7**

2.4.3.2 Immunogenicity data

Humoral Immune Response

There was a robust three-log anti-AMA-1 antibody response to all three vaccine dosages as determined by ELISA (Table 4 and Figure 1). After the second immunization, the antibody response in all three dosage groups was similar with no statistical differences between groups by analysis of variance. Increases in antibody titer were most pronounced after the first and second immunizations, with only a marginal increase after third immunization. During the six months of follow-up after the third immunization, antibody titers decayed slightly less than one log. The antibody response was consistent in all individuals. The average coefficient of variation of log titer at all subsequent time points after the second injection was less than 10% for all vaccine groups.

Table 4 Anti-FMP2.1 Geometric Mean Antibody Titers by ELISA (Malaria-033 [Malaria-Naïve Adults])

Vaccine Group	Day 0[1]	Day 14	Day 28[1]	Day 42	Day 56[1]	Day 70	Day 112	Day 168	Day 224
~10 µg FMP2.1 in 0.5mL AS02A	15	55	290	52464	40591	93149	42792	18125	10212
~25 µg FMP2.1 in 0.5mL AS02A	34	411	1181	72424	48827	92243	49495	25732	14888
~50 µg FMP2.1 in 0.5mL AS02A	14	379	2827	73097	51216	116069	71707	31046	16754

[1] Immunizations were given on days 0, 28, and 56.

Figure 1: Anti-FMP2.1 Antibody Response by ELISA (log scale) (Malaria-033)

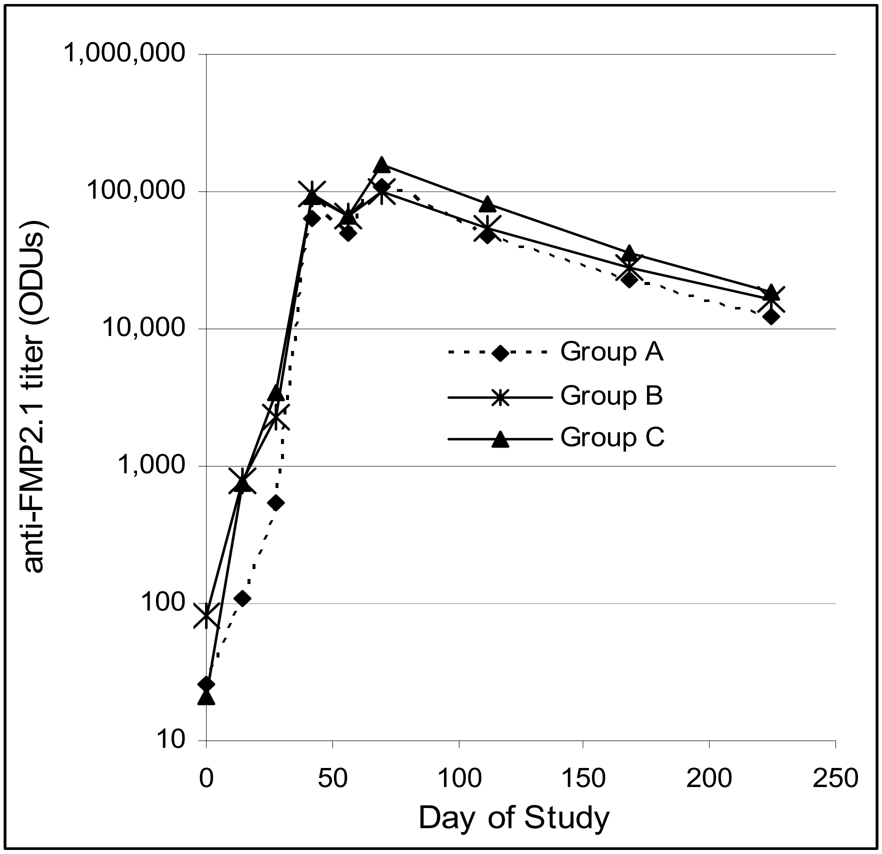

Group A: ~10µg FMP2.1/0.5 mL AS02A
Group B: ~25µg FMP2.1/0.5 mL AS02A
Group C: ~50 µg FMP2.1/0.5 mL AS02A

2.4.4 Phase 1 trial in Malian adults

Based on the safety and immunogenicity results of Malaria-033, a protocol entitled “Double-blind, randomized, controlled Phase 1 dose escalation trial to evaluate the safety and immunogenicity of the WRAIR AMA-1 malaria antigen (FMP2.1) formulated in GSKBio’s AS02A vs. rabies vaccine in malaria-experienced adults in Bandiagara, Mali” (Malaria-037) began in November 2004. Sixty adults were randomized in a 2:1 fashion in two staggered cohorts of 30 to receive by deltoid intramuscular injection approximately 50 μg FMP2.1 in 0.5 mL AS02A (n=20) or rabies vaccine (n=10) (cohort 1); or approximately 25 µg FMP2.1 in 0.25 mL AS02A (n=20) or rabies vaccine (n=10) (cohort 2). Thus a fixed antigen:adjuvant ratio was used in Malaria-037 as will be done for the present trial, in contrast to Malaria-033 which used a fixed volume of adjuvant. The immunizations were administered on study days 0, 30, and 60.

One hundred seventy-five adults were screened and 60 healthy adults aged 18-55 were enrolled and immunized. Solicited symptoms were actively monitored for eight days after immunization. Subjects were queried regarding local signs and symptoms including injection site pain, erythema, swelling and arm motion limitation; and general systemic signs and symptoms including headache, fever, chills, nausea, myalgia, joint pain and malaise. Unsolicited symptoms were monitored for 30 days after each immunization, and SAEs were monitored for one year after the first immunization. Antibody titers to FMP2.1 were measured by ELISA on sera collected on study day 0 and 14, and 30 days after each immunization, as well as 6, 9, and 12 months after the first immunization. All 60 participants received the first two immunizations and 59 participants received all three immunizations. One person in the FMP2.1 50 μg group had his third dose withheld due to a previous elevated ALT, described in the next section. As of study day 364, two participants have been lost to follow-up. Both of those participants were from the 25 µg group and received all three doses of FMP2.1. One of the participants left the study area on his study day 62 and could never be contacted again and the second participant was a female who moved and was unable to return for her study day 364 procedures.

This study found that both dosage levels of FMP2.1/AS02A were safe and well tolerated by malaria-experienced adults in Bandiagara, and that the vaccine produced a significant, dose-dependent antibody response.

2.4.4.1 Safety/Reactogenicity data

AEs were assessed for intensity. Grading scales used to determine the intensity of the following adverse events are described in Table 5.

- Grade 0 = No adverse event.
- Grade 1 = An adverse event that is easily tolerated by the subject, causing minimal discomfort and not interfering with everyday activities.
- Grade 2 = An adverse event that is sufficiently discomforting to interfere with normal everyday activities.

- Grade 3 = An adverse event that prevents normal, everyday activities. Such an adverse event would for example prevent attendance at work/school and would require the administration of corrective therapy.

Table 5: Assessment of adverse event intensity

Adverse Event	Intensity grade	Intensity definition
Pain at injection site	0	None
	1	Pain that is easily tolerated
	2	Pain that interferes with daily activity
	3	Pain that prevents daily activity
Swelling at injection site<br>Record size	0	0 mm
	1	>0 - ≤ 20 mm
	2	>20 - ≤ 50 mm
	3	>50 mm
Erythema at injection site<br>Record size	0	0 mm
	1	>0 - ≤ 20 mm
	2	>20 - ≤ 50 mm
	3	>50 mm
Limitation of arm motion - Abduction at the shoulder	0	None
	1	>90° but <120°
	2	>30° but ≤90°
	3	≤30°
Fever<br>Record oral temperature	0	<37.5ºC
	1	37.5 - ≤38.0ºC
	2	>38.0 - ≤39ºC
	3	>39ºC
Chills	0	None
	1	Chills that are easily tolerated
	2	Chills that interfere with daily activity
	3	Chills that prevent daily activity
Nausea	0	None
	1	Nausea that is easily tolerated
	2	Nausea that interferes with daily activity
	3	Nausea that prevents daily activity
Headache	0	None
	1	Headache that is easily tolerated
	2	Headache that interferes with daily activity
	3	Headache that prevents daily activity
Malaise	0	None
	1	Malaise that is easily tolerated
	2	Malaise that interferes with daily activity
	3	Malaise that prevents daily activity
Myalgia	0	None
	1	Myalgia that is easily tolerated
	2	Myalgia that interferes with daily activity
	3	Myalgia that prevents daily activity
Joint pain	0	None
	1	Joint pain that is easily tolerated
	2	Joint pain that interferes with daily activity
	3	Joint pain that prevents daily activity

Solicited Grade 3 symptoms, which consisted entirely of local injection site swelling of greater than 5 cm across at the widest dimension, were more common in the 50 μg group than in the 25 μg group or control group (Table 7). This swelling did not interfere with normal daily activities and was usually unnoticed by the participants and detected by careful physical examination. AEs were balanced by group and were typical of common medical problems in Bandiagara (Table 6). Laboratory toxicities were graded using the DMID guidelines (available at: http://www.niaid.nih.gov/dmid/clinresearch/). One alanine aminotransferase (ALT) elevation to 562 U/L was detected in an individual in the 50 μg FMP2.1/AS02A group who had negative serological testing for viral hepatitis. The participant had been using a non-steroidal anti-inflammatory drug that is associated with elevated liver transaminases, and after peaking on study day 37 the ALT was normal on study day 60. In the opinion of the investigators and the local medical monitor this was not considered to be related to vaccination, but a decision was made to withhold the third vaccination from this individual. There were mild, clinically insignificant abnormalities in complete blood count (CBC), creatinine and ALT that were balanced by group. No SAEs were reported through study day 364.

Table 6: Summary of Incidence of Adverse Events (Malaria-037)

Group	Grade 1	Grade 2	Grade 3
	First 7 days after vaccinations	First 7 days after vaccinations	First 7 days after vaccinations
~25 µg FMP2.1 in 0.25 mL AS02A	191	32	6
~50 µg FMP2.1 in 0.5 mL AS02A	191	26	21
Rabies vaccine (RabAvert®)	142	4	5
**Total**	**524**	**62**	**32**

Table 7: Solicited Adverse Event Totals (Malaria-037)

	~25 µg FMP2.1 in 0.25 mL AS02A			~50 µg FMP2.1 in 0.5 mL AS02A			Rabies vaccine (RabAvert®)		
	Grade1	Grade2	Grade3	Grade1	Grade2	Grade3	Grade1	Grade2	Grade3
Pain	44	1	0	49	4	0	22	0	0
Erythema	0	0	0	0	1	0	0	0	0
Swelling	7	24	6	3	12	21	3	3	5
Limitation of arm movement	4	1	0	3	0	0	0	0	0
Headache	8	0	0	15	3	0	10	0	0
Fever (Oral T >37.5^{o}C)	5	0	0	6	0	0	1	0	0
Malaise	4	0	0	4	1	0	3	0	0
Myalgia	5	0	0	6	1	0	3	0	0
Arthralgia	0	0	0	1	1	0	3	0	0
Chills	4	0	0	2	0	0	1	0	0
Nausea	5	0	0	1	0	0	1	0	0
**Total**	**86**	**26**	**6**	**90**	**23**	**21**	**47**	**3**	**5**

Figure 2: Incidence of Grade 3 solicited symptoms during the eight-day follow-up period after 3 immunization (Malaria-037)

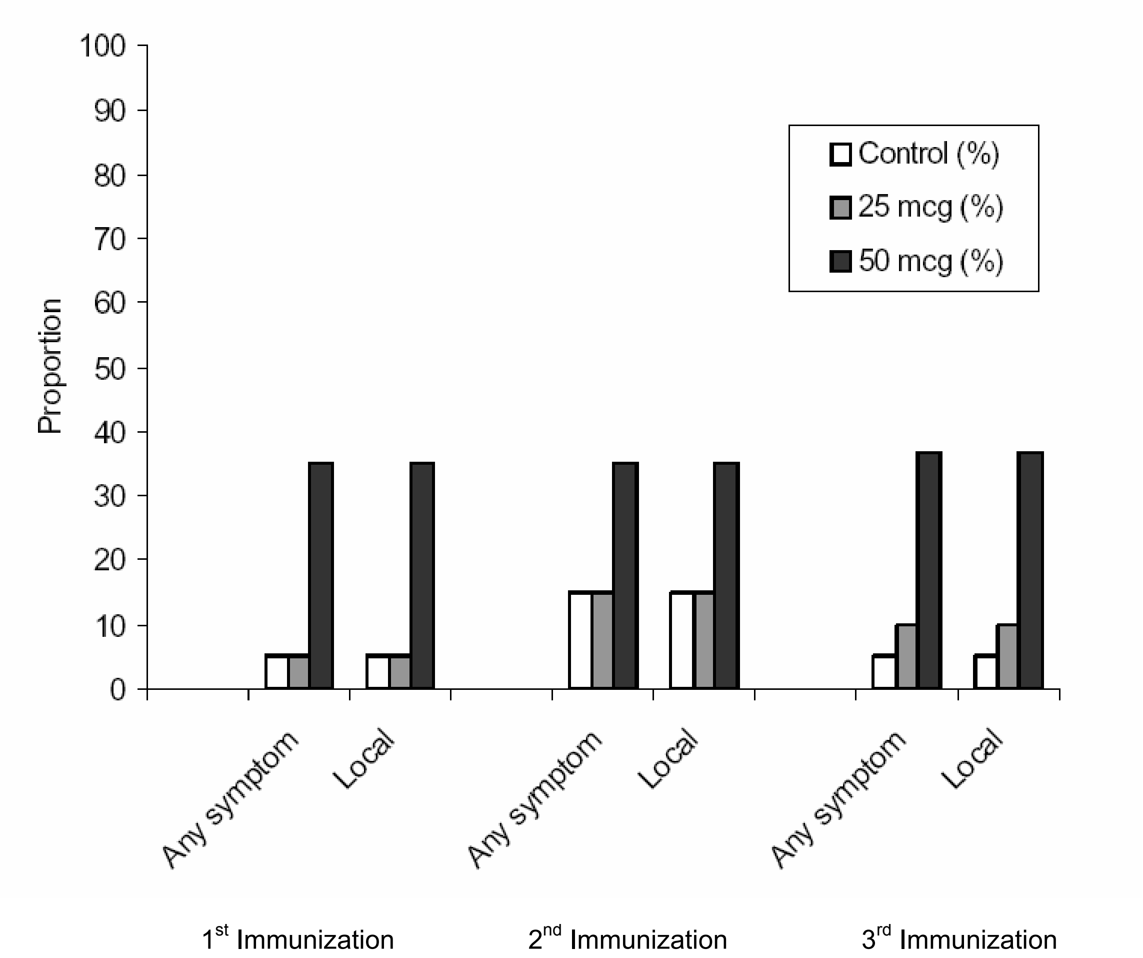

2.4.4.2 Immunogenicity Data

Humoral Immune Response: Volunteers receiving either the 25 or 50 µg dose of FMP2.1 developed anti-FMP2.1 antibody responses that were significantly higher than the rabies vaccinated volunteers. Two weeks after the third immunization there were 6- and 11- fold increases in anti-FMP2.1 titers respectively in the 25 and 50 µg dosage groups over the rabies control group. After the second immunization there was a significant difference between the geometric mean titers of the FMP2.1 vaccinated individuals and the rabies vaccinated individuals achieved significant difference ($p<0.05$ on log-transformed data). However, there was no significant difference between the 25 and 50 µg dosage groups after the second immunization but there is a trend towards significance ($p=0.07$) (Table 8). Similarly to the malaria-naïve volunteers immunized in Malaria-033, the coefficients of variance for the log transformed data from the malaria-exposed volunteers immunized in Malaria-037 were 10.1% or less after the second immunization whereas in the rabies

vaccinated individuals the average coefficient of variance was 20.9% and did not vary significantly over time (Figure 3). The baseline antibody titers were higher in this malaria-exposed population than in the malaria-naïve participants in Malaria-033, but the magnitude of the post-immunization responses was similar.

Table 8 Anti-FMP2.1 Geometric Mean Antibody Titers by ELISA (Malaria-037 [Malaria-Exposed Adults])

	Day 0[1]	Day 14	Day 30[1]	Day 44	Day 60[1]	Day 74	Day 90	Day 180
~25 µg FMP2.1 in 0.25 mL AS02A		30794	31805	55246	61439	89857	60967	50750
~50 µg FMP2.1 in 0.5 mL AS02A	23500	69752	55086	100590	119191	150738	102813	pending
Rabies vaccine (RabAvert®)	14355	13857	12691	13941	16030	14064	10425	7846

[1] Immunizations were given on days 0, 30, and 60.

Figure 3: Anti-FMP2.1 Antibody Response by ELISA (log scale) (Malaria-037)

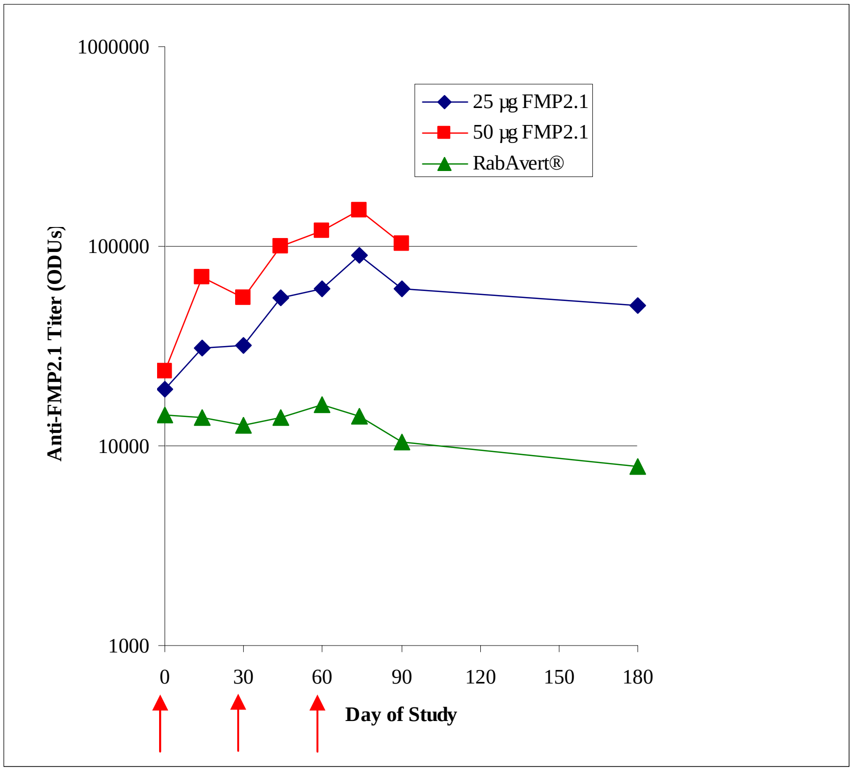

Note: Immunizations performed on day indicated by arrows. Assays for Day 180+ for the low-dose group and later dates for full-dose and control groups are in progress.

2.4.5 Clinical Experience with the AS02A Adjuvant

The adjuvant system AS02A, previously known as SBAS2, consists of an oil-in-water emulsion and phosphate buffered saline combined with two immunostimulants, monophosphoryl lipid A (MPL) and a saponin derivative known as Stimulon QS21 (a triterpene glycoside purified from the bark of *Quillaja saponaria)* (32-34). MPL is a detoxified, deacylated form of monophosphoryl lipid A, derived from the lipopolysaccharide (LPS) of *Salmonella minnesota*. LPS, and more specifically, its lipid A component, has long been known for its strong adjuvant effects; however, until recently, its high toxicity precluded its use in a vaccine formulation. Ribi et al. (35) showed that the monophosphorylated form of lipid A retains its adjuvant function and almost completely loses its endotoxin effects. Subsequently, the 3-deacylated form of MPL was shown to have a further decrease in its toxicity as tested in small animals, but retains its immunopotentiating effect (36). Several immunogenicity studies performed in mice, guinea pigs, monkeys, and humans have shown that inclusion of 3D-MPL into a vaccine preparation potentiates both specific antibody and cellular immune responses (36-38). The term MPL in this protocol refers to the 3-deacylated form of the compound. To date, the bulk of the experience with this formulation in malaria vaccines has been in conjunction with the RTS,S antigen reviewed above.

Table 9. Summary of clinical experience with the AS02A adjuvant in African subjects.

Vaccine	Location (reference if published)	Age (years)	Number of recipients of AS02A	Number of doses	Dose
RTS,S/AS02A	Gambia (39)	18-45	20	60	0.5mL[1]
RTS,S/AS02A	Gambia (40)	18-45	153	435	0.5mL[1]
RTS,S/AS02A	Gambia (41)	6-11<br>6-11<br>6-11	20<br>20<br>20	60<br>59<br>60	0.5mL[1]<br>0.25mL[2]<br>0.1mL[3]
RTS,S/AS02A	Gambia (41)	1-5<br>1-5<br>1-5	30<br>30<br>30	89<br>89<br>89	0.5mL[1]<br>0.25mL[2]<br>0.1mL[3]
RTS,S/AS02A	Mozambique	1-4	30	84	0.25mL[2]
RTS,S/AS02A	Mozambique (9;10)	1-4	803	2326	0.25mL[2]
RTS.S/AS02A	Kenya	18-45	20	60	0.5mL[1]
RTS,S/AS02A	Kenya	18-35	85	~240	0.5mL[1]
FMP1/AS02A	Kenya (42)	18-55	~20	~60	0.5mL[4]
FMP1/AS02A	Mali	18-55	20	60	0.5mL[4]
FMP1/AS02A	Kenya	1-4	90	N/A	0.5 mL[4]<br>0.25 mL[5]<br>0.10 mL[6]

FMP1/AS02A	Kenya	1-4	200	N/A	0.5 mL[4]
FMP2.1/AS02A	Mali	18-55	40	19<br>20	0.5 mL[7]<br>0.25 mL[8]

[1] 0.5mL = 50μg RTS,S + 0.5mL AS02A
[2] 0.25mL = 25μg RTS,S + 0.25mL AS02A
[3] 0.1mL = 10μg RTS,S + 0.1mL AS02A
[4] 0.5mL = 50μg FMP1 + 0.5mL AS02A
[5] 0.25mL = 25μg FMP1 + 0.25mL AS02A
[6] 0.10 mL = 10 μg FMP1 + 0.10 mL AS02A
[7] 0.5 mL = 50 μg FMP2.1 + 0.5 mL AS02A
[8] 0.25 mL = 25 μg FMP2.1 + 0.25 mL AS02A
FMP1 – MSP-1-based vaccine; FMP2.1 – AMA-1 based vaccine, RTS,S – CSP-based vaccine; N/A – Not available

These studies, with a large cumulative experience in both malaria-naïve and malaria-experienced adults and children, have demonstrated that AS02A has a good safety profile when used with the RTS,S vaccine, and suggest that it is similarly safe and well-tolerated when formulated with the FMP1 and FMP2.1 blood-stage vaccines.

2.4.6 Summary of clinical experience with AMA-1 FMP2.1/AS02A

On the basis of preclinical and clinical studies performed to date with FMP2.1/AS02A, there are no risks of particular severity or seriousness anticipated with the formulation.

Although the two previous clinical studies with FMP2.1/AS02A have resulted in grade 3 reactions; these reactions were generally short-lived and resolved in less than 48 hours after vaccination. In malaria-exposed Malian adults followed for a year, there were more unsolicited adverse events, as expected. Malian adults also experienced more local injection site swelling, which was short-lived and usually not symptomatic. This raises the possibility that the vaccine is more reactogenic in malaria-exposed than in malaria-naïve individuals and justifies the careful dose escalation proposed in this protocol.

Extensive clinical data exist for AS02A administered with FMP2.1, additional malarial antigens, hepatitis B surface antigen (HBsAg), and HIV antigens. Clinical development with these vaccines is ongoing. In general the AS02A formulated vaccines are safe and well tolerated when administered to adults, both naïve and malaria experienced, and in malaria experienced children. The most common side effect related to immunization is pain at the injection site. Redness and swelling are less frequent. The most frequent general side effects are headache and malaise, usually mild to moderate and short in duration. Various studies have shown that AS02A formulated vaccines are safe and well tolerated and the reactogenicity profile was clinically acceptable. Based on the experience with the FMP2.1 antigen and the AS02A adjuvant, other than standard precautions for idiosyncratic or allergic reactions, a need for special monitoring is not anticipated in the present trial.

2.4.7 Justification of 0-, 1-, 2-Month Schedule

The overall testing program of FMP2.1 aims at developing a vaccine for administration with routine childhood vaccines. With this goal in mind, the present study uses a 0-, 1-, and 2-month immunization schedule anticipating that this schedule will be more amenable to eventual incorporation into the Expanded Programme on Immunization of the WHO.

2.4.8 Justification of the vaccine dosing regimen and next steps for the vaccine

The clinical development of the candidate malaria vaccine FMP2.1/AS02A is progressing in four distinct steps; 1) a Phase 1 dose-escalation trial in malaria naïve adults (completed at WRAIR), 2) a Phase 1 dose-escalation trial in malaria-experienced adults (recently completed in Mali), 3) a Phase 1 dose-escalation study in young children that will result in dose selection for an efficacy trial (the present protocol), and 4) an efficacy trial of the selected dose in the target pediatric population (to proceed based on the outcome of the present study).

The overall goal of this Phase 1 study in malaria-experienced children is to choose a safe, well-tolerated and immunogenic dose to take forward into efficacy testing in children. In taking this product from adults to young children, it is prudent to perform a dose-escalation study starting with a fraction of the highest dose administered to adults. Therefore the safety of the 10 μg dose level will be assessed in a small cohort of 20 children (15 randomized to FMP2.1/AS02A and five to rabies vaccine) before any children receive the 25 or 50 μg dose levels. Because there was a significant dose-dependent difference in immunogenicity between the 25 and 50 μg dose levels in the adult Phase 1 trial of this vaccine in Mali, the possibility of a similar dose-dependent improvement in antibody response warrants testing both the 25 and 50 μg dose levels in children. Therefore we propose three dosage groups for this study: 10 µg FMP2.1 in 0.1 mL AS02A as the initial safety step, followed by 25 µg FMP2.1 in 0.25 mL AS02A, and then by 50 µg FMP2.1 in 0.5 mL AS02A to choose the optimal dose for further development based on safety, reactogenicity and immunogenicity.

2.4.9 Comparator Vaccine

2.4.9.1 Rationale for rabies comparator vaccine

Having a comparator vaccine is useful in Phase 1 trials conducted in malaria-endemic areas, since background immunity and natural exposure to malaria may make it difficult to interpret immunogenicity data. In this setting, rising titers of antibody to AMA-1 could be due to immunization or to natural exposure or both. The use of a control group will permit comparison of immune responses and may result in a clearer interpretation of serological results. While a placebo control group would accomplish this same end, using a vaccine that is beneficial to the subjects further increases the benefit to risk ratio, which is always relatively low in a Phase 1 trial. We have chosen to use rabies vaccine as the comparator for several reasons: 1) the dosing schedule is compatible with that of FMP2.1; 2) to be able to compare results directly with recent and ongoing studies of the FMP1/AS02A and FMP2.1/AS02A vaccines in Mali and Kenya that used rabies vaccine as a

comparator; and 3) the available evidence supports a benefit for participants who receive rabies vaccine. A placebo would offer no benefit.

Rabies prevalence in Mali is not known but available data from the Ministry of Health's Division of Epidemiology suggest that the rabies burden is high. Approximately 1,500 dog bites were reported to public health officials in Bamako, the capital of Mali, from 1996 through 1999. The vast majority of the dogs are unvaccinated and in most cases the status of rabies infection is unknown. The heads of 124 dogs were examined for evidence of rabies infection: rabies infection was found in 34 (27%); 7 cases were negative and there were no reported results for 74 cases (60%). These incomplete data allow us to estimate that 25-30% or more of dogs that bite humans may be carrying and potentially transmitting rabies infection. In the Bandiagara district health center, approximately one case of human rabies is reported per year. This is likely a considerable underestimate of the true incidence of rabies cases given the general population's reliance on traditional healers and the relatively low utilization of the district health center. Of note, three dog bites were reported among the 40 study subjects during a recent Phase 1 trial of malaria vaccine FMP1/AS02A in Kenya that used the rabies vaccine as a comparator. Because of the potential benefit to Bandiagara residents, participants in the two previous malaria vaccine trials who received malaria vaccine were offered rabies vaccine at the end of the trials.

When the RabAvert® rabies vaccine is administered according to the recommended immunization schedule (days 0, 7, 21), nearly 100% of subjects attain a protective titer. In two studies carried out in the US in 101 subjects, protective antibody titers >0.5 IU/mL were obtained by day 28 in all subjects. In studies carried out in Thailand in 22 subjects, and in Croatia in 25 subjects, antibody titers of >0.5 IU/mL were obtained by day 14 (injections on days 0, 7, 21) in all subjects (43-46).

High antibody titers have also been demonstrated with off-label immunization with rabies vaccines. Among participants in England, Germany, France and Belgium who received two immunizations one month apart, nearly 100% of the participants developed specific antibody and the geometric mean titer for the group was 10 IU (47-50). The proposed immunization schedule of 0, 1, and 2 months is therefore expected to be highly successful in conferring protective immunity against rabies among the control participants.

2.4.10 Safety of RabAvert® rabies vaccine

Local and/or mild systemic reactions may occur after injection of RabAvert® rabies vaccine but these are usually transient and do not contraindicate continuing immunization. Local reactions such as induration, swelling and reddening have been reported more often than systemic reactions. In a comparative trial in normal volunteers, the most commonly reported adverse reaction to RabAvert was pain at the injection site, reported in 34% of 19 volunteers who received RabAvert. Localized lymphadenopathy was reported in about 15%. The most common systemic reactions were malaise (15%), headache (10%), and dizziness (15%). In a recent study in the USA (4), 83 subjects received RabAvert. The most common adverse reaction was pain at the injection site in 84%. The most common systemic reactions were headache (52%), myalgia (53%) and malaise (20%). None of the adverse events was serious; almost all were of mild or moderate intensity.

Uncommonly observed adverse events include temperatures above 38°C (100°F), swollen lymph nodes, and gastrointestinal complaints. In rare cases, patients have experienced severe headache, fatigue, circulatory reactions, sweating, chills, monoarthritis and allergic reactions; transient paresthesias and one case of suspected urticaria pigmentosa have also been reported. Human serum albumin (HSA) is present in RabAvert at concentrations less than 0.3 mg/dose. No type III hypersensitivity reactions have been observed with RabAvert. Serious systemic anaphylactic reactions or neuroparalytic events have been reported in association with RabAvert administration. Against a background of 11.8 million doses distributed world-wide, ten cases of encephalitis (one death) or meningitis, seven cases of transient paralysis including two cases of Guillain-Barré Syndrome, one case of myelitis, one case of retrobulbar neuritis, and two cases of suspected multiple sclerosis have been temporally associated with the use of RabAvert. Also, two cases of anaphylactic shock have been reported.

2.5 Potential Risks and Benefits

2.5.1 Potential Risks

2.5.2 Vaccination

Risks associated with both FMP2.1/AS02A and rabies vaccinations include local inflammatory reactions to the injected product, such as injection site pain and swelling. Systemic effects generally associated with vaccines may include flu-like syndrome, fever, chills, nausea/GI symptoms, headache, malaise, myalgia and arthralgia. While rare, allergic reactions, including life-threatening anaphylaxis, are associated with many vaccine preparations and must therefore be considered as a potential risk in this study. Risks associated with drawing blood include fainting, infection and bruising.

Free medical treatment will be provided to enrolled participants during the active immunization phase and the surveillance period, at a level that meets the local Malian standards of medical diagnosis and treatment. Medical care for ailments not related to vaccination will not extend beyond the study period. Medical care for ailments related to vaccination will extend, at minimum, until the condition has resolved or stabilized.

2.5.3 Known Potential Benefits

Participants may not receive any direct benefit from the experimental vaccine. However, they will receive follow-up medical care at the BMP clinic. Routine and emergency health care will be provided by study clinicians at the Bandiagara District Hospital, in collaboration with District Hospital physicians. Free initial medical evaluations by study clinicians and basic treatments will also be provided to family members of participants and to other non-study participants who seek care at the research clinic, with appropriate referral to the District Hospital or other sources of care as appropriate. During the conduct of the study participants randomized to receive rabies immunization will benefit from this due to the reduced risk of rabies, which is present in this region. At the end of

the study all participants and their parents/guardians will be informed of the vaccine they received. Participants randomized to the FMP2.1/AS02A vaccine will be offered rabies immunization at that time. These immunizations will be given at the recommended schedule of 0, 7 and 21 days.

The benefits to the community include an overall improvement of the level of medical care provided at the District Hospital resulting from the ongoing presence of the research team, including high-quality malaria diagnosis and case management of severe malaria and other conditions. Because of heightened awareness in the community of severe malaria has resulted in more frequent and earlier treatment-seeking for severe malaria and lower case fatality rates. If this research is successful and a safe and effective malaria vaccine is developed and licensed, the community of Bandiagara and the rest of the malaria-endemic world will benefit.

2.5.4 Risks to study personnel

The main risks to study personnel are from accidental exposure to blood and body-fluid borne infections. SOPs for staff safety are used in clinical and laboratory areas, including sharps management, hazardous waste management, etc. Universal precautions are used for handling all body fluids.

3 OBJECTIVES

3.1 Study Objectives

3.1.1 Overall study objective

Identification of an optimal pediatric dose of FMP2.1/AS02A that is safe, with high immunogenicity and acceptable reactogenicity.

3.1.2 Primary specific objective

Evaluate the safety and reactogenicity of FMP2.1/AS02A in children naturally exposed to *P. falciparum* malaria infection.

3.1.3 Secondary specific objectives

Measure the magnitude and duration of antibody response to FMP2.1 by ELISA assay.

3.2 Study Outcome Measures

3.2.1 Primary Outcome Measures

1. Occurrence of solicited symptoms after each vaccination during a 7-day surveillance period (day of vaccination and days 1, 2, 3 and 7 after vaccination)
2. Occurrence of unsolicited symptoms after each vaccination during a 30-day surveillance period (day of vaccination and 30 subsequent days)
3. Occurrence of serious adverse events throughout the study period

3.2.2 Secondary Outcome Measures

1. Titers and activity of anti-FMP2.1 antibody at each time point where serology samples are analyzed, measured by ELISA.

4 STUDY DESIGN

4.1 Overview

- Randomized, controlled, dose-escalation phase I study
- One study center
- 100 healthy children aged 1-6 years residing in the rural town of Bandiagara, Mali
- Safety oversight from a DSMB and LMM
- Study monitoring by DMID
- Screening will be done within 35 days prior to the first immunization
- Dose escalating by 10 μg, 25 μg and 50 μg of FMP2.1 adjuvanted with 0.1 mL, 0.25 mL and 0.5 mL of AS02A, respectively
    - 20 children will be randomized to receive either the 10 μg dose of FMP2.1/AS02A (n=15) or rabies vaccine (n=5)
    - 40 children will be randomized to receive either the 25 μg dose of FMP2.1/AS02A (n=30) or rabies vaccine (n=10)
    - 40 children will be randomized to receive either the 50 μg dose of FMP2.1/AS02A (n=30) or rabies vaccine (n=10)
- Immunization schedule will be on study days 0, 30 +/- 7, and 60 +/- 7
- Each consecutive immunization with the 25 μg or 50 μg dose level of FMP2.1 will be administered at least 14 days after the corresponding immunization with the 10 or 25 μg dose of FMP2.1, respectively, to permit review of day 7 safety laboratory results.
- Route of immunization will be left deltoid muscle IM
- Source documents will be derived from the electronic CRFs. This will contain all the protocol required information including clinical laboratory test results and adverse event medical records. The information in the source document will then be entered directly into the Internet data system.
- Study duration will be approximately 14 months per participant

- 7-day surveillance after each immunization (day of vaccination and study days 1, 2, 3 and 7 for solicited adverse events

- 30-day surveillance after each immunization for unsolicited adverse events

- Follow-up of serious adverse events (SAEs) until resolution or stability

- Beginning Study Day 120, participants will be visited and assessed by local guides at home at monthly intervals and return to clinic every three months for safety surveillance.

- The primary unblinded analysis will be conducted for all primary and secondary endpoints at a data-lock-point one month post Immunization 3 (study day 90), after which the Primary Study Report will be produced.

- The study will then continue in a single-blind fashion. Data gathered during this period will be reported in an addendum report.

- At the end of the study participants and their parents/guardians will be informed which vaccine they received and those who received malaria vaccine will be offered immunization with the rabies vaccine.

5 STUDY ENROLLMENT AND WITHDRAWAL

5.1 Site description

Participants for this study will be drawn from the population of healthy children aged 1-6 years residing in the town of Bandiagara, which has been the site of MRTC malaria studies since 1993. Bandiagara (pop. 13,364), is a rural town 700 km northeast of Bamako, the capital of Mali. In a census conducted in 1999, the predominant ethnic group is Dogon (64%) followed by Peuhl (12%), Bambara (6%) and 16 other ethnic groups. About 85% of residents reported their religion to be Islam, and about 8% reported belonging to various Christian denominations. Animist beliefs and practices are also common. The most common occupations among men were in agriculture (30%) with 21% in the commercial sector, mostly self-employed as vendors of various goods, and the rest reported a wide variety of occupations including 6% who were government workers and smaller numbers of teachers, health workers, traditional healers and practitioners of spiritual and magical arts. Nearly 80% of women reported their occupation as housewife, but it is evident to the casual observer that a large proportion of the women in Bandiagara are also working in agriculture and/or as producers and sellers of various goods. Forty-six percent of adults had received no formal schooling, and only 14% reported themselves to be literate. However, 86% of children of the age to be in primary school (7-11 years) were attending school. Seventy-five percent of marriages were reported to be monogamous and 25% were polygamous with 2-4 wives.

The annual per capita income for Mali as a whole is estimated to be $600. However, this figure is based on large segments of the population who live in remote rural areas with virtually no cash economy, and per capita income in Bandiagara is higher than this. Income data specifically for Bandiagara are not available, but Bandiagara is a relatively affluent town by Malian standards, with a lively market economy and a significant tourist trade. Most homes are single story structures made of mud brick and/or concrete organized into compounds with several small dwellings surrounding a central courtyard. A main road runs through the length of the town, which extends about 2 km from end to end. All homes of potential participants in this trial are within walking distance of the BMP research clinic.

Since 1998 Bandiagara has been the site of an NIAID-supported contract for developing a site for testing malaria vaccines, known as the Bandiagara Malaria Project (BMP). Support for the BMP through the year 2010 is provided by a cooperative agreement from NIAID awarded to the Universities of Maryland and Bamako, with supplemental support from USAID, to conduct Phase 1 and 2 pediatric trials of the FMP2.1/AS02A malaria vaccine. The BMP has completed censuses of Bandiagara, established a laboratory with the capability of preserving sera, peripheral blood mononuclear cells (PBMCs), live parasites and DNA; and a clinical research center where malaria diagnosis and hemoglobin and blood glucose levels are routinely determined and where children with severe malaria are hospitalized and cared for. Thousands of children have been included in malaria research studies and treated for uncomplicated and severe malaria (5;6;51-53). Loss to follow-up has consistently been less than 7%.

The BMP research clinic is on the campus of the Bandiagara District Hospital, where two buildings were renovated in 2003 for the first of two NIAID-supported malaria vaccine clinical trials that have been conducted. The clinical research facility includes an air-conditioned clinical laboratory and vaccine storage preparation room, six private consultation rooms, a procedure room, covered waiting area, two vaccination rooms, a resuscitation suite, an observation room, and storage and administrative space. Locked cabinets with restricted access are used for data storage.

The BMP team has been managing severe malaria at this site since 1998, providing 24-hour inpatient care. In addition to the present capacity for 24-hour nursing care and physician coverage, and laboratory tests for hematological and biochemistry parameters, new and upgraded pediatric inpatient care facilities are being established for this trial including capacity for blood transfusion and oxygen supplementation. Children requiring X-rays will be transported to the District Hospital in Mopti, 60 km from the site on a paved road. Children requiring diagnostic or therapeutic interventions not available locally will be transported to the National Pediatric Hospital Gabriel Toure in Bamako.

The BMP team has been trained to conduct GCP-compliant studies, using source documents, written informed consent and standardized case report forms. The site has been monitored several times by DMID’s contractor PPD, Inc., the US Army, and GSKBio as well as by the World Health Organization. The BMP team has also established a strong trust and rapport with the community, which is very accepting of conducting malaria vaccine trials there.

The site is connected to the MRTC central laboratory in Bamako via a VSAT system, which allows high-speed communication link with Bamako, US partner institutions and the Internet. Back-up Internet service for E-mail is available locally.

5.2 Subject Inclusion Criteria

- Age 1-6 years inclusive at the time of screening
- Residing in Bandiagara town
- Appear to be in generally good health based on clinical and laboratory investigation
- Separate written informed consent obtained from the parent/guardian before screening and study start, respectively
- Available to participate in follow-up for the duration of study (14 months)

5.3 Subject Exclusion Criteria

- Previous vaccination with an investigational vaccine or a rabies vaccine

- Use of a investigational or non-registered drug or vaccine other than the study vaccine(s) within 30 days preceding the first study immunization, or planned use up to 30 days after the third immunization

- Chronic administration (defined as more than 14 days) of immunosuppressants or other immune-modifying drugs within six months prior to the first immunization. This includes any dose level of oral steroids or inhaled steroids, but not topical steroids

- Confirmed or suspected immunosuppressive or immunodeficient condition, including human immunodeficiency virus (HIV) infection

- Confirmed or suspected autoimmune disease

- History of allergic reactions or anaphylaxis to immunizations or to any vaccine component

- History of serious allergic reactions to any substance, requiring hospitalization or emergent medical care

- History of allergy to tetracycline, doxycycline, nickel or Imidazole

- History of splenectomy

- Laboratory evidence of liver disease (alanine aminotransferase [ALT] greater than the upper limit of normal of the testing laboratory = 49.6 U/L).

- Laboratory evidence of renal disease (serum creatinine greater than the upper limit of normal of the testing laboratory = 0.5 mg/dL (44.2 µmol/L), or more than trace protein or blood on urine dipstick testing).

- Laboratory evidence of hematologic disease (absolute leukocyte count <5,300/mm^3 or >15,300/mm^3, absolute lymphocyte count <2,300mm^3, platelet count <133,000/mm^3, or hemoglobin <9.0 g/dL).

- Chronic skin condition that could interfere with vaccine site reactogenicity assessment

- Administration of immunoglobulins and/or any blood products within the three months preceding the first study immunization or planned administration during the study period.

- Simultaneous participation in any other interventional clinical trial

- Acute or chronic pulmonary, cardiovascular, hepatic, renal or neurological condition, severe malnutrition, or any other clinical findings that in the opinion of the PI may increase the risk of participating in the study

- Other condition that in the opinion of the PI would jeopardize the safety or rights of a participant in the trial or would render the participant unable to comply with the protocol

5.3.1 Rationale for using clinical assessment of immunosuppression

We will not test for HIV routinely at the time of screening. HIV seroprevalence is 1.7% in Mali, one of the lowest rates in sub-Saharan Africa. Although no serosurveys have been done in Bandiagara, this site is in a remote rural area and almost certainly has a lower prevalence rate than the average for Mali. After working at this site for the past nine years, we have only very rarely encountered young adults, and virtually no young children, with illnesses that raised clinical suspicion of an immunosuppressive disease. In addition, the age distribution of HIV infection in Africa is bimodal, with most infection detected in young infants infected through maternal-to-child transmission or in sexually active teens and adults. Infection in children aged 1-6 years is almost certainly extremely rare in this setting. Therefore, testing for HIV would likely yield few, if any, cases of HIV in this small study.

This study is intended to produce comparable results to other Phase 1 trials of recombinant protein blood stage malaria vaccines adjuvanted with AS02A, including recent and ongoing trials conducted in Kenya and Mali. In these studies, a similar approach was taken to exclude persons with clinical evidence of immunosuppressive disease but not test for asymptomatic HIV infection, based on the rationale that it is necessary to assess the safety and immunogenicity of this vaccine in generally healthy persons who are representative of the population from which they are drawn. In Kenya, where rates of HIV infection are much higher, this general population includes many persons living with HIV and a study that excluded them would be of less value. Eventually, it will be necessary to demonstrate the safety and immunogenicity in persons living with HIV for any malaria vaccine to be employed in Africa, but because of the low rates of HIV infection in Mali these studies will have to be conducted elsewhere. Meaningful subanalyses of HIV-infected and uninfected vaccinees are not possible in this small trial.

Voluntary counselling and testing for HIV by rapid test is now available at the Bandiagara District Hospital, and antiretroviral treatment is available at the nearby Mopti District Hospital. In the event that HIV infection is suspected at the time of screening or later in the trial, participants and their parents/guardians will be referred to these sources of HIV testing and treatment.

5.4 Treatment Assignment Procedures

5.4.1 Randomization Procedures

Individual participants will be randomized to receive one of three escalating doses of FMP2.1/AS02A, or rabies vaccine. One hundred participants will be assigned to Cohort 1 (n=20), Cohort 2 (n=40) or Cohort 3 (n=40) in the order they are enrolled with stratification for age by two-year increments (1-2 years, 3-4 years, 5-6 years) but without stratification for gender, and randomized within cohorts to receive FMP2.1/AS02A or rabies vaccine. Because cohort sizes are not divisible by three, the numbers in each stratum may be either 6 or 7 in each age stratum for Cohort 1 and either 13 or 14 in Cohorts 2 and 3. Cohort 1 will be randomized in a 3:1 ratio to receive 10μg of FMP2.1 adjuvanted with 0.1 mL of AS02A (n=15) or rabies vaccine (n=5). Cohort 2 will be randomized in a 3:1 ratio to receive 25μg of FMP2.1 adjuvanted with 0.25 mL of AS02A

(n=30) or rabies vaccine (n=10). Cohort 3 will be randomized in a 3:1 ratio to receive 50μg of FMP2.1 adjuvanted with 0.50 mL of AS02A (n=30) or rabies vaccine (n=10). The total sample size for the rabies group will thus be 25.

Within each cohort, treatments will be assigned to participant ID numbers in randomized blocks of a size that will not be stated in this protocol to avoid compromising masking, per DMID guidelines. Randomization to either of the two vaccines will be done using a computer-generated randomization list. The randomization list will contain sequential codes linking a participant ID number to a vaccine assignment. Participant ID numbers will be assigned to participants of each cohort in the order in which they are enrolled in the trial.

5.4.2 Masking Procedures

Measures will be taken to keep participants, their parents/guardians and clinical investigators (including the PI) and all other staff involved in measuring study outcomes blinded to treatment allocation. Masking procedures are described in SOPs for randomization and vaccine preparation and administration. The FMP2.1/AS02A vaccine will have an opaque milky white appearance. The comparator rabies vaccine will appear as a clear to slightly opaque, colorless suspension after reconstitution. Therefore, blinding of the individuals preparing the study vaccine ("study pharmacists") will not be possible. Since the test article and comparison vaccines can be distinguished by appearance, the vaccine preparation area and the immunizing area will be physically separated. The study pharmacist and his assistant are both experienced pharmacists, and they will be exclusively dedicated to vaccine preparation.

Immunizations will be carried out simultaneously in dedicated vaccination rooms adjacent to the vaccine preparation room and connected by sliding doors. Despite the fact that the volumes of the study vaccine preparations are different, every attempt will be made to maintain blinding. The syringe barrels will be covered with opaque tape. Vaccinators will be physicians who are not involved in any way with surveillance activities, so that even if they realize which vaccine they are injecting, they will not be involved in the assessment of adverse events following vaccination. Each participant will be vaccinated in a closed room out of view of anyone other than the vaccinators, so that each parent sees only the syringe her child is injected with and never sees other participants being injected. The parents will not be told that the vaccines vary with respect to volume.

The Statistician at the EMMES Corporation in the US will also have the randomization list. The Local Medical Monitor will be provided sealed code-break envelopes and may unblind herself to the vaccine allocation of individual study participants if deemed urgently necessary for medical and/or ethical reasons (for example, in the case of a participant in urgent need of rabies immunization). The Local Medical Monitor (LMM) will also keep one set of the randomization code in a sealed envelope, in the event that emergency unblinding is required. USAMMDA will also be provided a sealed copy which will be held by the Division of Medical Affairs. Access to unsealed copies of the randomization list will be exclusively limited to the study pharmacists and the EMMES Corporation statistician. These individuals will be unblinded and will not be involved in study participants' further evaluation.

5.4.3 Reasons for Withdrawal

The following criteria will be checked at each visit. If any become applicable during the study, the subject will not be required to discontinue the study, but a separate immunogenicity analysis may be done that excludes these individuals.

- Use of any investigational drug or vaccine other than the study vaccine(s) during the study period.
- Chronic administration (defined as more than 14 days) of any dose level of immunosuppressants or other immune-modifying drugs during the study period and chronic daily use of inhaled steroids. Intermittent use of inhaled and topical steroids is allowed.
- Administration of a vaccine not foreseen by the study protocol during the period starting from 30 days before the first study immunization and ending 30 days after the third immunization.
- Administration of immunoglobulins and/or any blood products up to 30 days after the last study immunization.

The following criteria will be checked prior to each immunization and are contraindications to further immunization. However, the study participants and their parents/guardians will be encouraged to continue to participate in the surveillance schedule for safety evaluation.

- Systemic hypersensitivity reaction following administration of the study vaccine. Severe (i.e., Grade III) local reactions will be evaluated to determine whether or not further study immunizations should be administered

Subjects may also withdraw voluntarily from receiving the study intervention upon request for any reason.

5.4.4 Handling of Withdrawals

Every effort will be made to collect safety data on any subject discontinued because of an AE or SAE by continuing the safety follow-up procedures. If voluntary withdrawal occurs, the subject will be asked to continue scheduled evaluations and be given appropriate care under medical supervision until the symptoms of any AE resolve or the subject’s condition becomes stable. If possible, subjects who leave the study area will be traced and visited by clinical investigators to collect safety follow-up data.

5.4.5 Termination of Study

The trial may be terminated by USAMMDA, or suspended by DMID, or the PI due to development of serious laboratory toxicities or other major safety concern identified during interim safety analyses

by the DSMB or at any other time. The trial may also be suspended by the IRBs if deemed necessary.

6 STUDY INTERVENTION/INVESTIGATIONAL PRODUCT

6.1 Study Product Description

6.1.1 Acquisition

The candidate antigen, FMP2.1, has been developed and manufactured by WRAIR. The adjuvant AS02A is manufactured by GSKBio. As has been done for the two previous Phase 1 trials at this site of vaccines consisting of WRAIR antigen and GSKBio adjuvant, the antigen will be transported from the US and the adjuvant from Rixensart, Belgium, to the MRTC in Bamako, under documented temperature-controlled conditions. The rabies vaccine will be purchased by the University of Maryland from the manufacturer and shipped to Mali via the same mechanism as the FMP2.1 antigen.

6.1.2 Formulation and Packaging

6.1.2.1 FMP2.1

Each vial of the FMP2.1 antigen contains 50 μg of lyophilized protein.

Active Ingredient: 0.100 mg/mL Formulated Bulk FMP2.1 when reconstituted to 0.60 mL volume

Formulation Buffer: 23.5 mM Sodium phosphate

0.1 mM EDTA

3.15% Sucrose

30 mM Sodium chloride

6.1.2.2 AS02A adjuvant

The FMP2.1 will be reconstituted in AS02A adjuvant. AS02A contains 50 μg MPL, 50 μg QS21, and 0.250 mL of proprietary oil/water emulsion in phosphate buffered saline (PBS) per volume of 0.5 mL. The AS02A adjuvant will be supplied as pre-filled syringes. The prefilled syringes will contain approximately 0.60 mL of liquid and will be stored at 2ºC to 8ºC.

6.1.2.3 RabAvert® vaccine

Our two previous Phase 1 malaria vaccine trials have used the Imovax and RabAvert rabies vaccines, which are very similar products with identical safety profiles, administered on the same schedule. We plan to use the RabAvert rabies vaccine, produced by Chiron Behring GmbH & Co. It

is a sterile freeze-dried vaccine obtained by growing the fixed-virus strain Flury LEP in primary cultures of chicken fibroblasts. The strain Flury LEP was obtained from American Type Culture Collection as the 59th egg passage. The growth medium for propagation of the virus is a synthetic cell culture medium with the addition of human albumin, polygeline (processed bovine gelatin) and antibiotics. The virus is inactivated with propiolactone, and further processed by zonal centrifugation in a sucrose density-gradient. The vaccine is lyophilized after addition of a stabilizer solution which consists of buffered polygeline and potassium glutamate. One dose of reconstituted vaccine contains less than 12 mg polygeline (processed bovine gelatin), 1 mg potassium glutamate and 0.3 mg sodium EDTA. Small quantities of bovine serum are used in the cell culture process. Bovine components originate only from source countries known to be free of bovine spongiform encephalopathy. Minimal amounts of chicken protein may be present in the final product; ovalbumin content is less than 3 ng/dose (1 mL), based on ELISA. Antibiotics (neomycin, chlortetracycline, amphotericin B) added during cell and virus propagation are largely removed during subsequent steps in the manufacturing process. In the final vaccine, neomycin is present at < 1 μg, chlortetracycline at < 20 ng, and amphotericin B at < 2 ng per dose. RabAvert is intended for intramuscular (IM) injection. The vaccine contains no preservative and should be used immediately after reconstitution with the supplied Sterile Diluent for RabAvert (Water For Injection). The potency of the final product is determined by the NIH mouse potency test using the US reference standard. The potency of one dose (1.0 mL) RabAvert is at least 2.5 IU of rabies antigen. RabAvert is a white, freeze-dried vaccine for reconstitution with the diluent prior to use; the reconstituted vaccine is a clear to slightly opaque, colorless suspension.

6.1.3 Product Storage and Stability

The FMP2.1 antigen, rabies vaccine, and AS02A pre-filled syringes will be stored at 2 to 8 ºC. Vaccines will be kept in a cold room or refrigerator that has 24-hour temperature recording. A back-up refrigerator and generator will be available in case of breakdown/power failure. The refrigerator that holds the vaccines and adjuvant will be locked. Records will be maintained that document receipt, release for immunization, disposal or return to the manufacturer of all vaccine vials. Copies of these records will be provided to the sponsors upon request for archiving. All study records will be kept in locked metal boxes.

The FMP2.1 antigen vials will be transported under controlled temperature conditions to the MRTC in Bamako, Mali. Temperature recorders will document maintenance of required temperature ranges. The AS02A adjuvant will be similarly transported from Belgium. FMP2.1 antigen, comparator vaccine and adjuvant will be stored in a cold room in the MRTC laboratory in Bamako until a few days before each vaccination is scheduled in Bandiagara. These will be transported in temperature-controlled conditions from Bamako to Bandiagara in the same containers used for shipping to Mali.

6.2 Dosage, Preparation and Administration of Investigational Product

The Investigators will maintain detailed SOPs for vaccine transport, storage, formulation, reconstitution and administration. Staff and investigators will be trained by senior investigators, the clinical coordinator and/or the senior study pharmacist in the SOPs relevant to their duties and will sign copies of the SOPs to document this training. Copies of SOPs will be available for inspection and review by the DMID, USAMMDA, GSKBio, WRAIR and study monitors. The study pharmacist and assistant study pharmacist will bear sole responsibility for storing, preparing, monitoring, and reconstituting vaccine and adjuvant from the time of delivery to the MRTC in Mali to the time syringes containing reconstituted vaccine and adjuvant are passed to physicians to administer to participants.

6.2.1 Administration of vaccines

Vaccines will be administered by physician-investigators not involved with assessing adverse events. Each 0.1, 0.25 or 0.5 mL of FMP2.1/AS02A or 1.0 mL of rabies vaccine will be administered by intramuscular injection in the left deltoid muscle immediately after formulation/reconstitution. Each vial will be used for only one dose for one participant. Alternatively the right deltoid muscle may be used when the preferred site for injection is contraindicated or not advisable such as in the case of a condition involving pain, infection, scarring that will make local reactions difficult to assess, or if the study participant or parent/guardian declares a preference for immunization in the alternative site.

For each cohort, immunizations will begin with children aged 2 years or older. Children aged less than two years will not be immunized until at least a day after the first immunizations are given to older children.

6.3 Accountability Procedures for the Investigational Product

In Mali, only the vaccine manager and assistant vaccine manager will have access to vaccines. The Vaccine Log Book will be used to record use and final disposition of each vial of FMP2.1 and adjuvant. Used vaccine vials, as well as unused vials, will be kept until such time as the PI and USAMMDA agree that there are no concerns about vaccine accountability and that they can be destroyed or returned according to instructions from USAMMDA.

6.4 Concomitant Medications/Treatments

At each study visit/contact, the investigator will question the participant and their parents/guardians about any medication taken, including traditional medicines. Concomitant medication, including any vaccine other than the study vaccines, and any other medication relevant to the protocol, including any specifically contraindicated or administered during the period starting from one week before each study immunization and ending one month (maximum 30 days) after will be recorded in the

CRF with trade name and/or generic name of the medication, medical indication, start and end dates of treatment.

7 STUDY SCHEDULE

7.1 Screening

Recruitment will be progressive until 100 children of either gender who fulfill the inclusion criteria are included. Participants will be recruited among children aged 1-6 years of age residing in Bandiagara. They will be recruited after their parents voluntarily bring them to the BMP clinic. After the study has been explained to the parents of the potential participants, they may leave and return later with their decision; this will allow time for them to discuss the study with their family and carefully consider their child's involvement in the study. Finally, at the BMP clinic, the individual consent process will be conducted in private room to ensure confidentiality.

After community information is disseminated as described in Section 15.3, all interested parents of potentially eligible children will be invited to visit the study clinic on a specific date. They will receive oral and written explanation of the study, after which screening consent will be obtained from those willing to participate. All screening tests, medical history and examinations will be performed only after screening consent is obtained. Study clinicians will generally handle acute, simple conditions such as malaria or other acute infections. More complicated or chronic conditions, such as chronic renal or heart disease, will be referred to appropriate sources of medical care.

A screening form will be prepared for each participant and will later become part of the CRF for participants enrolled in the vaccine trial. A unique screening identification number will be assigned to each study participant. A medical history will be taken with special attention to recurrent infections to suggest immune suppression, previous history of splenectomy and prior vaccine reactions. Physical examination and laboratory screening tests will include: complete blood count (CBC) creatinine, ALT, and urine dipstick analysis. Unused blood from screening tests may be preserved for parasite genotyping. A participant who meets any of the exclusion criteria will be excluded. Participants excluded from this study because of significant abnormalities will be managed initially by study clinicians and referred to the local health center for evaluation as necessary. Screening tests will be completed within 35 days prior to entry into the study. Laboratory studies may be conducted at other times during the course of the trial if the investigators judge it necessary for the safety of the participant. Screening and follow-up diagnostic laboratory testing will be performed at the MRTC laboratory in Bandiagara and if applicable at the MRTC clinical laboratory in Bamako. Recruitment will continue until 100 eligible participants have fulfilled all of the inclusion criteria and none of the exclusion criteria, and signed the study consent form. Should parents of participants change their mind and decline to participate prior to immunization, additional participants will be screened until 100 participants are enrolled. At the end of the screening process, a list of eligible children will be generated and used to identify the participants eligible for immunization.

7.2 Enrollment/Baseline

Participant ID numbers will be assigned to participants of each cohort in the order in which they come to BMP clinic on the day of immunization. Immediately after the first immunization, each study subject will receive a photo ID card. The vaccine and dose that are assigned during the first immunization will be maintained for second and third immunizations.

7.3 Vaccination process

Before each vaccination, criteria for continued eligibility will be reviewed and verified. A history-directed physical examination will be done and temperature, blood pressure, pulse, respiratory rate and baseline general symptoms will be recorded. Venous blood will be collected for laboratory analysis.

After the participant's identity is checked by comparing his/her name with the list of eligible children, he/she will be vaccinated by intramuscular injection into the left deltoid muscle. If any local impairment prevents administration of the vaccine into the preferred deltoid for that particular immunization, the vaccine may be administered into the opposite deltoid. Vaccination will be done on study days 0, 30 +/- 7 and 60 +/- 7.

The participant ID number will be assigned only to children immunized with the first dose. After immunization, the ID photo card with the unique participant ID number assigned to children will be prepared.

7.3.1 Dose escalation

As soon as possible after the completion of the 7-day surveillance period following each of the first two immunizations of cohorts 1 and 2, the DSMB will review safety results and recommend to the sponsors whether to proceed with next scheduled immunization and with the 25 and 50 µg doses for cohorts 2 and 3, respectively. Ultimately, the decision to proceed with each dose escalation will be made by USAMMDA and DMID based on advice from the DSMB and in consultation with WRAIR, GSKBio, USAID, and the investigators. The timeline for safety reviews by the DSMB is presented in detail in Section 10.11.

7.4 Follow-up

After each immunization, participants will be observed for local and systemic reactions for a minimum of 60 minutes. Signs and symptoms will be solicited from participants and parents/guardians and recorded by the investigators. Participants will then be followed for a 7-day surveillance period after each vaccination (including day 0, the day of vaccination). All adverse events occurring during this 7-day period will be followed until resolution or stabilization. If any symptom persists beyond the 7-day surveillance period the participant will be followed daily until resolution of the adverse event.

After the 7-day surveillance period, participants and their parents/guardians will be asked to come to the BMP clinic center on days 14 +/- 3 and 30 +/- 7 after vaccination. At each visit, a study physician will evaluate the participants. A clinical examination will be performed and information on any solicited or unsolicited symptoms since the last visit will be collected. Every effort will be made to ensure compliance with visits. Local guides will conduct home visits to participants a day before their scheduled visit at the clinic. If a participant does not appear for a scheduled clinic visit, the local guide will visit him/her again and accompany the participant to the clinic center. If a serious adverse event (SAE) has occurred, appropriate measures will be taken to notify the PI, Local Medical Monitor, DSMB, USAMMDA, DMID, and all IRBs as described in Section 10.7.2.

8 STUDY PROCEDURES/EVALUATIONS

8.1 Standard operating procedures (SOPs)

The Investigators will maintain detailed SOPs for vaccine transport, storage, formulation, reconstitution and administration. All staff and investigators will be trained in the SOPs relevant to their duties and will sign copies of the SOPs to document this training. Copies of SOPs will be available for inspection and review by the DMID, USAMMDA, GSKBio, WRAIR and study monitors. During the study SOPs may be modified to improve them and new SOPs may be developed as needed to improve operations and ensure adherence with the protocol.

The following SOPs will be followed (additional SOPs may be developed and added):

- Recruitment
- Community informed consent
- Individual informed consent
- Field team organization
- Physical examination
- Management of anaphylaxis
- Case management of uncomplicated and severe malaria
- Management of abnormal laboratory tests
- Completion of CRFs
- Transport of vaccines and adjuvants
- Vaccine preparation and administration
- Vaccine accountability, handling, storage, disposition and destruction
- Randomization
- Unblinding
- Quality control and quality assurance
- Study documentation
- Adverse event reporting
- Protocol deviations
- SAE reporting
- Provisions for study suspension
- Malaria thick smear preparation and reading
- Specimen collection and handling
    - Blood collection
    - Urine collection
    - Specimen transport
    - Specimen reception
    - Serum separation
    - Specimen storage

    - Specimen shipping overseas
- Waste management
    - Sharps handling and disposal
    - Biologic waste handling and disposal
    - General waste handling and disposal
- Safety
    - Spill management
    - Exposure to body fluids
- Equipment maintenance and quality control SOPs (Reflotron PLUS, AcT-Diff, Centrifuge)

8.2 Daily study procedures

Day -35 to –1 Screening /inclusion of participants

Meetings will be held with town administrative and medical authorities to explain the purpose of the study. These meetings will be followed by meetings with the traditional authorities and the heads of families for village-level "permission to enter." Subsequently, general information about the study will be disseminated through the local radio station. The target population will be invited for screening as described in investigator's subject recruiting SOPs. Screening will be performed until 100 eligible participants are identified.

Screening (may take place over more than one visit) up to 35 days prior to vaccination

- Written informed consent for screening
- Medical history of participant
- Complete physical examination
- Collect 2-3 mL venous blood sample to measure:
    - Hematology: Complete Blood Count (CBC) which includes: red blood cells, platelets, white blood cell count, lymphocyte count, hemoglobin, mean corpuscular volume and mean corpuscular hemoglobin concentration.
    - Biochemistry: serum creatinine and ALT
    - Remaining unused blood may be used for parasite genotyping
- Collect urine: dipstick for blood, glucose and protein
- Check of inclusion and exclusion criteria
- Written informed study consent for vaccination

Day 0: Vaccination 1

Before vaccination:

- Review screening laboratory test results
- Review inclusion/exclusion criteria and check of contraindications/precautions
- Record any complaints, symptom-directed physical examination, and examination of the immunization site(s) for any abnormalities.
- Record vital signs: temperature, blood pressure, pulse, respiratory rate

- Record baseline data for solicited general symptoms
- Collect up to 7-10 mL venous blood sample to measure:
    - CBC, creatinine, ALT
    - Serum for anti-FMP2.1 antibodies
    - Peripheral blood mononuclear cells (PBMC) for cell-mediated immune responses (CMI) to AMA-1
- Assignment of unique participant ID number
- Administer study immunization 1

After vaccination:
- Observe for a minimum of 60 minutes
- Record blood pressure, pulse, respiratory rate, temperature
- Record solicited and unsolicited events
- Prepare an ID card containing participant's unique participant ID number and photo
- Instruct participants and their parents/guardians to return to the BMP clinic center immediately should they manifest any signs or symptoms they perceive as serious.

Days 1-3: Days 1, 2, 3 post-vaccination 1 surveillance visits
- Brief medical history
- Record temperature
- Record solicited and unsolicited signs/symptoms
- Targeted physical examination including immunization site

Day 7± 1 day: 7 days post-vaccination 1 surveillance visit
- Brief medical history
- Record temperature
- Targeted physical examination including immunization site
- Record any solicited and unsolicited adverse events occurring after the last study immunization
- Collect 2-3 mL of venous blood to measure CBC, creatinine and ALT

Day 14± 3 days: 14 days post-vaccination 1 surveillance visit
- Brief medical history
- Record temperature
- Targeted physical examination including immunization site
- Record any unsolicited adverse events occurring after the last study immunization

Day 30± 7 days: 1 month post-vaccination 1 surveillance visit and vaccination 2

Before vaccination:
- Check participant's ID to confirm identity
- Brief medical history
- Targeted physical examination including immunization site(s)
- Check of contraindications/precautions

- Record vital signs: temperature, blood pressure, pulse, respiratory rate
- Review medical history and record any unsolicited adverse events occurring since last visit
- Record baseline data for solicited general symptoms
- Collect up to 7-10 mL venous blood sample to measure:
    - CBC, creatinine, ALT
    - Serum for anti-FMP2.1 antibodies
    - PBMC for CMI
- Administer study immunization 2

After vaccination:

- Observe for at least 60 minutes
- Record blood pressure, pulse, respiratory rate, temperature
- Record solicited and unsolicited adverse events
- Instruct participants and their parents/guardians to return to the BMP clinic center immediately should they manifest any signs or symptoms they perceive as serious.

Days 31-33: Days 1, 2, 3 post-vaccination 2 surveillance visits

- Brief medical history
- Record temperature
- Record solicited and unsolicited signs/symptoms
- Targeted physical examination including immunization site(s)

Day 37 ± 7 days: Day 7 post-vaccination 2 surveillance visit

- Brief medical history
- Record temperature
- Record solicited and unsolicited signs/symptoms
- Targeted physical examination including immunization site(s)
- Collect 2-3 mL of venous blood to measure CBC, creatinine and ALT

Day 44± 7 days: 14 days post-vaccination 2 surveillance visit

- Brief medical history
- Record temperature
- Targeted physical examination
- Record any unsolicited adverse events occurring after the last study immunization

Day 60± 7 days: 1 month Post-vaccination 2 surveillance visit and vaccination 3

Before vaccination:

- Check participant’s ID to confirm identity
- Brief medical history
- Targeted physical examination including immunization site(s)s
- Check of contraindications/precautions
- Record vital signs: temperature, blood pressure, pulse, respiratory rate

- Review medical history and record any unsolicited adverse events occurring since last visit
- Record baseline data for solicited general symptoms
- Collect up to 7-10 mL venous blood sample to measure:
  - CBC, creatinine, ALT
  - Serum for anti-FMP2.1 antibodies
  - PBMC for CMI
- Administer study immunization 3

After vaccination:

- Observe for at least 60 minutes
- Record blood pressure, pulse, respiratory rate, temperature
- Record solicited and unsolicited adverse events
- Instruct participants and their parents/guardians to return to the BMP clinic center immediately should they manifest any signs or symptoms they perceive as serious.

Days 61-63: Days 1, 2, 3 post-vaccination 3 surveillance visits

- Brief medical history
- Record temperature
- Record solicited and unsolicited signs/symptoms
- Targeted physical examination including immunization site(s)

Day 67 ± 7 days: Day 7 post-vaccination 3 surveillance visit

- Brief medical history
- Record temperature
- Record solicited and unsolicited signs/symptoms
- Targeted physical examination including immunization site(s)
- Collect 2-3 mL of venous blood to measure CBC, creatinine and ALT

Day 74± 7 days: 14 days Post-vaccination 3 surveillance visit

- Brief medical history
- Record temperature
- Targeted physical examination including immunization site(s)
- Record any unsolicited adverse events occurring after the last study immunization

Day 90± 10 days: 30 days Post-vaccination 3 surveillance visit

- Brief medical history
- Record temperature
- Targeted physical examination including immunization site(s)
- Record any unsolicited adverse events occurring since last visit
- Collect up to 7-10 mL of venous blood to measure:
  - Serum for anti-FMP2.1 antibodies
  - PBMC for CMI

  - CBC, creatinine and ALT

Day 120± 14 days: 60 days Post-vaccination 3 surveillance visit

- Brief medical history
- Record temperature
- Targeted physical examination including immunization site(s)
- Record any serious adverse events occurring after the last study immunization

Day 180± 14 days, Day 272± 14 days, Day 364± 14 days: Post-vaccination Safety surveillance period

- From this date, participants will be visited monthly by local guides to confirm their location and to remind them to come to clinic for routine clinical evaluation.
- Participants and their parents/guardians are encouraged to continue to attend the BMP clinic any time they are sick.
- From this day participants and their parents/guardians will be asked to return to BMP clinic center every 3 months ±14 days.
- Brief medical history
- Record vital signs
- Targeted physical examination including immunization site(s)
- Collect up to 7-10 mL venous blood to measure:
  - Serum for anti-FMP2.1 antibodies
  - PBMC for CMI
- Participants and their parents/guardians are invited to continue to attend the BMP clinic any time they are sick. A malaria smear will be done whenever symptomatic malaria is suspected.

8.2.1 Outline of study procedures

Table 10. Summary of study procedures

**Study Days**	**-35 to –1 Screening**	**0**	**1-3**	**7**	**14**	**30**	**31-33**	**37**	**44**	**60**	**61-63**	**67**	**74**	**90**	**120**	**180-364**
**Clinic Visit**	**1**	**2**	**3-5**	**6**	**7**	**8**	**9-11**	**12**	**13**	**14**	**15-17**	**18**	**19**	**20**	**21**	**22-24**
Village & family information & discussion	●															
Written individual Screening Consent	●															
Check of inclusion/exclusion criteria	●	●														
Check of contraindications to immunization		●				●				●						
Written individual Study Consent		●														
Medical history	●	●	●	●	●	●	●	●	●	●	●	●	●	●	●	●
Physical examination	●	●	●	●	●	●	●	●	●	●	●	●	●	●	●	●
**Vaccination**		●				●				●						
Post-vaccination recording of solicited AE		●	●	●		●	●	●		●	●	●				
Recording of unsolicited AE occurring up to one month post-vaccination			●	●	●	●	●	●	●	●	●	●	●	●		
Recording of medication		●	●	●	●	●	●	●	●	●	●	●	●	●		
Recording of SAEs during the study period		●	●	●	●	●	●	●	●	●	●	●	●	●	●	●
Urine analysis for blood, glucose and protein	●[a]															
CBC	●[a]	●[b]		●		●[b]		●		●[b]		●		●		
Serum chemistry (Creatinine, ALT)	●[a]	●[b]		●		●[b]		●		●[b]		●		●		
Serum and cells for anti-AMA-1 response		●[b]				●[b]				●[b]				●		●[c]
Monthly home visit by local guides															●[d]	●[d]
Scheduled blood volume (mL)	2-3	7-10	0	2-3	0	7-10	0	2-3	0	7-10	0	2-3	0	7-10	0	21-30
Cumulative Blood Volume[e] (mL)	2-3	9-13	9-13	11-16	11-16	18-26	18-26	20-29	20-29	27-39	27-39	29-42	29-42	36-52	36-52	57-82

a. Performed within 35 days prior to immunization
b. Blood collected just prior to each immunization.
c. Serum for anti-FMP2.1 antibodies and cells for CMI collected every 3 months from study Day 180 through study Day 364
d. Monthly local guides home visits to check the status of participants starting from study Day 120
e. A few drops of the drawn venous blood will be blotted onto filter paper for later molecular analyses each time blood is drawn, as well as each time malaria smears are prepared. Unused blood left over after serum chemistries and CBCs are performed may also be preserved for parasite genotyping. This will not increase the amount of blood taken from participants.

8.2.2 Health care provision

Routine and emergency health care will be provided by study clinicians at the Bandiagara District Hospital, in collaboration with District Hospital physicians. The clinical research facility includes private consultation rooms, a procedure room, a resuscitation suite with oxygen, suction and resuscitation kits, and a post-immunization observation room. An ambulance with suction and oxygen will be on site on immunization days. The pharmacy at the BMP clinic will have sufficient

provisions to provide participants with oral and parenteral drugs for the treatment of common illnesses (including uncomplicated and severe malaria) free of charge, using essential medicines and treatment regimens that meet or exceed standards recommended by the Mali Ministry of Health. Blood transfusion will be available at all times. Twenty-four hour hospitalization and basic emergency surgery services are available at the Bandiagara Hospital adjacent to the BMP clinic, in a pediatric ward that is being renovated and upgraded for this trial. Twenty-four hour nursing staffing and 24-hour on call physicians will be available.

If the investigators or the Local Medical Monitor judge that a participant requires hospitalization at the National Hospital in Bamako (approximately 8 hours drive from Bandiagara), referral and transportation will be arranged and the medical management of the participants will be monitored by senior physician investigators and/or the Local Medical Monitor. The regional hospital in Mopti, a 45-minute drive from Bandiagara, has radiography, medical subspecialty and surgical capabilities. The national hospital in Bamako has these capacities and in addition has an intensive care unit with mechanical ventilation, a computerized axial tomography scanning facility and other advanced medical and surgical care.

Cases of clinical malaria will be managed according to Malian National Malaria Control guidelines and the SOP “Case management of uncomplicated and severe malaria”.

8.3 Final Study Visit

At the time of the final study visit (day 364 +/- 14 days), participants and their parents/guardians will be debriefed, including instruction to return to the BMP clinic at any time in the future if they feel they may have a medical problem related to their participation in the study. Any AEs or SAEs that are unresolved at that time will be continue to be followed until resolution, or, if a chronic condition has developed, until it has stabilized.

8.4 Early Termination Visit

If early termination after the first vaccination occurs more than one week after a scheduled visit has been completed and the participant and their parents/guardians are willing to have evaluations performed, a physical examination should be performed and, if more than two weeks have elapsed since the last time venous blood was drawn, up to 7-10 mL venous blood may be drawn for CBC, creatinine and ALT determination, and/or for serology. Any participant who has received one or more immunizations will be followed for the duration of the study even if it is determined that the second or third immunization should not be administered. Participants who exit the study after receiving one or more immunizations will not be replaced by new participants.

8.5 Unscheduled Visit

Unscheduled visits will prompt a history and physical examination, clinical laboratory tests including malaria smear if indicated, documentation of any AEs, and any other medically indicated diagnostic or therapeutic procedures. These will be recorded as observations in the participant’s study record.

9 Sample handling and analysis

9.1 Overview of sample collection, handling, transport and shipping.

Detailed SOPs are maintained for these activities. Briefly, finger-prick blood and venous blood are obtained at the BMP research clinic and processed in the sample processing laboratory according to SOPs. Filter paper blood samples are stored at room temperature in sealed dessicant pouches, and sera, plasma, cells and parasites are frozen at -80°C or in liquid nitrogen containers. Frozen samples are transported in project vehicles to the main MRTC immunology laboratory in Bamako in liquid nitrogen dry shippers, and either stored there in a liquid nitrogen storage system or shipped to the CVD in dry shippers. This cold chain has been successfully maintained since 1999.

9.2 Overview of collection time points

Blood will be collected from study participants by venipuncture up to 11 times during the study, including screening. The maximum amount of blood requested from any participant for standard collection during the study for research purposes will not exceed 90 mL over the one-year study period. However, additional blood may be obtained as deemed necessary by the investigators or clinicians to evaluate a medical illness or condition.

9.2.1 Safety

Tests for CBC, creatinine, and ALT, at screening, and on study days 0, 7, 30, 37, 60, 67 and 90 (intervals between study visits may deviate from this schedule by the ranges indicated in Section 8 above) will be performed at the MRTC clinical laboratory in Bandiagara, with back-up testing (serological testing for hepatitis and other conditions, liver function tests, etc.) available at the MRTC clinical laboratory in Bamako if needed.

9.2.2 Serology

Separation of serum/plasma from venous blood will be performed at the BMP clinic and samples will be aliquoted for later use to determine anti-FMP2.1 antibody assays at Day 0, 30, 60, 90, 180, and 364 (intervals between study visits may deviate from this schedule by the ranges indicated in Section 8 above). The antibody assays on samples collected at day 90 and thereafter will provide information on the duration of vaccine-boosted antibody responses.

9.2.3 CMI

CMI assays will be performed on PBMC samples collected on Day 0, 30, 60, 90, 180, and 364 (intervals between study visits may deviate from this schedule by the ranges indicated in Section 8

above). The CMI assays on samples collected at day 90 and thereafter will provide information on the duration of vaccine-boosted CMI responses.

9.3 Laboratory Assays

The Investigators will maintain detailed SOPs for all laboratory assays at the BMP and central laboratories at MRTC, in Bamako. These SOPs will include sample collection, handling (e.g. serum separation), labeling, preservation (e.g. PBMC cryopreservation), storage, transport, and shipping. All staff and investigators will be trained in the SOPs relevant to their duties and sign copies of the SOPs to document this training. Copies of SOPs will be available for inspection and review by study monitors. The general methods that will be used are summarized in the following section.

9.3.1 Hematology and biochemistry

A complete blood count (CBC) with automated differential cell count, serum creatinine and ALT tests will be measured at defined time points throughout the study period. The Principal Investigator will maintain laboratory reference intervals in the study file, and copies will be made available upon request to study monitors and sponsors. Hematology and serum biochemistry assays will be performed at the MRTC clinical laboratory in Bandiagara. In the rare event that a participant has traveled to Bamako, hematology and biochemistry tests may also be done in the MRTC laboratory in Bamako under study-specific SOPs.

Urine will be collected for glucose, blood and protein determination using FDA-approved urinary reagent dipsticks.

9.3.2 Serology

Serological assays for anti-FMP2.1 antibodies will be performed at the WRAIR laboratories in Silver Spring, US, following SOPs for methods that have been used for the two previous trials of this vaccine.

Immunogenicity will be determined by evaluating antibody (IgG) responses to the *P. falciparum* AMA-1 protein as measured using standard ELISA methodologies with appropriate capture antigens, namely the recombinant AMA-1 antigen that the vaccine is based upon. These results will be the main immunogenicity outcome measure for this trial. Secondary serological assays described below will not be begun until all primary humoral immunogenicity studies have been completed at WRAIR.

9.3.3 Malaria smears

Blood smears for malaria diagnosis by microscopy will be prepared and read when clinically indicated as part of routine medical care. Depending on when immunization starts relative to the malaria transmission season we expect 20-80% of children to have asymptomatic parasitemia. The value of a malaria smear in the absence of symptoms is of no value for safety assessment or

clinical purposes. This trial is not powered to measure prevalence of infection or incidence of malaria disease, data on which for this site are published (5). However, because asymptomatic infection at the time of immunization or in the period following immunization could influence immune responses to the vaccine (in either direction), blood smears for microscopy and filter paper samples for PCR detection of infection will be collected at each time point when venous blood is drawn and stored for retrospective analyses to determine whether asymptomatic infection accounts for variations in the immune response. It is emphasized that malaria microscopy and PCR results do not constitute trial endpoints nor will they be reported as such.

9.3.4 Additional assays

Titers of AMA-1 antibodies measured by ELISA will be the main immunological endpoint considered in choosing a dose level of FMP2.1/AS02A to take forward into the subsequent Phase 2 trial. However, several other means of measuring the immune response are being assessed. These additional studies may help refine our understanding of the immune response to AMA-1 vaccines in ways that lead to development of improved immunogenicity assays for future vaccine trials. These immunological studies, in conjunction with AMA-1 sequencing studies and related in vitro studies conducted separate from this protocol, are aimed to improve our understanding of the degree to which the immune response to AMA-1 immunization is allele-specific or cross-reactive. These studies may inform further development and optimization of AMA-1 vaccines. To ensure appropriate use of sera and PBMC, these additional assays will be done in an order of priority agreed upon by the investigators.

9.3.5 CMI and antibody avidity assays

PBMCs will be cryopreserved and transported to the University of Maryland for CMI assays. The objectives of CMI and secondary serological studies are twofold: (1) Accurately measure humoral immunity by quantifying antibody responses to the antigen AMA-1 and (2) Systematically analyze T-cell responses to both full-length recombinant AMA-1 (rAMA-1) antigen and to synthesized peptides derived from domain I of the AMA-1 ectodomain. Based on these results, we will address three important questions: (1) Is the level of anti-AMA-1 antibody levels in serum (as measured in end-point dilutions by ELISA) correlated with T-helper cell responses? (2) What are the characteristics of memory T cell responses? And (3) are the avidity and/or classes and IgG sub-classes of anti-AMA-1 antibodies correlated with memory and effector T-helper cell responses?

In addition to antibody levels as measured by ELISA titers, avidity of antibodies may be important measures of a protective immune response. Antibody avidity and affinity assays will be performed at the University of Maryland to test the hypothesis that the avidity of antibodies to AMA-1 correlates with allele-specific T-helper cell responses to AMA-1.

A list of assays follows. Results of these studies will not be used as measures of immunogenicity for purposes of choosing a dose for further clinical development. Methods for some of these assays are currently being developed and refined. As noted above, these assays will be prioritized by the investigators to ensure optimal use of limited sera and cells.

- Antibody avidity studies
- Identification of immunodominant T cell epitopes involved in class I (e.g., CTL) and II (e.g., Th1, Th2) MHC restricted T cell responses
- Measurement of cytokine-producing cells to soluble antigens by ELISPOT
- Cytokine measurements using optimized Cytometric Bead Array (CBA) technology
- Measurement of memory T cell subpopulations
- Measurement of memory B cell pools
- Lymphoproliferative responses to AMA-1

9.3.6 Additional serology

As a capacity-building exercise, WRAIR investigators will assist MRTC investigators with establishing the ability to perform serological assays for antibody (IgG) responses to the P. falciparum AMA-1 by using the same ELISA methodologies with appropriate capture antigens that will be used for the immunogenicity study endpoint at WRAIR. This process has begun with a 3-month visit by an MRTC scientist to the WRAIR ELISA laboratory in 2005.

No additional blood will be drawn for these capacity-building assays. After final serological results from the reference immunology laboratory at WRAIR have been fully analyzed and reported, and if sufficient serum remains, these capacity building assays may be conducted and the results of the Mali assays may be compared to the WRAIR serological results, solely for the purposes of assessing how well the Malian laboratory was able to replicate the WRAIR results. It is emphasized that this is a capacity-building exercise and that only the serological results from the WRAIR Department of Immunology will be analyzed as trial endpoints.

9.3.7 AMA-1 sequencing and genotyping

To the extent that the vaccine effect is allele-specific, we hypothesize that the frequency of malaria infections with AMA-1 sequence similar to the 3D7 vaccine strain would decrease in the FMP2.1/AS02A group relative to the control group in a Phase 2 study that is powered for this type of analysis. This Phase 1 trial is not powered to measure efficacy of any type, whether clinical or molecular. However, we may be able to detect trends in frequencies of polymorphisms that will allow us to define specific haplotypes based on particular subsets of the 31 polymorphic amino acid residues that appear to be under selection pressure by the vaccine. These preliminary data will then be used to develop an analysis plan for measuring molecular efficacy in the Phase 2 trial.

At any scheduled or unscheduled visit when clinical malaria is suspected, microscopy for malaria will be performed as a clinical diagnostic procedure for routine medical care. Each time malaria smears are obtained by digital puncture, a few drops (less than 0.2 mL) of blood will be blotted onto

filter paper and preserved for parasite DNA analysis. Unused blood left over after clinical laboratory testing (serum chemistries and CBC) have been done may also be used for parasite genotyping. These samples will be subject to sequencing of AMA-1 to identify polymorphic amino acid residues that may be selected by the FMP2.1/AS02A vaccine and other parasite genotyping studies including microsatellite analysis to aid with characterization of vaccine-resistant strains in future studies. These genotyping studies are expected to guide the development of analytical plans for measuring efficacy at the molecular level in the subsequent Phase 2 trial of this vaccine.

10 ASSESSMENT OF SAFETY

10.1 Specification of Safety Parameters

The primary outcome measures for this trial are:

1. Occurrence of solicited symptoms after each vaccination during a 7-day surveillance period (day of vaccination and days 1, 2, 3 and 7 after vaccination)

2. Occurrence of unsolicited symptoms after each vaccination during a 30-day surveillance period (day of vaccination and 30 subsequent days)

3. Occurrence of serious adverse events throughout the study period

10.2 Methods and Timing for Assessing, Recording, and Analyzing Safety Parameters

10.2.1 Adverse Events

An adverse event includes any noxious, pathological or unintended change in anatomical, physiological or metabolic functions as indicated by physical signs, symptoms and/or laboratory detected changes occurring in any phase of the clinical study whether associated with the study vaccine or active comparator vaccine and whether or not considered vaccination related. This definition includes an exacerbation of pre-existing conditions or events, intercurrent illnesses, or vaccine or drug interaction. Symptomatic uncomplicated or severe malaria infection will be coded as adverse events as will any other acute illness, throughout the 15 months of the study. Anticipated day-to-day fluctuations of pre-existing conditions that do not represent a clinically significant exacerbation need not be considered adverse events. Discrete episodes of chronic conditions occurring during a study period will be reported as adverse events to assess changes in frequency or severity.

Adverse events will be documented in terms of a medical diagnosis. When it is not possible to make a specific medical diagnosis, the adverse event will be documented in terms of signs and/or symptoms observed by the investigator or reported by the subject at each study visit. Pre-existing conditions or signs and/or symptoms (including any that are not recognized at study entry but are recognized during the study period) present in a participant prior to the start of the study will be recorded on the participant's CRF. Any hospitalization will be considered a serious adverse event. Adverse events to be recorded as endpoints are described below. All other adverse events will be recorded as unsolicited adverse events.

10.2.2 Surveillance period for adverse events

All adverse events occurring within 30 days following administration of each study immunization will be recorded irrespective of severity or whether or not they are considered vaccination-related.

Solicited adverse events will be elicited for a 7-day surveillance period (day of vaccination and study days 1, 2, 3 and 7) and unsolicited adverse events will be recorded during a 30-day surveillance period. Serious adverse events will be recorded throughout the study.

10.2.3 Recording adverse events

At each visit/assessment, the investigator will evaluate all adverse events observed by the investigators or reported by the participant or their parents/guardians. New adverse events will be recorded in the Adverse Event form within the participant's CRF. Solicited and unsolicited adverse events will be recorded on separate pages of the CRF. The nature of each event, date and time (where appropriate) of onset, outcome, intensity and relationship to vaccination will be established. Any corrective treatment will be recorded in the CRF. As a consistent method of soliciting adverse events, the participant and their parents/guardians will be asked a non-leading question such as: "Have you felt different in any way since receiving the vaccine or since the last visit?" The investigator will record only those adverse events having occurred within the time frames defined above.

Adverse events already documented in the CRF, *i.e.* at a previous assessment and designated as 'ongoing' will be reviewed at subsequent visits, as necessary. If these events have resolved, the documentation in the CRF will be completed, including the date that the adverse event resolved. If an adverse event changes in frequency or intensity during a study period, the record will be updated to reflect the maximum intensity or describe the frequency.

10.2.4 Solicited adverse events

Local (injection site) adverse events
Pain or tenderness at injection site
Swelling at injection site
Erythema at injection site

General adverse events
Fever (body temperature ≥ 37.5°C)
Drowsiness
Loss of appetite
Vomiting
Irritability/fussiness

Temperature will be recorded at the time of the clinic visit. If additional temperature measurements are recorded at another time of the day, the highest temperature will be recorded.

10.2.5 Unsolicited adverse events

Unsolicited adverse events will be recorded in the CRF. Unsolicited adverse events are adverse events reported by the participants or accompanying guardians that are different from those solicited symptoms defined in the preceding section and/or that begin after the 7-day surveillance period for solicited adverse events.

10.3 Assessment of intensity of non-serious adverse events

For each solicited symptom the participants and their parents/guardians will be asked if they sought medical advice for this symptom. For all other adverse events than those in Table 11, maximum intensity will be assigned to one of the following categories:

0 = No adverse event

1 = An adverse event which is easily tolerated by the subject, causing minimal discomfort and not interfering with everyday activities.

2 = An adverse event that is sufficiently discomforting to interfere with normal everyday activities.

3 = An adverse event that prevents normal, everyday activities. Such an adverse event would for example prevent normal play or attendance at school and would require the administration of corrective therapy.

Intensity of the following adverse events will be assessed as described in Table 11:

Table 11. Assessment of Solicited Adverse Event (AE) Intensity

AE	Grade	Intensity Definition
Pain/tenderness at injection site	0	Absent
	1	Minor reaction to touch
	2	Cries/protests on touch
	3	Cries when limb is moved/spontaneously painful
Swelling at injection site	0	Absent
	1	< 5 mm
	2	5-20 mm
	3	> 20 mm
Erythema at injection site	0	Absent
	1	< 5 mm
	2	5-20 mm
	3	> 50 mm
Limitation of arm motion - Abduction at the shoulder	0	None
	1	>90° but <120°
	2	>30° but ≤90°
	3	≤30°
Fever	0	< 37.5°C
	1	37.5-38.0°C
	2	38.1-39.0°C
	3	> 39.0°C
Irritability/fussiness	0	Behavior as usual
	1	Crying more than usual/ no effect on normal activity
	2	Crying more than usual/ interferes with normal activity
	3	Crying that cannot be comforted/ prevents normal activity
Drowsiness	0	Behavior as usual
	1	Drowsiness easily tolerated
	2	Drowsiness that interferes with normal activity
	3	Drowsiness that prevents normal activity
Loss of appetite	0	Normal
	1	Eating less than usual/ no effect on normal activity
	2	Eating less than usual/ interferes with normal activity
	3	Not eating at all
Vomiting	0	Absent
	1	Occasional but able to eat/drink normal amounts
	2	Repeated with limited oral intake
	3	Continuous, unable to keep down liquids or solids

Table 12. Laboratory toxicity grading

HEMATOLOGY					
	Normal Range	Grade 1	Grade 2	Grade 3	Grade 4 (SAE)
Hemoglobin<br>Male and female	8.4 – 12.4 g/dL[a]	7.5 – 8.3g/dL	6.1 – 7.4 g/dL	5.0 – 6.0 g/dL	< 5.0 g/dL

Platelets	133,000 – 523,000/mm^3 [a]	75,000 - 132,999/mm^3	50,000 - 74,999/mm^3	20,000 - 49,999/mm^3 or > 1,000,000/mm^3	<20,000/mm^3
WBCs	5,300 – 15.300/mm^3 [a]	15,299 – 16,500/ mm^3 or 3,501-5,299/mm^3	16,501-18,000 /mm^3 or 2,001-3,500/mm^3	18,001-30,000/mm^3 or 1,000-2,000/mm^3	>30,000 or <1,000 /mm^3
Absolute Lymphocyte Count	2,300-9,500/mm^3 [b]	1,000-2,399/mm^3	750-999/mm^3	500-749/mm^3	<500/mm^3
**CHEMISTRIES**					
	Normal Range	Grade 1	Grade 2	Grade 3	Grade 4 (SAE)
Creatinine	0.2–0.5 mg/dL (15.2–44.2 µmol/L)[c]	44.2 – 91.5 µmol/L (0.5-1.0 mg/dL)	91.6 – 183 µmol/L (1.1-2.0 mg/dL)	183.1 – 366 µmol/L (2.1-4.0 mg/dL)	> 366 µmol/L (>4.1 mg/dL) or dialysis required
ALT	3.9 – 49.6 U/L[a]	49.7-124 U/L	124.1 – 248 U/L	248.1 – 496 U/L	> 496 U/L

[a]Determined and adapted from normal children in Donéguébougou, a rural Malian village and malaria vaccine trial site
[b] From Lugada, E. S. et al. Population-Based Hematologic and Immunologic Reference Values for a Healthy Ugandan Population. *Clin Diag Lab Immunol*, 11, 29-34.
[c] From Current Pediatric Diagnosis & Treatment, 17th edition, 2005.

10.4 Assessment of causality

Every effort will be made by the investigator to explain each adverse event and assess its causal relationship, if any, to administration of the study vaccine(s).

The degree of certainty with which an adverse event can be attributed to administration of the study vaccine(s) (or alternative causes, e.g., natural history of the underlying diseases, concomitant therapy, etc.) will be determined by how well the event can be understood in terms of one or more of the following:

- Reaction of similar nature having previously been observed with this type of vaccine and/or formulation.

- The event having often been reported in literature for similar types of vaccines.

- The event being temporally associated with vaccination or reproduced on re-vaccination.

All solicited local (injection site) reactions will be considered causally related to vaccination. The investigators will assess the causality of all other adverse events using the following method:

In your opinion, did the vaccine(s) possibly contribute to the adverse event?

NO : The adverse event is not causally related to administration of the study vaccine(s). There are other, more likely causes and administration of the study vaccine(s) is not suspected to have contributed to the adverse event.

YES : There is a reasonable possibility that the vaccine contributed to the adverse event.

If an AE is determined not to be causally related to administration of a study vaccine, the investigators will specify the likely cause if one can be determined.

10.5 Following-up of adverse events and assessment of outcome

Investigators will follow up subjects with serious adverse events until the event has resolved or until the condition has stabilized regardless of when this outcome occurred in relation to the study conclusion. Investigators will follow up participants with non-serious adverse events until the participant completes the study. Clinically significant laboratory abnormalities, as well as any adverse event, will be followed up until they have returned to normal, or until a satisfactory explanation has been provided. Reports relative to the subsequent course of an adverse event noted for any subject will be submitted to the Study Monitor.

Outcome will be assessed as Resolved, Resolved with Sequelae, Ongoing, or Death.

10.6 Clinical malaria

Clinical malaria will be recorded in the same fashion as other adverse events. In general, uncomplicated malaria will constitute an AE and severe malaria will constitute an SAE. Formal clinical case definitions of these conditions will not be used and study clinicians may exercise their judgment to hospitalize cases that do not meet standard definitions of severe malaria.

10.7 Serious adverse events

In 2004 the under-5 year age group mortality rate in Mali was 21.9%. While the children participating in this study will receive better medical care than most Malian children do, some hospitalizations and even deaths are likely in the normal course of events in this population.

10.7.1 Definition of a serious adverse event

A serious adverse event is any untoward medical occurrence that results in death, is life threatening, results in persistent or significant disability/incapacity, requires in-patient hospitalization or prolongation of existing hospitalization or is a congenital anomaly/birth defect in the offspring of a study subject. In addition, important medical events that may jeopardize the participant or may require intervention to prevent one of the other outcomes listed above will be considered serious.

- Life threatening—definition: An adverse event is life threatening if the participant was at risk of death at the time of the event; it does not refer to an event, which hypothetically might have caused death, if it were more severe.

- Disabling/incapacitating—definition: An adverse event is incapacitating or disabling if the event results in a substantial disruption of the participant's ability to carry out normal life functions. This definition is not intended to include experiences of relatively minor medical significance such as headache, nausea, vomiting, diarrhea, influenza, and accidental trauma (e.g. sprained ankle).

- Hospitalization: In general, hospitalization signifies that the participant has been detained (usually involving at least an overnight stay) at the hospital or emergency ward for treatment that would not have been appropriate in the physician's office or outpatient setting.

10.7.2 Reporting serious adverse events

All serious adverse events will be:

- Recorded on the appropriate serious adverse event case report form
- Followed through resolution by a study physician
- Reviewed by a study physician

Any AE considered serious by the Principal Investigator or Subinvestigator or meets the aforementioned criteria must be submitted on an SAE form to PPD Development, NIAID's pharmacovigilance contractor, which will notify USAMMDA as the Sponsor as well as DMID, USAID, GSKBio, and the University of Maryland International Regulatory Affairs Coordinator of any SAEs, with in the timelines required by each of these parties.

10.7.2.1 USAMMDA SAE REPORTING CONTACT INFORMATION

U.S. Army Medical Research and Materiel Command / U.S. Army Medical Materiel Development Activity Regulatory Affairs Office:

- E-mail USAMRMCREGAFFAIRS@amedd.army.mil (Preferred method)
- facsimile (301-619-0197) or
- telephone (301-619-0317) – Provide voice mail message during non-duty hours

USAMMDA will notify the HSRRB of any SAEs.

10.7.2.2 PPD SAE REPORTING CONTACT INFORMATION

Medical Affairs/Pharmacovigilance
PPD Development
3151 17th St.
Wilmington, NC 28412
SAE Fax line: 888 488-9697

Email: dmidpvg@wilm.ppdi.com

Questions about SAE reporting may be referred to the SAE Hotline (available 24 hours a day/7 days a week) at 800 201-8725

PPD will notify USAMMDA as the Sponsor, DMID, GSKBio, USAID, and the University of Maryland International Regulatory Affairs Coordinator of any SAEs.

10.7.2.3 GSKBio SAE REPORTING CONTACT INFORMATION

Study Contact at GSK Biologicals for Reporting Serious Adverse Events Manager Clinical Safety Vaccines	Clinical Development Manager
GSK Biologicals Clinical Safety Physician,<br>GlaxoSmithKline Biologicals,<br>Rue de l'Institut 89, 1330 Rixensart,<br>Belgium.<br>Tel: +32.2.656.87.98<br>Fax: +32.2.656.80.09<br>Mobile phone for 7/7 day availability:<br>+32.477.40.47.13<br>email: rix.ct-safety-vac@gskbio.com	Amanda Leach,<br>Clinical Development Manager,<br>GlaxoSmithKline Biologicals,<br>Rue de l'Institut 89, 1330 Rixensart,<br>Belgium.<br>Tel: +32.2.656.77.88<br>Fax: +32.2.656.80.44<br>email: amanda.leach@gskbio.com

SAEs will also be reported within 24 hours by telephone, fax or E-mail to the Local Medical Monitor and the FMPOS Ethics Review Committee in Mali.

10.7.2.4 HSRRB SAE REPORTING CONTACT INFORMATION

Unanticipated problems involving risk to volunteers or others, serious adverse events related to participation in the study, and all volunteer deaths should be promptly reported by telephone (301-619-2165), by e-mail (hsrrb@amedd.army.mil), or by facsimile (301-619-7803) to the U.S. Army Medical Research and Materiel Command's Human Subjects Research Review Board. A complete written report should follow the initial notification. In addition to the methods above, the complete report can be sent to the U.S. Army Medical Research and Materiel Command, ATTN: MCMR-ZB-P, 504 Scott Street, Fort Detrick, Maryland 21702-5012.

10.7.2.5 REGULATORY REPORTING

Following notification via PPD from the investigator, USAMMDA, the IND sponsor, will report events that are both serious and unexpected and that are associated study product(s) to the FDA within the required timelines as specified in 21 CFR 312.32: fatal and life threatening events within 7 calendar days (by phone or fax) and all other serious adverse events in writing within 15 calendar days. All serious events designed as "not associated" to study product(s), will be reported to the FDA at least annually in a summary format.

10.7.2.6 SAE reporting procedures

The study clinician will complete a Serious Adverse Event Form within the following timelines:

- All deaths, whether related or unrelated, will be recorded on the Serious Event Form and sent by fax or electronic mail immediately upon discovery of the death regardless of availability of complete information. For initial reports provided via telephone, a written report via electronic mail or fax will be provided within 24 hours of discovery of death by investigators. Follow-up reports will be provided as complete or additional relevant information is available.

- Serious adverse events other than death, regardless of relationship, will be reported via fax by the site immediately upon discovery of the experience regardless of availability of complete information. For initial reports provided via telephone, provide a written report via electronic mail or facsimile within 72 hours of becoming aware of the event. Follow-up reports will be provided as complete or additional relevant information is available.

Other supporting documentation of the event may be requested by the pharmacovigilance contractor and should be provided as soon as possible.

All SAEs will be followed until satisfactory resolution or until the Principal Investigator or Subinvestigator deems the event to be chronic or the patient to be stable.

Every serious adverse event that is not resolved at the time the initial written report is filed will have a follow-up report submitted when information is available. Any submitted report will be identified as “initial”, “follow-up”, or “medical monitor”.

The initial notification will include:

- The study protocol number and the name of the PI
- The participant ID number, gender and age
- The date of onset of the SAE, and date of administration of study vaccine(s)
- Description of event

The PI will not wait to collect additional information to fully document the event before making notification of a serious adverse event. The telephone/e-mail report will be followed by a full written report using the SAE form within the CRF, detailing relevant aspects of the adverse events in question.

Formal surveillance will continue until stability or resolution of the event. However, in the event that instances of death or cancer in a study participant are brought to the attention of the investigator AT ANY TIME after cessation of the study AND suspected by the Investigators to be related to study medication will be reported to USAMMDA, DMID and GSKBio within four weeks of coming to the knowledge of the PI.

10.8 Treatment of adverse events

The investigators will provide treatment of adverse events. Advice will be sought from the Local Medical Monitor and other medical specialists as indicated for severe, unusual or complicated medical conditions. Treatment events will be fully documented. Clinical trials insurance will cover medical liability of Malian clinical investigators.

10.9 Halting Rules

Because this Phase 1 study is not powered for statistically significant comparisons of adverse event frequencies between groups and because the DSMB will be conducting safety reviews before each dose escalation, statistical halting rules will not be used. Decisions to halt or pause the study will be made by USAMMDA and DMID based on the recommendations of the DSMB. In the event that one or more serious adverse events probably or suspected of being related to vaccination are detected following any immunization in any of the vaccine groups, no further vaccinations will be administered until a written report has been submitted to USAMMDA, DMID, GSKBio, the DSMB, the US Army HSRRB, the University of Maryland IRB, and the FMPOS Ethics Review Committee and the investigators have conferred with the Local Medical Monitor and the DSMB. DMID and USAMMDA retain the authority to halt or pause the study based on recommendations of the DSMB.

However, participants in the first cohort will be divided into half by age. The older half (i.e. the older ten of the 20 children in Cohort 1) will be immunized on one day and the younger half on the next day. To account for possible deferral of immunization due to acute illness or other reasons, at least 80% (8 children) of the older half of Cohort 1 will be immunized before proceeding to immunize any of the younger half of the cohort. No statistical safety evaluation will be performed based on these small subgroups. The intent is to be able to identify any very common, unexpected, severe events that would warrant immediate discussions with DMID, USAMMDA and the DSMB before immunizing the younger participants.

A single vaccine-related SAE in any participant of any cohort at any time within the first seven days of observation (study days 0-7) or a 20% rate of SAEs (related or unrelated) in a cohort during the 7 day surveillance period following immunization will result in a temporary pause in immunizations and consultation with USAMMDA, DMID and the DSMB.

10.10 Safety Oversight

10.10.1 Local Medical Monitor

A qualified Malian pediatrician will serve as the Local Medical Monitor (LMM) for this study. The LMM's *curriculum vitae* will be maintained on record. She is a qualified and experienced pediatrician not otherwise associated with this protocol, who is able to provide medical care to research subjects for conditions that may arise during the conduct of the study. If safety concerns are identified the LMM may request a meeting of the DSMB to review safety data. The medical monitor is required to review all unanticipated problems involving risk to subjects or others, SAEs, and all subject deaths

associated with the protocol and provide an unbiased written report of the event. For SAEs and deaths she will provide an unbiased written report of the event within 10 calendar days of the initial report. At a minimum, the medical monitor should comment on the outcomes of the event or problem and, in the case of an SAE or death, comment on the relationship to participation in the study. The medical monitor should also indicate whether he/she concurs with the details of the report provided by the study investigator. Reports for events determined by either the investigator or medical monitor to be possibly or definitely related to participation and reports of events resulting in death should be promptly forwarded to the sponsors and the IRBs through the DMID contractor PPDi.

The involvement of the LMM will be particularly important when decisions related to safety of participants have to be made quickly. A copy of treatment codes will be in her safekeeping and she may unblind individual study participants if deemed necessary for medical and/or ethical reasons (for example, in the case of a participant in urgent need of rabies immunization). In exceptional circumstances, for example a death possibly related to vaccination, she would have the authority to temporarily suspend the whole or any specific aspect of the trial pending review by the USAMMDA and DMID Medical Monitors and the DSMB.

The LMM will be on-site during active phases of immunization and during the immediate post-vaccination surveillance period. A physician on staff at the Bandiagara Health Center who resides full time in Bandiagara will act as the on-site LMM in support of the LMM between the vaccinations.

The LMM's role will include:

- Acting as the study participants' advocate
- Providing advice to the investigators on whether a set of clinical circumstances in a study warrants formal notification to the DMID and USAMMDA Medical Monitors and the DSMB.
- Providing clinical advice on any illness in study subjects especially in circumstances in which treatment might influence the course of the trial.
- Review all SAEs as outlined above

The LMM will liaise closely with the PI throughout the course of the trial and relay relevant safety information to the PI. The PI and co-PI will subsequently inform the FMPOS IRB, the DSMB, DMID, GSKBio, WRAIR, USAMMDA and USAID of safety concerns that arise.

10.11 Data Safety and Monitoring Board (DSMB)

An independent DSMB will be constituted by DMID to review safety data at specified intervals prior to each dose escalation. The role of the DSMB is to provide safety oversight over the conduct of the trial. It will review blinded safety data before each dose escalation and advise on progression to the next dose level. The DSMB will hold two conference calls to review the safety data generated from the trial up to that point. These calls will occur as soon as possible after the completion of the 7-day

surveillance period following each of the first 2 immunizations of cohorts 1 and 2. The first call will thus occur approximately one week before the first immunization with the 25 μg dose level of FMP2.1, and the second will occur approximately one week before the first immunization with the 50 μg dose level (Figure). The purpose of these conference calls will be to review the accumulated safety data to advise whether or not the study can proceed with the dose escalation, and whether the trial can proceed generally. In the event that the DSMB identifies a safety concern, the collaborative group including the investigators, DMID, WRAIR, GSK and USAID will review all safety data to decide whether to give the next sequential dose. Senior investigators and representatives from USAMMDA, DMID, WRAIR, GSK and USAID will be invited to but not obligated to participate in the conference calls during open sessions, which will be followed by closed sessions including only DSMB members.

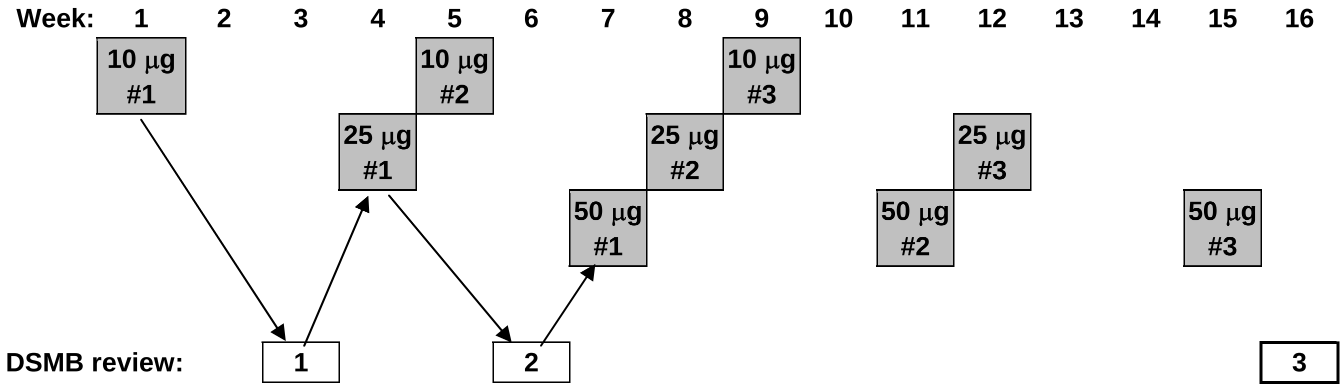

Figure. Timeline for safety reviews by DSMB. Arrows down indicate immunization safety data included in safety review. Arrows up indicate review results in recommendation to proceed with immunization.

The DSMB will meet after the 7-day surveillance period following the last immunization of cohort 3 to review safety data and provide advice to the sponsors and investigators with regard to dose selection for the subsequent Phase 2 trial.

The investigator will inform the DSMB of:

- All subsequent protocol amendments, informed consent form changes or revisions of other documents originally submitted for review
- Serious adverse events (SAEs), grade 3 adverse experiences and grade 2 abnormalities of laboratory tests occurring during the study, regardless of relationship to the study vaccine
- New information that may affect adversely the safety of the subjects or the conduct of the study.

The DSMB may recommend that the Sponsor, USAMMDA, in conjunction with DMID, put the study vaccinations on temporary hold pending review of potential safety issues. The DSMB will request

additional information from the Principal Investigator as needed and will request any appropriate statistical calculations to support discussions with the Sponsor, USAMMDA, in conjunction with DMID. All documentation provided to members of the DSMB for information and review will be treated in a confidential manner.

11 CLINICAL MONITORING

11.1 Site Monitoring Plan

Site monitoring will be conducted by DMID or its designated monitoring contractor, to ensure that GCP standards and regulatory guidelines are being followed. Pre-trial monitoring visits will be made to the site, including the clinical laboratory. All records will be made available to monitors, including regulatory files, CRFs and other source documents, QA/QC documentation, SOPs, etc. At the discretion of the monitor, additional site visits may be made during the course of the trial and at the end of the surveillance period.

In addition, monitors from USAMMDA and GSKBio may make site visits, in coordination with the primary monitoring group designated by DMID. DMID or its designated monitoring contractor and monitors from USAMMDA and GSKBio should coordinate their visits in order to reduce the burdens to the clinical study site, clinical investigation staff and study participants.

In conjunction with the monitoring body designated by DMID, a detailed monitoring plan, subject to approval by OCRA, will be developed and included in the Manual of Procedures. The monitoring plan will include the number of subject charts to be reviewed, which/what proportion of data fields and what will be monitored, and who will be responsible for conducting the monitoring visits, and who will be responsible for ensuring that monitoring findings are addressed.

12 STATISTICAL CONSIDERATIONS

Data entry will be performed on site and in the MRTC/MMVDU data management unit in Bamako if necessary. The EMMES Corporation will do data analysis and reporting for primary and secondary endpoints in collaboration with the MRTC Data Management Unit.

12.1 Primary endpoints

- Occurrence of solicited symptoms during a 7-day surveillance period after vaccination (day of vaccination and study days 1, 2, 3 and 7).
- Occurrence of unsolicited symptoms during a 30-day surveillance period after vaccination.
- Occurrence of serious adverse events during the study period.

12.2 Secondary endpoints

- Titers and activity of anti-FMP2.1 antibody at each time point where serology samples are analyzed, measured by ELISA*, by process inhibition assay and growth inhibition assay, (in this order of priority when serum quantities are limited).
- Cellular immune responses at each time point
- Incidence of unique haplotypes of AMA-1 domain I in *P. falciparum* infections detected during follow-up

*The antibody response to FMP2.1 by ELISA will be the main measure of immunogenicity for consideration in dose selection for efficacy trials.

12.3 Study cohorts/datasets to be evaluated

Total cohort

The 'Total Cohort' will include all participants enrolled (defined as randomized to study groups) in the study.

Safety cohort

The 'Safety Cohort' will consist of all participants who have received at least one dose of study vaccine or comparator and for whom any data on safety are available.

The presentation of safety data will explore separately the adverse experiences among participants who received all vaccinations, among those who received only some and among those with clinical violations of study protocol.

Immunogenicity cohort

The 'Immunogenicity Cohort' will include all evaluable participants (i.e., those meeting all eligibility criteria, and who have received at least one immunization with any of the study or control vaccines) for whom data concerning immunogenicity endpoint measures are available. This will include participants for whom assay results are available for antibodies against at least one study vaccine antigen component after vaccination.

12.4 Sample Size Considerations

The sample size is driven by the primary endpoint, safety. However, this Phase 1 trial is not powered to detect differences between groups. Even if comparative statistics for the safety variables are computed, the study will have low power to detect anything other than very large differences in the incidence of local and general side effects between the vaccination groups. This is done weighing the need to detect any possible untoward reactions against the need to limit the number of participants involved for safety purposes. The sample size of 30 in each study group is widely accepted and used in industry for the initial assessment of the safety, tolerance and immunogenicity of an investigational vaccine, including other blood-stage subunit protein malaria vaccines with the AS02A and other adjuvants. Incorporation of a comparator vaccine as control will enable broad initial estimates of the incidence of local and general side effects and of immune responses among vaccine recipients. Table 12 provides the probability of detecting events in the groups of 30 that will receive the 25 µg and 50 µg doses. For example, if the "true" rate of an SAE is 5%, then the probability of observing no events (Pr 0/30) in a group size of 30 is 0.21 and the probability of observing one or more events (Pr 1+/30) in a group size of 30 is 0.79.

Table 12. Event detection probability table.

Event Rate	Pr 0/30	Pr 1+/30
0.01	0.74	0.26
0.05	0.21	0.79
0.08	0.08	0.92
0.10	0.04	0.96

Event rate. True rate at which an event occurs.
Pr 0/30. Given the event rate, probability that no events will be detected among 30 vaccinees.
Pr 1+/30. Given the event rate, probability that one or more events will be detected among 30 vaccinees.

The inclusion of an initial group of 20 children randomized 3:1 to receive 10 μg of FMP2.1 in 0.1 mL of AS02A or control vaccine is for the purpose of assuring that there is not a common adverse reaction in children that should preclude further testing. This is not considered a dose level to be evaluated for further clinical development and thus does not warrant the larger sample size of the 25 μg and 50 μg dose level groups.

12.5 Final Analysis Plan

A final research analysis plan will be agreed upon by the investigators, USAMMDA, DMID, WRAIR, GSKBio and USAID prior to locking of the database for the final analysis. This plan will include information on adverse events by dose and number of vaccinations. The primary analysis will be conducted on data and samples collected until Day 90 (30 days post Immunization 3 for each cohort). The primary analysis will be used for decision-making related to the product clinical development plan. The study will continue in a single blind manner for additional safety surveillance and immunogenicity assessment. This additional information will be appended to the study report. It is anticipated that the results of this study will be presented to the scientific community via oral presentations at meetings and written publications in scientific journals. The data to be presented and the authorship will be discussed between partners prior to any official communication.

The official report of the primary analysis will be written by the study investigators and the statistical consultant and submitted through appropriate channels and upon approval by USAMMDA and DMID. This report will contain detailed information about the participants, their tolerance of the vaccines, their side effects and laboratory abnormalities, as well as their overall immune responses to immunization.

12.5.1 Analysis of demographics

Demographic characteristics (age, sex, and place of residence) of each study cohort will be tabulated. The mean age (plus range and standard deviation) by sex of the enrolled participants, as a whole and per group will be tabulated.

12.5.2 Analysis of immunogenicity

The primary immunogenicity endpoint of ELISA titers of FMP2.1 antibodies will be assessed in several ways. A series of graphs will display immunologic responses. For each vaccine group and time point, the distribution of anti-FMP2.1 antibody levels and reverse cumulative distribution curves will be plotted. Corresponding summary statistics will show means and standard deviations as well as median, 25th and 75th percentiles, and 10th and 90th percentiles. The results will be presented both as raw data and as log-transformed data.

In addition, for each treatment group and time point, anti-FMP2.1 antibody levels will be presented as geometric means of OD units with 95% confidence intervals. For each vaccine group and for each time point, a table will show the proportion of participants with two-fold, four-fold, and eight-fold increases in anti-FMP2.1 antibody titers relative to their pre-immunization titers.

12.5.3 Analysis of safety

The overall percentage of participants with at least one local adverse event (solicited or unsolicited) and the percentage with at least one general adverse event (solicited and unsolicited) during the 7-day surveillance period after vaccination will be tabulated. The incidence, intensity and relationship of individual solicited symptoms over the 7-day surveillance period will be calculated per group and vaccine administration.

The number of participants with at least one report of an unsolicited adverse event, classified by WHO-preferred terms, reported up to 30 days after vaccination will be tabulated per group and vaccine administration. The intensity and relationship to vaccination of the unsolicited symptoms reported will also be assessed.

Serious adverse events are expected to be rare, but where observed will be described. Comparisons between study groups of incidence of symptoms, local and general symptoms will be made based on a two-sided Fisher's Exact Tests. Analysis of safety during the 15-month surveillance period will consist of comparison of incidence of serious adverse events as well as hemoglobin, creatinine and ALT levels.

Clinical laboratory parameters

Hematological (CBC) and biochemical (ALT, creatinine) laboratory parameters will be measured at specific time points, Days 0, 7, 30, 37, 60, 67 and 90. Clinically relevant abnormal values based on reference intervals determined in rural Malian children will be tabulated and a trend analysis could be performed if deemed necessary. Laboratory abnormalities will be graded on a scale of 1-4 as defined in relevant SOPs.

12.5.4 Analysis of CMI responses

Correlation will be measured 1) between serum anti-AMA-1 antibody levels and T-helper cell responses; and 2) between avidity and/or classes and IgG sub-classes of anti-AMA-1 antibodies and T-helper cell responses. Correlations between continuous measures of immune response will be assessed using the standard Pearson as well as Spearman rank correlation coefficients, on $\log_{10}$ transformed data when a logarithmic transformation results in a distribution more nearly normal than the distribution of untransformed values. Association between a continuous and a categorical measure will be assessed using t-tests or analysis of variance, or the analogous tests on ranks. Association between categorical measures will be assessed using chi-square or exact tests. Appropriate summary descriptive statistics (e.g., means and standard deviations or medians and ranges) will be presented. Effects of covariates (e.g., gender, ethnicity) will be assessed using regression models.

12.5.5 Analysis of AMA-1 sequences

Based on previous studies of the incidence of malaria infection and disease in Bandiagara, we expect each participant to experience an average of two episodes of clinical malaria during the year

of the study (5). The parasite DNA from filter paper samples corresponding to these episodes will be subject to sequencing of domain 1 of AMA-1. As a first step, the frequency will be measured of haplotypes based on all 31 polymorphic amino acid residues seen in this region. Independently from this clinical trial, research is underway to identify smaller haplotypes based on fewer polymorphic amino acid residues, which may represent clusters of polymorphisms that are under natural immune selection. The sequence data will be analyzed with respect to these putative immunologically relevant haplotypes, with comparisons of their frequencies over time following immunization and of their incidence between malaria vaccine and control groups. Candidate "protective haplotypes" identified in this fashion will be assessed by determining whether the polymorphisms they include form clusters on the 3-dimensional face of the AMA protein that could plausibly correspond to antibody-binding sites.

These preliminary studies are expected to improve our understanding of how anti-AMA-1 antibodies may interact with the protein to block parasite invasion and how recombinant AMA-1 proteins might be designed to offer more universal protection. Identification of haplotypes based on fewer than 31 amino acid residues that appear to be under selection pressure by natural and/or vaccine-induced immunity will aid in designing sampling and analysis strategies to measure efficacy at the molecular level in the subsequent Phase 2 study. The preliminary molecular studies conducted on samples obtained from this Phase 1 trial are not designed to measure vaccine efficacy, and will not be construed as such.

13 SOURCE DOCUMENTS AND ACCESS TO SOURCE DATA/DOCUMENTS

Electronic CRFs (eCRFs) will be supplied by the EMMES Corporation under contract to the NIAID, and a remote data entry system will be used. Copies of the CRFs will be made available for Source Document Workbooks (SDWs). The SDW for each subject will be maintained at the site. All SDWs will be filled out completely by appropriate study personnel.

The site will permit authorized representatives of the USAMMDA and DMID, their designees, and appropriate regulatory agencies to examine (and when required by applicable law, to copy) clinical records for the purposes of quality assurance reviews, audits and evaluation of the study safety and progress. These representatives will be permitted access to all source data.

14 QUALITY CONTROL AND QUALITY ASSURANCE

SOPs for quality management will be developed, used to train appropriate personnel, and kept on file with documentation of training. Data will be evaluated for compliance with protocol and accuracy in relation to source documents. The study will be conducted in accordance with procedures identified in the protocol. The types of materials to be reviewed, who is responsible, and the schedule for reviews will be referenced in the SOPs. Study-specific training will be provided for all staff prior to the commencement of the trial.

The study will be conducted at a single center, the Bandiagara Malaria Project in Bandiagara, Mali, with the exception of immunological studies which will be conducted at WRAIR (ELISA, GIA, PIA), UMB (antibody avidity, CMI, sequencing) and the main campus of the University of Bamako (serology not to be used for study endpoints, and CMI).

SOPs will be used at all clinical and laboratory sites. Regular monitoring and an independent audit will be performed according to GCP/ICH (e.g., data monitoring). Following written standard operating procedures, the monitors will verify that the clinical trial is conducted and data are generated, documented (recorded), and reported in compliance with the protocol, GCP, and the applicable regulatory requirements. Reports will be submitted to DMID and USAMMDA on monitoring activities.

The investigational site will provide direct access to all trial related sites, source data/documents, and reports for the purpose of monitoring and auditing by the USAMMDA and DMID, and inspection by local and regulatory authorities.

The EMMES Corporation and the MRTC Biostatistics and Epidemiology Unit will implement quality control procedures beginning with the data entry system and generate data quality control checks that will be run on the database. Any missing data or data anomalies will be communicated to the field site for clarification/resolution.

15 ETHICS/PROTECTION OF HUMAN SUBJECTS

15.1 Ethical Standard

The WRAIR FMP2.1/AS02A vaccine has been submitted as FDA IND BB-11,140 and the study described in this protocol will be conducted according to current Good Clinical Practices (US 21 CFR Part 50-Protection of Human Subjects and Part 56-Institutional Review Boards, US Army Regulation AR 40-38 and AR 70-25, US 45 CFR 46, 21 CFR 312, the Declaration of Helsinki, and the applicable rules and regulations of Mali).

The FMPOS IRB (FWA00001769) will review and approve the protocol prior to study start. In addition, the study will be reviewed by the U.S. Army Medical Research and Materiel Command's Human Subjects Research Review Board (HSRRB), by DMID, and the University of Maryland IRB. Documentation of the approval by these ethical review boards will be kept in the PI's study file.

15.2 Institutional Review Board

All amendments will be submitted to the University of Bamako Faculty of Medicine Pharmacy and Odonto-stomatology (FMPOS) IRB, the UMB IRB, and the HSSRB as well as to USAMMDA and DMID. No amendments will go into effect without written approval from the FMPOS IRB, the UMD IRB, HSRRB, USAMMDA and DMID except when necessary to eliminate immediate hazards to the participants. Protocol deviations will also be reported to each IRB according to the policy of each IRB. CRFs and other source documents will be examined to determine whether missing data were not transcribed, unavailable or missing for unknown reasons and this information will be coded and documented in the database.

The investigators will inform all the IRBs, USAMMDA and DMID of the following:

- All subsequent protocol amendments, informed consent changes or revisions of other documents originally submitted for review
- Serious and/or unexpected adverse events occurring during the study, where required
- New information, including any provided by GSKBio, USAMMDA or WRAIR, that may affect adversely the safety of the participants or the conduct of the study
- An annual update and/or continuing review reports, as required
- A final report including SAE outcomes will be provided when the study has been completed.

15.3 Informed Consent Process

The principles of informed consent in the current edition of the Declaration of Helsinki will be implemented before any protocol-specified procedures or interventions are carried out.

Information will be given in both oral and written form whenever possible. The written consent documents will embody the elements of informed consent as described in the current edition of the Declaration of Helsinki, will adhere to the ICH Harmonised Tripartite Guideline for Good Clinical Practice and 45CFR46 and 21CFR50, and will also comply with applicable Malian regulations. The oral consent process will be consistent with 45CFR46.§46.117, 21CFR50.27 and ICH E6 (R1) Section 4.8. Independent witnesses will be used to attest that illiterate potential participants have understood the contents of the informed consent document.

15.3.1 Screening and study informed consent

We consider informed consent to be a dynamic, ongoing process, with continuous availability of investigators to answer any questions that arise in the course of the trial and to ensure that participants' parents/guardians understand trial procedures. Should new data become available that could affect participant safety and/or willingness to continue in the study, informed consent would be obtained and documented again.

The extensive contact between the team of investigators and the population of Bandiagara has led to the development of mutual trust and the establishment of an ongoing informed consent process attempting to address issues related to interventional studies in resource-limited settings. Many discussions with local community leaders, heads of families and citizens through group meetings, and more limited group interviews have reviewed the need to obtain a written informed consent from parents/guardians. The community has now become familiar with the informed consent process, including written, signed consent forms, which have been used for several studies at this site, including several interventional and observational pediatric trials and two Phase 1 adult malaria vaccine trials in 2003-2005.

Before starting any study in Bandiagara, the senior Malian and US investigators visit the local commandant (representative of the national government), the mayor, the director of the local school system, the chiefs of each of the eight quartiers of Bandiagara, the medical director of the local health center, the director of the Bandiagara Center for Research of Traditional Medicine, and the head of the Bandiagara traditional healers' association. These are courtesy visits during which results of the previous year's studies are summarized and plans for new studies are explained and any questions are answered. In accordance with the tradition in Mali, small quantities of kola nuts are given to the chiefs of the quartiers and the traditional healers as a sign of respect.

These individual meetings are followed by a larger community meeting attended by the above personages as well as numerous other local health care providers, traditional healers and notable citizens (including several respected women from the community). Planned studies are explained in more detail, and ample time is given for carefully and thoroughly addressing all questions and

concerns. This question and answer period is frequently prolonged with many detailed and often sophisticated questions being raised. Each presentation, question and response is translated from French into Dogon and Peulh so that all present understand the entire discourse.

Once this group of community leaders has expressed their approval of the planned study, they disseminate information to their various constituencies, so that when potential recruits approach the study staff they are already generally aware of the nature of the impending study. The investigators do not consider this process to constitute “community consent” in addition to or in lieu of individual informed consent, but rather a community “permission to enter” that is a necessary prerequisite to conducting any study in a tight-knit and highly organized traditional rural community such as Bandiagara.

After community meetings have been held, a brief announcement is made on local radio describing the study. Prior to initiating screening and obtaining informed consent, the study team meets to review the oral translation of the consent forms into the relevant local languages and dialects word by word, until there is consensus that the individuals responsible for giving consent in each language are conveying as accurately as possible the exact content of the IRB-approved French language consent form.

A period of approximately two weeks is allotted for screening and recruitment to allow plenty of time for participants’ parents/guardians to consider their decision about participating and to discuss their participation with family members and others in the community. At the times of screening and recruitment, the consent forms are read to parents/guardians who speak French, and translated orally into the language of choice of each parents/guardians. Dogon is the main language spoken in Bandiagara. It has no written form and is constituted of more than 12 dialects. Others languages used in the area include Bambara and Fulani, both of which do have written forms, although neither the local population nor the study staff use the written forms of these mainly oral languages. Most of the population is illiterate; therefore with the exception of those whose preferred language is French, informed consent will be administered by oral translation of the text in presence of a witness. In all cases, the investigator will give the parents/guardians ample opportunity to inquire about the details of the study and to ask any questions before dating and signing the consent forms, including the opportunity to take a copy of the consent form home to review with family members or others before returning on a later day with their decision. All illiterate individuals will have the study and consent forms explained to them point by point by the interviewer in the presence of a witness who will sign the consent form. Witnesses will have no association with the conduct of the study and will not be related to the study subject. Individual consent to participate in research studies is given freely, and is not subject to approval by village elders or others.

We will use a comprehension test developed for previous malaria vaccine Phase 1 trials at this site. Parents of participants must answer all questions on the comprehension exam correctly to be eligible for enrollment in the study. Study staff will use incorrect answers to identify those areas of the informed consent document that need further review with the person giving informed consent. The incorrectly answered questions will then be repeated to the participant after a review of the informed consent form. A final score of 100% will be required for the participant to be considered

eligible to continue the screening process. This test will be administered orally in the presence of a witness in the case of potential participants who cannot read.

Informed consent will be documented by the use of a written consent form approved by the IRBs and signed or thumbprinted and dated by the parent/guardian, and by the person who conducted the informed consent discussion. Thumb printing will be used for illiterate persons, who are expected to constitute the majority of parents/guardians. The consent form will be orally translated into native languages from the French written version of the consent form. Consent will be administered by a study clinician who is fluent in French and will either be fluent in the local language of the parents/guardians or use a translator. A witness will assist during the procedure. After the participant's parent/guardian clearly states that she/he has understood what was explained and agrees for his/her child to participate to the study, the consent forms will be completed. The parent will be asked if she/he prefers to thumbprint or to sign. In the case of the thumbprint option, the distal end of her/his left thumb will be applied to a stamp inker and then firmly applied to the space on the consent forms reserved for thumbprints. This procedure has been followed for many years by the BMP team, and thumbprints obtained by trained staff following an SOP are uniformly legible.

The signature/thumbprint confirms that the consent is based on information that has been understood. Each participant's parents/guardians' signed informed consent form is kept on file by the investigator for possible inspection by regulatory authorities. The subject will receive a copy of the signed and dated written informed consent forms and any other written information provided by the investigator, and will receive copies of any signed and dated consent form updates and any amendments to the written information.

Since the vast majority of study participants' parents/guardians do not use telephones, fax or mail, contact information is provided in terms of local physicians who can be visited directly and who can themselves reach the investigators directly or by telephone or fax.

15.3.2 Screening recruitment radio announcement text

"The Bandiagara Research Project team from the Faculty of Medicine in Bamako has returned to Bandiagara, and sends its greetings to the population of Bandiagara. The team is here to test an experimental malaria vaccine, to see if it is safe to use in children who live in a place where they get malaria. Parents of boys and girls aged 1-6 years who live in Bandiagara town and are interested in having their child participate in this research study are invited to come to the Bandiagara Health Center at [*time*] on [*date*] to learn more about this study**."**

15.3.3 Compensation

To compensate the participant's family for participating in the study, each will be given 50 kg rice and 50 kg millet, worth about $60. They will receive half of the rice and millet after the first shot and the other half at the end of the study. The annual per capita income for Mali as a whole is estimated to be $250, with a more recent estimate of $600. However, these figures are based on large

segments of the population who live in rural areas with virtually no cash economy, and per capita income in Bandiagara is higher than this. Income data specifically for Bandiagara are not available, but Bandiagara is a relatively affluent town by Malian standards, with a lively market economy and a significant tourist trade. As the primary IRB for our studies in Bandiagara since 1997, the Mali IRB reviews and sets compensation amounts for specific settings in Mali based on local considerations with the aim of providing adequate compensation for time away from work and accepting risks of study participation while avoiding coercion.

15.4 Subject Confidentiality

Subject confidentiality is held strictly in trust by the participating investigators, their staff, DMID and USAMMDA and their agents. Confidentiality is extended to cover testing of biological samples and genetic tests in addition to the clinical information relating to participating subjects.

The study protocol, documentation, data, and all other information generated will be held in strict confidence. No information concerning the study or the data will be released to any unauthorized third party without prior written approval of USAMMDA and DMID.

The study monitor or other authorized representatives of USAMMDA and DMID may inspect all documents and records required to be maintained by the investigator, including but not limited to, medical records (office, clinic, or hospital) and pharmacy records for the subjects in this study. The clinical study site will permit access to such records.

Participants will be assigned a unique participant ID number. All results will be keyed to this number. Study records will only be available to staff members and will be kept locked at the study site conforming to the investigators' SOPs. Following the conclusion of the study, all records will be maintained on site for a minimum of two years, after which they will be stored long-term in the MMVDU data storage facilities in Bamako. All records will be retained in locked metal boxes for at least two years after a marketing application is approved for FMP2.1; or, if an application is not approved for FMP2.1, until two years after shipment and delivery of the drug for investigational use is discontinued and the FDA has been so notified. After either of these conditions have been met with permission of USAMMDA and DMID, records will be destroyed. Representatives of the US Army Medical Research and Materiel Command (USAMRMC), the FDA, USAMMDA and DMID may review these records.

15.5 Study Discontinuation

In the event that the study is discontinued before all three doses of the immunizations are administered, completion of the rabies vaccine series will be offered to participants who received one or more doses of the rabies vaccine. Depending on the reason for study discontinuation, rabies vaccine may be offered to participants who received one or more doses of the FMP2.1/AS02A malaria vaccine. If it is felt by the investigators and DMID and/or USAMMDA that immunization with rabies vaccine may interfere with further safety assessment, rabies vaccine may be deferred indefinitely for any individual in the malaria vaccine group.

At the end of the study all participants will be informed through their parents/guardians of the vaccine they received. Participants randomized to the FMP2.1/AS02A vaccine will be offered rabies immunization at that time. These immunizations will be done at the recommended schedule of 0, 7 and 21 days.

15.6 Future Use of Stored Specimens

If residual sera and cells are available following the serological and CMI assays described in this protocol, additional immunological and in vitro studies may be performed at the University of Bamako, University of Maryland or WRAIR on those samples for which permission was expressly granted for preserving samples for future studies at the time of informed consent at study enrollment. These assays may include anti-AMA-1 epitope mapping, determination of response to other allelic forms of the AMA-1 gene, or the ability of participant sera to interfere with in vitro parasite growth or invasion in an antigen-specific fashion. Additional research questions to be asked for cells include antigen-specific cytokine induction as measured by ELISPOT, flow cytometry, or both, or additional analysis to determine specificity of lymphoproliferation responses to this particular allele of AMA-1. These immunological studies will be limited to immune responses to malaria antigens unless specific permission for additional studies is obtained from the relevant IRBs. Samples from participants whose parents/guardians did not grant permission to preserve samples will be discarded after the primary and secondary analyses described in this protocol have been completed. Study participants will have the right to withdraw their permission for further use of their samples at any time during and after the study.

15.7 Justification for conducting the study in children

Infants and young children are the target population for a malaria vaccine in Africa, as they bear the main burden of malaria disease and death. Older children and adults living in areas of heavy transmission, such as Bandiagara, Mali, gradually acquire partial immunity that protects them against the clinical manifestations of malaria infection. This Phase 1 trial in children is an essential step toward the establishment of efficacy in the target age group in Phase 2 pediatric trials which will in turn need to be followed by Phase 3 pivotal efficacy trials in infants and young children. Malaria vaccine efficacy cannot be tested in older children or adults in this setting because of their partial immunity. The vaccine was tested first in adults mainly for safety reasons. This clinical development pathway follows that of other malaria vaccines that have been tested in several other African countries, most notably the RTS,S vaccine that underwent Phase 1 and 2 pediatric trials in Mozambique and another blood stage malaria vaccine that has recently been in Phase 1 and 2 pediatric trials in young children in Kenya.

15.8 Justification for exclusion

The study design evaluates a pediatric population. Therefore adults, including women, are excluded.

16 DATA HANDLING AND RECORD KEEPING

The investigator is responsible to ensure the accuracy, completeness, legibility, and timeliness of the data reported. All source documents should be completed in a neat, legible manner to ensure accurate interpretation of data. Black ink is required to ensure clarity of reproduced copies. When making changes or corrections, cross out the original entry with a single line, and initial and date the change. DO NOT ERASE, OVERWRITE, OR USE CORRECTION FLUID OR TAPE ON THE ORIGINAL.

Copies of the eCRF will be provided for use as source documents and maintained for recording data for each subject enrolled in the study. Data reported in the eCRF derived from source documents should be consistent with the source documents or the discrepancies should be explained.

DMID and/or its designee will provide guidance to investigators on making corrections to the source documents and eCRF.

A copy of the cleaned and locked eCRF will be provided to DMID and USAMMDA at the end of the study.

16.1 Data Management Responsibilities

All source documents and laboratory reports must be reviewed by the clinical team and data entry staff, who will ensure that they are accurate and complete. Adverse events must be graded, assessed for severity and causality, and reviewed by the site principal investigator or designee.

Data collection is the responsibility of the clinical trial staff at the site under the supervision of the site principal investigator. During the study, the investigator must maintain complete and accurate documentation for the study.

The EMMES Corporation will serve as the Statistical and Data Coordinating Center for this study and in collaboration with the MRTC data management unit will be responsible for data management, quality review, analysis, and reporting of the study data.

16.2 Data Capture Methods

Clinical data (including AEs, concomitant medications, and reactogenicity data) and clinical laboratory data will be entered into a 21 CFR Part 11-compliant IDES provided by The EMMES Corporation. The data system includes password protection and internal quality checks, such as automatic range checks, to identify data that appear inconsistent, incomplete, or inaccurate. Clinical data will be entered directly from the source documents.

16.3 Types of Data

Data for this study will include safety and outcome measures (e.g., reactogenicity, immunogenicity, virology).

16.4 Study Records Retention

Study documents should be retained for a minimum of 2 years after the last approval of a marketing application in an ICH region and until there are no pending or contemplated marketing applications in an ICH region or at least 2 years have elapsed since the formal discontinuation of clinical development of the investigational product. These documents should be retained for a longer period, however, if required by local regulations. No records will be destroyed without the written consent of USAMMDA and DMID, if applicable. It is the responsibility of USAMMDA and DMID to inform the investigator when these documents no longer need to be retained.

16.5 Protocol Deviations

A protocol deviation is any noncompliance with the clinical trial protocol, GCP, or MOP requirements. The noncompliance may be either on the part of the subject, the investigator, or the study site staff. As a result of deviations, corrective actions are to be developed by the site and implemented promptly.

These practices are consistent with ICH E6:

4.5 Compliance with Protocol, sections 4.5.1, 4.5.2, and 4.5.3

5.1 Quality Assurance and Quality Control, section 5.1.1

5.20 Noncompliance, sections 5.20.1 and 5.20.2.

It is the responsibility of the site to use continuous vigilance to identify and report deviations within 5 working days of identification of the protocol deviation, or within 5 working days of the scheduled protocol-required activity. All deviations must be promptly reported to DMID and USAMMDA via The EMMES Corporation’s IDES.

Any deviations that impact subject safety, or that alter the risk:benefit analysis or the scientific integrity of the study will be reported to the DMID, USAMMDA and the IRBs within two business days of the PI or study personnel becoming aware of the deviation.

All deviations from the protocol must be addressed in study subject source documents. A completed copy of the DMID Protocol Deviation Form (IDES form) must be maintained in the regulatory file, as well as in the subject’s source document. Protocol deviations must be sent to the local IRB per their guidelines. The site principal investigator/study staff is responsible for knowing and adhering to their IRB requirements.

17 PUBLICATION POLICY

Following completion of the study, the results of this research will be published in a scientific journal. The trial will be registered in a public trials registry such as ClinicalTrials.gov, which is sponsored by the National Library of Medicine. It is the responsibility of DMID to register this trial in an acceptable registry.

18 LITERATURE

(1) Snow RW, Craig M, Deichmann U, Marsh K. Estimating mortality, morbidity and disability due to malaria among Africa's non-pregnant population. Bull World Health Organ **1999**;77(8):624-40.

(2) Alonso PL, Armstrong JR, Lindsay SW. Malaria, bednets, and mortality. Lancet **1991**;338(8771):897.

(3) Nevill CG, Some ES, Mung'ala VO, et al. Insecticide-treated bednets reduce mortality and severe morbidity from malaria among children on the Kenyan coast. Trop Med Int Health **1996**;1(2):139-46.

(4) D'Alessandro U, Olaleye BO, McGuire W, et al. Mortality and morbidity from malaria in Gambian children after introduction of an impregnated bednet programme. Lancet **1995**;345(8948):479-83.

(5) Coulibaly D, Diallo DA, Thera MA, et al. Impact of preseason treatment on incidence of falciparum malaria and parasite density at a site for testing malaria vaccines in Bandiagara, Mali. Am J Trop Med Hyg **2002**;67(6):604-10.

(6) Lyke KE, Dicko A, Kone A, et al. Incidence of severe Plasmodium falciparum malaria as a primary endpoint for vaccine efficacy trials in Bandiagara, Mali. Vaccine **2004**;22(23-24):3169-74.

(7) Cerami C, Frevert U, Sinnis P, et al. The basolateral domain of the hepatocyte plasma membrane bears receptors for the circumsporozoite protein of Plasmodium falciparum sporozoites. Cell **1992**;70(6):1021-33.

(8) Vreden SG, Verhave JP, Oettinger T, Sauerwein RW, Meuwissen JH. Phase I clinical trial of a recombinant malaria vaccine consisting of the circumsporozoite repeat region of Plasmodium falciparum coupled to hepatitis B surface antigen. Am J Trop Med Hyg **1991**;45(5):533-8.

(9) Alonso PL, Sacarlal J, Aponte JJ, et al. Efficacy of the RTS,S/AS02A vaccine against Plasmodium falciparum infection and disease in young African children: randomised controlled trial. Lancet **2004**;364(9443):1411-20.

(10) Alonso PL, Sacarlal J, Aponte JJ, et al. Duration of protection with RTS,S/AS02A malaria vaccine in prevention of Plasmodium falciparum disease in Mozambican children: single-blind extended follow-up of a randomised controlled trial. Lancet **2005**;366(9502):2012-8.

(11) Good MF, Kaslow DC, Miller LH. Pathways and strategies for developing a malaria blood-stage vaccine. [Review] [94 refs]. Annual Review of Immunology **1998**;16:57-87.

(12) Narum DL, Thomas AW. Differential localization of full-length and processed forms of PF83/AMA-1 an apical membrane antigen of Plasmodium falciparum merozoites. Mol Biochem Parasitol **1994**;67(1):59-68.

(13) Crewther PE, Culvenor JG, Silva A, Cooper JA, Anders RF. Plasmodium falciparum: two antigens of similar size are located in different compartments of the rhoptry. Exp Parasitol **1990**;70(2):193-206.

(14) Waters AP, Thomas AW, Deans JA, et al. A merozoite receptor protein from Plasmodium knowlesi is highly conserved and distributed throughout Plasmodium. J Biol Chem **1990**;265:17974-9.

(15) Kocken CH, van der Wel AM, Dubbeld MA, et al. Precise timing of expression of a Plasmodium falciparum-derived transgene in Plasmodium berghei is a critical determinant of subsequent subcellular localization. J Biol Chem **1998**;273(24):15119-24.

(16) Deans JA, Alderson T, Thomas AW, Mitchell GH, Lennox ES, Cohen S. Rat monoclonal antibodies which inhibit the in vitro multiplication of Plasmodium knowlesi. Clin Exp Immunol **1982**;49(2):297-309.

(17) Thomas AW, Deans JA, Mitchell GH, Alderson T, Cohen S. The Fab fragments of monoclonal IgG to a merozoite surface antigen inhibit Plasmodium knowlesi invasion of erythrocytes. Mol Biochem Parasitol **1984**;13(2):187-99.

(18) Peterson MG, Marshall VM, Smythe JA, et al. Integral membrane protein located in the apical complex of Plasmodium falciparum. Mol Cell Biol **1989**;9(7):3151-4.

(19) Waters AP, Thomas AW, Deans JA, et al. A merozoite receptor protein from Plasmodium knowlesi is highly conserved and distributed throughout Plasmodium. J Biol Chem **1990**;265(29):17974-9.

(20) Marshall VM, Zhang L, Anders RF, Coppel RL. Diversity of the vaccine candidate AMA-1 of Plasmodium falciparum. Mol Biochem Parasitol **1996**;77(1):109-13.

(21) Oliveira DA, Udhayakumar V, Bloland P, et al. Genetic conservation of the Plasmodium falciparum apical membrane antigen-1 (AMA-1). Mol Biochem Parasitol **1996**;76(1-2):333-6.

(22) Hodder AN, Crewther PE, Matthew ML, et al. The disulfide bond structure of Plasmodium apical membrane antigen-1. J Biol Chem **1996**;271(46):29446-52.

(23) Thomas AW, Trape JF, Rogier C, Goncalves A, Rosario VE, Narum DL. High prevalence of natural antibodies against Plasmodium falciparum 83-kilodalton apical membrane antigen (PF83/AMA-1) as detected by capture-enzyme-linked immunosorbent assay using full-length baculovirus recombinant PF83/AMA-1. Am J Trop Med Hyg **1994**;51(6):730-40.

(24) Udhayakumar V, Kariuki S, Kolczack M, et al. Longitudinal study of natural immune responses to the Plasmodium falciparum apical membrane antigen (AMA-1) in a holoendemic region of malaria in western Kenya: Asembo Bay Cohort Project VIII. Am J Trop Med Hyg **2001**;65(2):100-7.

(25) Dolo A, Thera MA, Baby M. Strain-specific antibody responses to potential malaria vaccine antigens in Mali. Program and Abstracts of Annual Meeting of Am Soc Trop Med Hyg. **2003**. p. 495.

(26) Hodder AN, Crewther PE, Anders RF. Specificity of the protective antibody response to apical membrane antigen 1. Infect Immun **2001**;69(5):3286-94.

(27) Cortes A, Mellombo M, Mueller I, Benet A, Reeder JC, Anders RF. Geographical structure of diversity and differences between symptomatic and asymptomatic infections for Plasmodium falciparum vaccine candidate AMA1. Infect Immun **2003**;71(3):1416-26.

(28) Kennedy MC, Wang J, Zhang Y, et al. In vitro studies with recombinant Plasmodium falciparum apical membrane antigen 1 (AMA1): production and activity of an AMA1 vaccine and generation of a multiallelic response. Infect Immun **2002**;70(12):6948-60.

(29) Kocken CH, Withers-Martinez C, Dubbeld MA, et al. High-level expression of the malaria blood-stage vaccine candidate Plasmodium falciparum apical membrane antigen 1 and induction of antibodies that inhibit erythrocyte invasion. Infect Immun **2002**;70(8):4471-6.

(30) Healer J, Murphy V, Hodder AN, et al. Allelic polymorphisms in apical membrane antigen-1 are responsible for evasion of antibody-mediated inhibition in Plasmodium falciparum. Mol Microbiol **2004**;52(1):159-68.

(31) Plowe CV, Thera MA, Takala SL, et al. Dynamics of *Plasmodium falciparum* apical membrant antigen-1 sequence variation over three years at a malaria vaccine testing site in Bandiagara, Mali. Program and Abstracts of Annual Meeting of Am Soc Trop Med Hyg. **2005**. p. 277.

(32) Gordon DM, McGovern TW, Krzych U, et al. Safety, immunogenicity, and efficacy of a recombinantly produced Plasmodium falciparum circumsporozoite protein-hepatitis B surface antigen subunit vaccine. J Infect Dis **1995**;171(6):1576-85.

(33) Wu JY, Gardner BH, Murphy CI, et al. Saponin adjuvant enhancement of antigen-specific immune responses to an experimental HIV-1 vaccine. J Immunol **1992**;148(5):1519-25.

(34) Soltysik S, Bedore DA, Kensil CR. Adjuvant activity of QS-21 isomers. Ann N Y Acad Sci **1993**;690:392-5.

(35) Ribi E, Cantrell J, Feldner T. Microbiology. Washington, DC: American Society of Microbiology, **1986**.

(36) Myers KR, Truchot AT, Ward J. Cellular and molecular aspects of endotoxin reactions. In: Nowotny A, ed.Amsterdam: **1990**. p. 145-56.

(37) Loppnow H, Durrbaum I, Brade H, et al. Lipid A, the immunostimulatory principle of lipopolysaccharides? Adv Exp Med Biol **1990**;256:561-6.

(38) Johnson AG, Tomai MA. A study of the cellular and molecular mediators of the adjuvant action of a nontoxic monophosphoryl lipid A. Adv Exp Med Biol **1990**;256:567-79.

(39) Doherty JF, Pinder M, Tornieporth N, et al. A phase I safety and immunogenicity trial with the candidate malaria vaccine RTS,S/SBAS2 in semi-immune adults in The Gambia. Am J Trop Med Hyg **1999**;61(6):865-8.

(40) Bojang KA, Milligan PJ, Pinder M, et al. Efficacy of RTS,S/AS02 malaria vaccine against Plasmodium falciparum infection in semi-immune adult men in The Gambia: a randomised trial. Lancet **2001**;358(9297):1927-34.

(41) Bojang KA, Olodude F, Pinder M, et al. Safety and immunogenicty of RTS,S/AS02A candidate malaria vaccine in Gambian children. Vaccine **2005**;23(32):4148-57.

(42) Stoute JA, Gombe J, Withers MR, et al. Phase 1 randomized double-blind safety and immunogenicity trial of Plasmodium falciparum malaria merozoite surface protein FMP1 vaccine, adjuvanted with AS02A, in adults in western Kenya. Vaccine **2005**.

(43) Dreesen DW, Fishbein DB, Kemp DT, Brown J. Two-year comparative trial on the immunogenicity and adverse effects of purified chick embryo cell rabies vaccine for pre-exposure immunization. Vaccine **1989**;7(5):397-400.

(44) Nicholson KG, Farrow PR, Bijok U, Barth R. Pre-exposure studies with purified chick embryo cell culture rabies vaccine and human diploid cell vaccine: serological and clinical responses in man. Vaccine **1987**;5(3):208-10.

(45) Vodopija I, Sureau P, Lafon M, et al. An evaluation of second generation tissue culture rabies vaccines for use in man: a four-vaccine comparative immunogenicity study using a pre-exposure vaccination schedule and an abbreviated 2-1-1 postexposure schedule. Vaccine **1986**;4(4):245-8.

(46) Wasi C, Chaiprasithikul P, Chavanich L, Puthavathana P, Thongcharoen P, Trishanananda M. Purified chick embryo cell rabies vaccine. Lancet **1986**;1(8471):40.

(47) Cox JH, Schneider LG. Prophylactic immunization of humans against rabies by intradermal inoculation of human diploid cell culture vaccine. J Clin Microbiol **1976**;3(2):96-101.

(48) Kuwert EK, Marcus I, Werner J, Iwand A, Thraenhart O. Some experiences with human diploid cell strain-(HDCS) rabies vaccine in pre- and post-exposure vaccinated humans. Dev Biol Stand **1978**;40:79-88.

(49) Ajjan N, Soulebot JP, Stellmann C, et al. [Results of preventive rabies vaccination with a concentrated vaccine of the PM/WI38-1503-3M rabies strain cultured on human diploid cells. Preparation of mixed antirabies-antitetanus hyperimmune immunoglobulin by plasmapheresis of blood taken from vaccinated veterinary students]. Dev Biol Stand **1978**;40:89-100.

(50) Costy-Berger F. [Preventive rabies vaccination using vaccine prepared from human diploid cells]. Dev Biol Stand **1978**;40:101-4.

(51) Lyke KE, Diallo DA, Dicko A, et al. Association of intraleukocytic Plasmodium falciparum malaria pigment with disease severity, clinical manifestations, and prognosis in severe malaria. Am J Trop Med Hyg **2003**;69(3):253-9.

(52) Lyke KE, Burges R, Cissoko Y, et al. Serum Levels of the Proinflammatory Cytokines Interleukin-1 Beta (IL-1{beta}), IL-6, IL-8, IL-10, Tumor Necrosis Factor Alpha, and IL-12(p70) in Malian Children with Severe Plasmodium falciparum Malaria and Matched Uncomplicated Malaria or Healthy Controls. Infect Immun **2004**;72(10):5630-7.

(53) Djimde A, Doumbo OK, Cortese JF, et al. A molecular marker for chloroquine-resistant falciparum malaria. N Engl J Med **2001**;344(4):257-63.

19 SUPPLEMENTS/APPENDICES

Consent forms are attached as separate files.
